# Delayed cation dynamics enables dual-doped organic electrochemical transistors with high current sensitivity

**DOI:** 10.1038/s41467-026-73762-1

**Published:** 2026-06-01

**Authors:** Sen Zhang, Bingjun Wang, Nicholas Siemons, Iona Anderson, Shijie Wang, Yuxin Kong, Jin-Ting Ye, Xian-Kai Chen, Minning Wang, Xiao Yu, Chi-Yuan Yang, Yuxiang Li, Simone Fabiano, Jenny Nelson, Wei Ma

**Affiliations:** 1 State Key Laboratory for Mechanical Behavior of Materials, Xi’an Jiaotong University, Xi’an, China; 2 Department of Physics, Imperial College London, London, UK; 3 School of Materials Science and Engineering, Xi’an University of Science and Technology, Xi’an, China; 4 Institute of Functional Nano and Soft Materials (FUNSOM), Joint International Research Laboratory of Carbon-Based Functional Materials and Devices, Soochow University, Suzhou, China; 5 Laboratory of Organic Electronics, Department of Science and Technology, Linköping University, Norrköping, Sweden

**Keywords:** Electronic devices, Sensors and biosensors

## Abstract

The coupling between ionic and electronic species and their dynamic interplay lay the foundation for organic electrochemical transistors (OECTs) to transduce and amplify bio(chemical) signals through ion-modulated conductivity. However, the operation of most reported OECTs is typically dominated by single ions, e.g. anions for p-type accumulation devices, mainly due to the challenge of regulating ion dynamics to enable both types of ions to play a role during the electrochemical doping process. In this study, we propose that electrochemical doping of an OECT channel can occur via an anion-cation dual-doping mechanism, where cation expulsion and anion injection occur simultaneously. By designing a p-type organic mixed ionic-electronic conductor, Pu2gT, with strong side chain-cation interactions, we successfully decelerate the cation transport dynamics, allowing the dual-doping process to occur. As a result, Pu2gT OECT exhibits improved current sensitivity compared with the anion-dominated counterpart, showing potential in high-quality electrocardiogram signal acquisition and ion concentration discrimination. Furthermore, incorporating crown ether additives into Pu2gT enhances the dual-doping effect by further delaying cation dynamics, leading to even higher device performance. This dual-doping mechanism deepens the understanding of OECT working principles and opens avenues for achieving state-of-the-art bioelectronic devices.

## Abstract (web summary)

Organic electrochemical transistors (OECT) rely on the coupling between ionic and electronic species. Here, Zhang et al. show that delaying cation expulsion enables an anion-cation dual-doping mechanism in OECTs, thus improving current sensitivity and fidelity for physiological signal acquisition.

## Introduction

Emerging bioelectronics^1,2^ are required to transduce and amplify weak ionic signals in biology into high-amplitude electronic signals in semiconductor devices with exceptional current sensitivity (characterized by high transconductance and low subthreshold slope)^3,4^. Such ion-to-electron transducers have previously been reported using ion bipolar junction transistors^5^, protonic field-effect transistors^6^ and additional transducing interlayers^7^ in solid-contact ion-selective electrodes. However, these devices still lack sufficient current sensitivity to generate detectable and high-quality current output in response to small voltage variations. This limitation can mainly be attributed to their interfacial ionic-electronic coupling mechanisms.

A proven strategy to increase the current sensitivity is to utilize volumetric electrochemical doping of the active channel rather than interfacial charge generation as in field-effect transistors. Such a mechanism is well reflected in organic electrochemical transistors (OECTs)^8–11^, where ions from the electrolyte penetrate and dope the bulk of the organic mixed ionic-electronic conductor (OMIEC) channel. A number of advantages of OECTs have been demonstrated, including low working voltage^12,13^, high stability in aqueous environment^14,15^, biocompatibility^16,17^, and the potential for neuromorphic computing^18,19^. However, current OECT devices still face challenges in detecting ultraweak bioelectric signals, requiring enhanced current sensitivity in ionic-electronic transduction.

The current sensitivity of an OECT is hinged upon its ionic-electronic coupling efficiency, and a number of previous works have been working on improving such an efficiency^20–24^. It is noteworthy that the current understanding of OECTs (take p-type devices as an example) is typically based on a mechanism where ionic-electronic coupling is dominated by anions, and cations have limited, if any, contributions to the electrochemical doping, possibly through a stepwise process^25^; in other words, only one type of ions contribute to the doping process at a given potential. Having recognized this fact, we propose that by regulating ion transport dynamics, an anion-cation dual-doping process, i.e., simultaneous cation-expulsion- and anion-injection-induced electrochemical doping, is possible to occur. Such a dual-doping process could result in a higher charge quantity variation rate dQ / dt, translating to a higher current sensitivity dI / dV under a linearly changed voltage profile. Nevertheless, effective and controllable ion dynamics regulation methods have not been reported yet, impeding the implementation of the dual-doping process to increase the OECT current sensitivity.

In this study, we conceptualize and realize a concurrent cation-expulsion- and anion-injection-induced dual-doping mechanism by designing a p-type OMIEC, poly(3’,4’-bis(2-(2-(2-methoxyethoxy)ethoxy)ethoxy)−2,2’:5’,2”-terthiophene) (Pu2gT). Leveraging coupled voltammetric, gravimetric, spectroscopic, and microstructural characterization under different voltage scan rates, we figure out delayed cation dynamics in Pu2gT as the key prerequisite enabling the dual-doping mechanism. As a result, the Pu2gT OECT exhibits higher current sensitivity, represented by higher transconductance and smaller subthreshold slope compared with the anion-dominated counterpart device. Such improvements can further be enhanced by adding crown ethers into the channel to strengthen the delayed cation expulsion and dual-doping. We also demonstrate that for several OMIECs lacking the dual-doping effect, crown ether additives can be used to introduce cation-additive interactions, thereby converting the anion-dominated doping to dual-doping. Finally, the highly sensitive OECTs are used for electrocardiogram signal acquisition and ion concentration discrimination to demonstrate their potential applications. This dual-doping mechanism provides different perspectives on the operating principles of OECTs, offering valuable guidance for achieving high-performance bioelectronics with exceptional current sensitivity.

## Results

### The dual-doping mechanism and material design

**Fig. 1:**
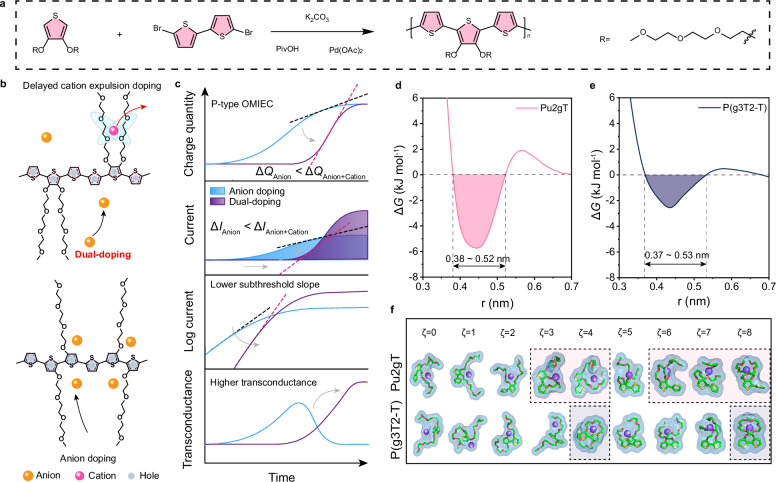
The dual-doping mechanism and material design. **a** Synthetic route of Pu2gT. **b** Schematics of the dual-doping mechanism for Pu2gT (top) and the anion-dominated doping mechanism for P(g3T2-T) (bottom). **c** Schematic illustration of charge quantity, current, subthreshold slope, and transconductance variations in p-type organic mixed ionic-electronic conductor (OMIEC) materials under dual-doping versus anion-dominated doping mechanisms. **d**, **e** The free energy change (ΔG) as a function of the distance r between the center of Na+ cations and the geometric center of the Pu2gT (**c**) and P(g3T2-T) (**d**) oligomers. **f** Illustrations of the Na^+^-oligomer bound states for Pu2gT (top) and P(g3T2-T) (bottom) at different coordination numbers ζ. The stable coordination structures are highlighted by additional shading.

The molecular structure and synthetic route of Pu2gT are shown in Fig. 1a, Supplementary Figs. 1–6. Unlike most polythiophene OMIECs, the oligo(ethylene glycol) (OEG) side chains of Pu2gT are distributed in an axisymmetric and localized manner^26–30^. We hypothesize that the localized OEG side chains can lead to strong cation-side chain chelation^31–33^ similar to the cation-trapping effect observed in crown-ether-functionalized OMIECs^34,35^. Consequently, when a doping voltage is applied, the pre-stored and bound cations are reluctant to be expelled from the Pu2gT film. This delayed cation expulsion process can then coincide with anion injection once a sufficient electrochemical bias is reached, allowing both the cation and anion to contribute to hole generation (Fig. 1b, top). A key feature of this dual-doping mechanism is the significant temporal overlap of cation expulsion and anion injection, which differs from previously reported cationic and anionic processes in OMIECs^25^ and organic radical polymers^36^. For the well-studied OMIEC P(g3T2-T)^37^, which shares a similar molecular structure to Pu2gT except for the centrosymmetric and more distributed side chains, the interactions between cations and side chains are likely to be weaker than Pu2gT. This will result in an anion-dominated doping mechanism (Fig. 1b, bottom).

We further anticipate that the dual-doping mechanism can improve the OECT performance, as the schematics in Fig. 1c illustrate. For p-type OMIECs operating in accumulation mode, passively swollen cations inherently act as de-dopants that suppress film conductivity. In Pu2gT, the strong side chain-cation interactions effectively trap cations within the film at low voltages and inhibit charge generation. Only when the applied voltage exceeds a critical threshold do the confined cations overcome the interactions with side chains under the driving electric field. Concurrently, anions from the electrolyte permeate the film, resulting in the temporal overlap of cation expulsion and anion injection processes. This overlap enables a substantial charge quantity variation (ΔQ) with a small voltage increment, reflected in the delayed yet abrupt charge accumulation profile in the upper panel of Fig. 1c, corresponding to an enhanced dQ/dt. In contrast, single-ion doping is likely to exhibit an earlier onset but a more flattened charge accumulation profile with reduced dQ/dt. Consequently, dual-doped devices are expected to demonstrate a steeper current increase in transfer characteristics compared with anion-dominated devices, which can translate to a higher transconductance (gm) and a reduced subthreshold slope (SS). Therefore, we propose that rational side chain engineering to modulate cation dynamics represents a promising strategy for enhancing OECT current sensitivity.

To investigate the interactions between cations and side chains, we performed well-tempered metadynamics simulations on neutral oligomeric models of both Pu2gT and P(g3T2-T) in the same environment comprising a single Na+ and water^31,38^. Figure 1d, e, Supplementary Fig. 7 show the change in free energy (ΔG) as a function of the geometric distance between the Pu2gT oligomer and the Na+, r. It is found that the cation will chelate with the OEG side chain spontaneously (ΔG < 0) within a wide range of 0.08 < *r* < 0.52 nm. The strength of the interaction is relatively strong, represented by a ΔG minimum of −7.7 kJ mol^−1^ at *r* = 0.18 nm. Such an interaction is around 3 kT, indicating most cations in the Pu2gT film will be bound at room temperature. By contrast, the ranges for ΔG < 0 are narrower for P(g3T2-T) (0.12 < *r* < 0.22 nm and 0.32 < *r* < 0.54 nm), and a less negative ΔG minimum of −3.2 kJ mol^−1^ is reached at *r* = 0.18 nm (Fig. 1d). The interactions for P(g3T2-T) are only around 1kT, indicating only half of the cations in a P(g3T2-T) film are bound at room temperature, and they will be dynamically unbinding and binding due to thermal fluctuations. The higher binding energy for Pu2gT than P(g3T2-T) can be attributed to the fact that the Pu2gT can fully coordinate the Na+ ion without needing any adjustment to the backbone, whereas the P(g3T2-T) backbone needs to twist to achieve full coordination between Na+ and OEG side chains^31^. These results confirm our hypothesis that cations tend to interact with Pu2gT spontaneously and strongly, thanks to its axisymmetric and closely located side chains.

We next simulated ΔG as a function of the coordination number ζ, which quantifies the number of side-chain oxygen atoms that are within 0.38 nm of the Na+ cation. All coordinated structures are illustrated in Fig. 1f, Supplementary Fig. 8, where stable states (i.e., ΔG < 0) are highlighted. Five out of eight possible ζ values correspond to stable cation binding with Pu2gT, whereas only two stable states are observed for P(g3T2-T). These findings reveal that rational side chain engineering enables substantial enhancement of cation chelation efficacy, which constitutes the critical determinant for achieving delayed cation expulsion dynamics. The DFT results and basic material properties of Pu2gT and P(g3T2-T) are summarized in Supplementary Figs. 9–12.

### Evidence for the dual-doping mechanism and microstructure

To validate the delayed cation dynamics and the dual-doping process, we carried out electrochemical quartz crystal microbalance with dissipation monitoring (EQCM-D) and operando UV-vis-NIR absorption spectroscopy measurements. Both techniques were coupled with cyclic voltammetry (CV) at a series of scan rates. We chose 0.1 M NaPF6 aqueous solution as the electrolyte, as large and soft anions like PF6− can enhance the cation-side chain interactions in a water environment^25,32^ and thus magnify the dual-doping effect. Supplementary Figs. 13–18 show the EQCM-D results and absorption spectra of Pu2gT and P(g3T2-T) films, indicating very different ion behaviors and doping mechanisms between the two materials.

**Fig. 2:**
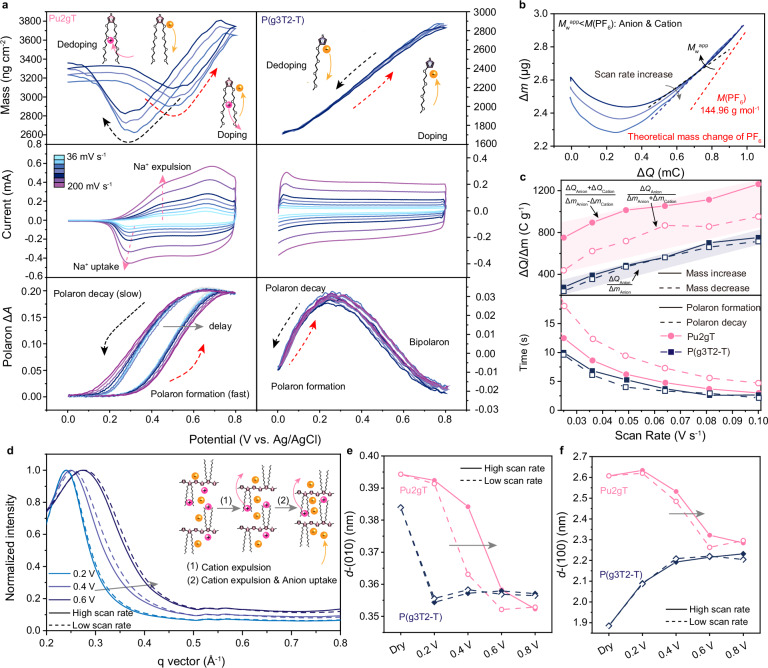
EQCM-D, operando UV-vis-NIR absorption, and GIWAXS results revealing the dual-doping mechanism. **a** EQCM-D-derived mass change (top), cyclic voltammogram (middle), and UV-vis-NIR spectra-derived polaron differential absorbance (bottom) with respect to the doping potential for Pu2gT and P(g3T2-T) films in 0.1 M NaPF6 aqueous electrolyte. Results from different scan rates are shown and compared. The polaron absorbance was extracted at 850 nm for Pu2gT spectra and 900 nm for P(g3T2-T) spectra. **b** The mass change of Pu2gT film versus charge passed through the film in 0.1 M NaPF6 aqueous electrolyte during 0.064 V s^−1^, 0.081 V s^−1^, 0.1 V s^−1^ scans. Linear fittings are applied to extract the apparent molecular weight Mwapp. **c** The charge generated per unit mass change and polaron generation/decay time at different scan rates for Pu2gT and P(g3T2-T) films in 0.1 M NaPF6 aqueous electrolytes. Here, Δm is obtained by calculating the difference between the maximum mass during the forward scan (0 to 0.8 V) and the minimum mass during the reverse scan (0.8 to 0 V). ΔQ is extracted by integrating the current-time (I-t) curve. **d** One-dimensional GIWAXS linecuts of Pu2gT films electrochemically doped at different voltages with different scan rates (high scan rate is 0.1 V s^−1^ and low scan rate is 0.016 V s^−1^). The inset illustrates the different impacts of two doping mechanisms on molecular packing. **e**, **f** Changes in (010) (**e**) and (100) (**f**) spacings of Pu2gT and P(g3T2-T) at different doping voltages and scan rates (high scan rate is 0.1 V s^−1^ and low scan rate is 0.016 V s^−1^).

The EQCM-D-derived mass change, cyclic voltammograms, and optical spectra-derived polaron differential absorbance (ΔA) are plotted against the doping potential in Fig. 2a. Two peaks can be observed in the cyclic voltammogram for Pu2gT in both forward (0 to 0.8 V) and reverse scans. The one at the lower potential can be attributed to Na+ expulsion (uptake) during doping (dedoping) as described in a previous study on doping mechanisms of organic radical polymers^36^. This is corroborated by the concurrent mass loss rate peak and the first oxidation peak (Supplementary Fig. 19), confirming cation involvement in the electrochemical (de)doping of Pu2gT. Furthermore, for the Pu2gT film, the initial mass decrease during the forward scan (i.e., doping) and the final mass increase during the reverse scan (i.e., dedoping) reiterate the cation-related mechanism, and the lower rate for the initial mass decrease than that for the final mass increase suggests a delayed cation expulsion process due to strong Na+-side chain interactions, as expected from Fig. 1d, e. Additionally, the polaron ΔA curve for Pu2gT shows evident hysteresis, again confirming the dissimilar cation transport dynamics between forward and reverse scans. Collectively, these results demonstrate that cation expulsion is hindered in Pu2gT during electrochemical doping, in contrast to P(g3T2-T), where none of these features can be observed (Fig. 2a, right column). Furthermore, the dual-doping process can be modulated by voltage scan rates (Fig. 2a). At lower rates, cation expulsion spanning over a sufficiently long period results in relatively small temporal overlap with anion injection, thereby weakening the dual-doping effect. Conversely, enhanced dual-doping effects can be observed in fast scans due to the pronounced overlap between cation expulsion and anion injection.

To confirm the simultaneous cation expulsion and anion injection, we plot the mass change (Δm) over the charge change (ΔQ) during Pu2gT doping at different scan rates (Fig. 2b), and the apparent molecular weight (Mwapp) of involved ions can be extracted via linear fitting^39,40^:

1 $$\frac{\Delta m}{\Delta Q}=\frac{{M}_{w}^{{app}}}{{n}^{F}}$$

where n is the valence number of ions (here *n* = 1), and F is the Faraday constant. Hence, the physical meaning of Mwapp is the mass change corresponding to unit charge generation. At the high scan rate (0.1 V s^−1^), the Mwapp is first shown to be negative (i.e., Δm < 0 during ΔQ increases), and then gradually rises to 102.27 *g* mol-1, smaller than the molecular weight of PF6− (144.96 g mol^−1^). We note that some non-ideal factors, e.g., a faradic efficiency of <100% and the accompanying water uptake, can lead to an overestimation of Mwapp. Therefore, the smaller Mwapp value than the theoretical must be a direct consequence of simultaneous PF6− uptake and Na+ expulsion, so the effective mass of PF6− is partly offset by Na + . At lower scan rates, the derived Mwapp value increases, indicating that the mass offset effect of Na+ is reduced and the dual-doping is weakened. The analysis of Mwapp during the Pu2gT doping process unambiguously validates the dual-doping process, the degree of which can be regulated by the voltage scan rate. By contrast, the Mwapp for P(g3T2-T) is always higher than the theoretical value of 144.96 g mol^−1^ at all scan rates (Supplementary Fig. 16), as non-ideal factors intervene.

We next analyze charge-per-mass variation (ΔQ/Δm) and polaron formation/decay dynamics during doping/dedoping cycles (0 to 0.8 V) for Pu2gT and P(g3T2-T) across different scan rates (Fig. 2c). As anticipated, Pu2gT generated significantly more charges with smaller mass changes compared to anion-dominated P(g3T2-T), attributed to concurrent cation-expulsion- and anion-injection-induced doping. Notably, Pu2gT exhibited pronounced disparities in ΔQ/Δm ratios and polaron formation/decay dynamics between doping and dedoping stages under identical conditions, a hallmark of the dual-doping process absent in P(g3T2-T). These findings confirm that enhanced side chain chelation of cations promotes temporal overlap of cation expulsion and anion injection, accelerating charge generation. Furthermore, voltage scan rates function as a valuable tool for controlling cation expulsion dynamics and the dual-doping process. Supplementary Fig. 28 shows the annealing-dependent CV curves of Pu2gT, confirming that thermal treatment affects the cation-side-chain interactions and doping dynamics.

In addition, we investigated the morphological evolution during the dual-doping process, and in-situ grazing-incidence wide-angle X-ray scattering (GIWAXS) was employed to monitor structural changes of Pu2gT and P(g3T2-T) at different doping levels (Fig. 2d, Supplementary Figs. 20–25 and Supplementary Table 1). In the pristine state, Pu2gT exhibits a larger lamellar spacing (26.7 Å) and π-π stacking distance (3.94 Å) compared to P(g3T2-T) (18.9 Å and 3.83 Å, respectively), attributed to steric hindrance from its densely attached side chains. The cryo-EM results also demonstrate that Pu2gT has a looser packing structure, which facilitates ion storage (Supplementary Fig. 25). Upon NaPF6 immersion, the molecular packing of P(g3T2-T) expands due to ion penetration, while the microstructure of Pu2gT remains largely unchanged. Under voltage bias, anion injection continuously increases the lamellar spacing in P(g3T2-T) while promoting backbone planarization and reducing the π-π stacking distance, consistent with previous reports^41,42^. In contrast, Pu2gT displays anomalous contraction of lamellar spacing under bias, in agreement with cation expulsion. Notably, Pu2gT exhibits scan-rate-dependent structural dynamics—slow scans induce earlier morphological transitions, while fast scans delay structural changes—consistent with EQCM-D and operando UV-vis-NIR absorption spectroscopy observations in Fig. 2a. This scan-rate-dependent behavior correlates with the temporal overlap of cation expulsion and anion injection: reduced overlap leads to decreased dual-doping efficacy at low rates, whereas rapid scans promote dual-doping by enhancing this overlap. Crucially, all scan rates converge to similar final lamellar and π-π stacking distances, indicating that dual-doping modulates doping dynamics rather than thermodynamic equilibrium. These results unveil the distinct microstructural evolution in the dual-doping process compared to conventional anion-dominated doping.

### Effect of dual-doping and OECT performance

**Fig. 3:**
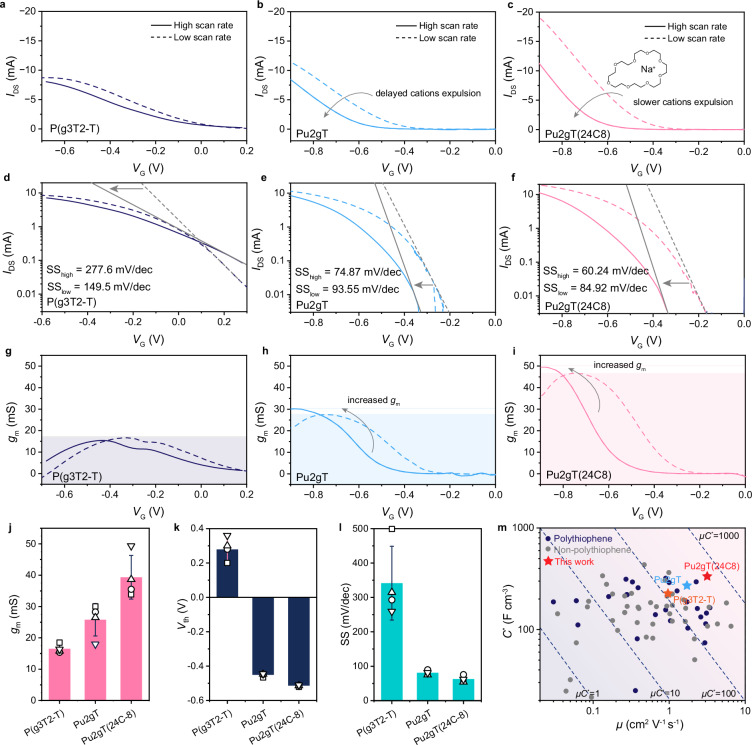
OECT performance. **a**–**c** Transfer characteristics of P(g3T2-T) (**a**), Pu2gT (**b**), and Pu2gT(24C8) (**c**) c-OECTs in 0.1 M aqueous NaPF_6_ electrolyte. The dashed lines and solid lines represent the results obtained at the low scan rate (0.05 V s^−1^) and high scan rate (1 V s^−1^), respectively. The inset of **a** illustrates the c-OECT device structure, and the inset of **c** shows the chemical structure of 24‑Crown‑8 (24C8). **d**–**f** Transfer characteristics of P(g3T2-T) (**d**), Pu2gT (**e**), and Pu2gT(24C8) (**f**) c-OECTs in logarithmic scale in 0.1 M aqueous NaPF_6_ electrolyte. **g**–**i** Transconductance (*g*_m_) curves of P(g3T2-T) (**g**), Pu2gT (**h**), and Pu2gT(24C8) (**i**) c-OECTs in 0.1 M aqueous NaPF_6_ electrolyte. **j**–**l** Statistical comparison of *g*_m_, threshold voltage (*V*_th_), and subthreshold slope (SS) for P(g3T2-T) (**j**), Pu2gT (**k**), and Pu2gT(24C8) (**l**) c-OECTs at a scan rate of 1 V s^−1^. Data are shown as mean ± SD with individual data points overlaid. **m** Summary of *μ* and C* for materials reported in this work and other p-type OMIECs.

Having validated the delayed cation dynamics and dual-doping mechanism of Pu2gT, we fabricated conventional planar organic electrochemical transistors (c-OECTs) to evaluate their impacts on device performance. Supplementary Figs. 26, 27 provide the output characteristics, scan-rate-dependent transfer curves of the devices. As hypothesized (Fig. 1c), for Pu2gT OECTs, both cations and anions contribute to charge generation during electrochemical doping, inducing a rapid increase in source-drain current (IDS). Consequently, Pu2gT OECTs are anticipated to exhibit improved current sensitivity compared to P(g3T2-T) devices. In planar OECTs gated by NaPF6 aqueous electrolytes (Fig. 3a, b), Pu2gT demonstrates a higher threshold voltage (Vth) relative to P(g3T2-T), attributed to its deeper highest occupied molecular orbital (HOMO) level (Supplementary Fig. 11) and delayed charge generation. Notably, the Pu2gT OECT exhibits a pronounced Vth increase at higher scan rates, consistent with enhanced dual-doping effects, whereas the P(g3T2-T) device shows negligible Vth shifts.

To elucidate the role of dual-doping in improving current sensitivity, we compared the SS and gm of both devices at low and high scan rates (Fig. 3d, e, g, h). The results reveal that the Pu2gT OECT achieves a lower SS (SSlow = 93.55 mV/dec) and can be further reduced at high scan rates (SShigh = 74.87 mV/dec), contrasting with the P(g3T2-T) device, which exhibits higher SS values (SSlow = 149.5 mV/dec and SShigh = 277.6 mV/dec). Nonetheless, it is important to note that the enhancement in SS for Pu2gT-based OECTs is not an inherent outcome of dual-doping but depends critically on which mechanism dominates at the onset of conduction. In our system, the faster anion injection kinetics govern switching near the threshold, thereby enabling the steep current rise and reduced SS. Should the slower cation-driven process dominate instead, the current gain would be suppressed, and the SS improvement would likely be lost. Therefore, this SS enhancement is conditional on the predominance of the faster anionic process, a key nuance that delineates the scope of our dual-doping findings. Similarly, the Pu2gT device demonstrates scan-rate-dependent gm enhancement, while the P(g3T2-T) counterpart maintains constant gm. Intriguingly, Pu2gT OECT exhibits higher gm in planar devices compared to P(g3T2-T) under suboptimal charge transport conditions due to Pu2gT’s face-on crystallite orientation (Supplementary Fig. 20), which is disadvantageous for lateral charge transport in planar OECTs. We propose that the higher gm under such unfavorable morphology underscores the intrinsic benefits of Pu2gT’s dual-doping mechanism.

However, quantifying the respective contributions of molecular packing and dual-doping effects to device performance remains challenging. To address this issue, we implemented an additive strategy to modulate dual-doping without altering morphology. Given that cation trapping is a prerequisite for dual-doping, we introduced 24-crown-8 ether (24C8)—a macrocyclic host with eight oxygen atoms—into the Pu2gT matrix. The selection of 24C8 was driven by its moderate binding affinity for small cations (e.g., Na^+^) and its large cavity size, enabling a controlled dual-doping process. GIWAXS analysis (Supplementary Fig. 29, Supplementary Table 2) confirmed that the incorporation of 24C8 does not induce significant morphological changes, corroborating dual-doping as the dominant variable. Subsequent CV and operando UV-vis-NIR absorption spectroscopy (Supplementary Fig. 30) revealed delayed cation expulsion and polaron generation onset in 24C8-modified Pu2gT, alongside accelerated polaron formation and current response at high scan rates. These observations indicate that the addition of 24C8 strengthens cation-polymer interactions and amplifies dual-doping effects while maintaining thin-film morphology and charge transport conditions unchanged. In device tests, the 24C8-containing Pu2gT OECT exhibits larger Vth, reduced SS, and significantly improved gm (Fig. 3c, f, i). Statistical analysis (Fig. 3j–l) quantified these trends: Vth increases from −0.451 ± 0.013 V to −0.514 ± 0.005 V, gm rises from 25.79 ± 5.17 mS to 39.34 ± 6.97 mS, and SS decreases marginally from 81.12 ± 7.73 mV/dec to 62.74 ± 9.25 mV/dec. These metrics collectively demonstrate that the dual-doping mechanism indeed enhances OECT current sensitivity (Supplementary Figs. 31–33). The simultaneous GM enhancement and SS reduction yield a substantially improved μC*, the product of the mobility μ and volumetric capacitance C* benchmarking OMIECs (Supplementary Figs. 34, 35). The μ and C* of Pu2gT(24C8) are determined to be 2.83 ± 0.05 cm^2^ V^−1^ s^−1^ and 337.5 ± 7.3 F cm^−3^, respectively, indicating a μC* product of 955.5 ± 18.75 F cm^−1^ V^−1^ s^−1^, one of the highest reported μC* values among p-type OMIECs (Fig. 3m, Supplementary Tables 3, 4).

Finally, to validate the universality of this strategy, we added the 24C8 additive to other OMIEC systems, including P(gDPP-T) and P(gDPP-TT). Both corresponding OECT devices exhibit enhanced current sensitivity (Supplementary Figs. 36–38), consistent with the additive-induced dual-doping mechanism. This systematic improvement across different OMIECs highlights the broad applicability of our dual-doping strategy, which leverages cation-side chain interactions to enhance the current sensitivity without needing to modify the material’s chemical structure. The consistency in current sensitivity improvements further validates the robustness of this approach, suggesting its potential as a generalizable materials design and device engineering principle for optimizing electrochemical doping dynamics in OECTs.

### Application for dual-doping OECT device

**Fig. 4:**
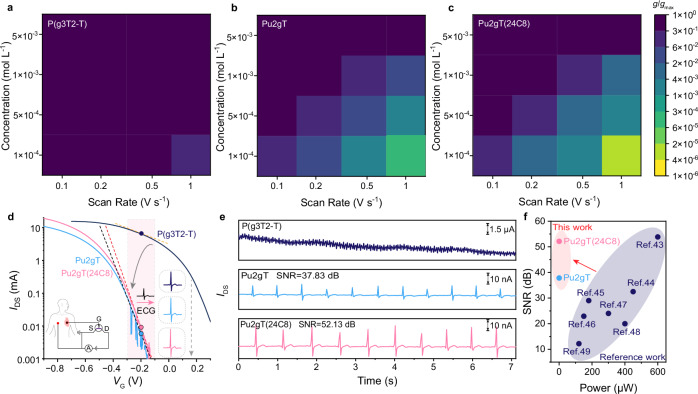
Applications for dual-doped OECTs. **a**–**c** Normalized transconductance (gm/gm, max) of P(g3T2-T) (**a**), Pu2gT (**b**), and Pu2gT(24C8) (**c**) OECTs under varying NaPF6 concentrations and scan rates. **d** Transfer characteristics of P(g3T2-T), Pu2gT, and Pu2gT(24C8) OECTs in the logarithmic scale. The inset illustrates the electrocardiogram (ECG) test circuit and mechanism. **e** Current response and signal-to-noise ratio (SNR) of P(g3T2-T), Pu2gT, and Pu2gT(24C8) OECTs for ECG signal acquisition. **f** Summary of SNR and power for this work and other reported works^43–49^.

Leveraging the unique ion dynamics of the dual-doping mechanism and the resulting device performance enhancement, we demonstrate the feasibility of weak-signal detection, such as ion concentration differentiation and faint electrocardiographic (ECG) signal monitoring. Systematic characterization of transfer curves for P(g3T2-T), Pu2gT, and Pu2gT(24C8) devices under varying scan rates and ion concentrations (Fig. 4a–c) indicates that gm may be used as a parameter for ion concentration differentiation. The P(g3T2-T) device exhibits limited sensitivity, with gm variation confined to about one order of magnitude. In contrast, the dual-doped devices (Pu2gT and its 24C8-modified counterpart) achieve a six-order-of-magnitude gm variation, indicating improved ion concentration discrimination capability. Such high performance can be attributed to delayed cation expulsion and the dedoping effect these cations induce, especially at low ion concentrations.

Furthermore, we demonstrate high-quality ECG signal detection using the dual-doped OECT. As shown in Fig. 4d, the comparison of IDS variations between the three devices indicates that in the subthreshold operating regime, the high-amplitude components of ECG signals are selectively amplified while the low-amplitude noise is scaled down, achieving a filtering effect. Figure 4e, Supplementary Figs. 39–41 display the ECG signals measured by the three devices, where the Pu2gT material demonstrates better signal acquisition quality due to its reduced subthreshold slope. Moreover, with the introduction of the 24C8 additive, the device’s SS further decreases, increasing the signal-to-noise (SNR) ratio from 37.83 dB to 52.13 dB. At the same time, the power consumption of this dual-doped device (6 nW) is lower than that of existing OECT devices when detecting ECG signals, making it one of the optimal devices for ECG monitoring (Fig. 4f).

## Discussion

In summary, this work elucidates an anion-cation dual-doping mechanism in OECTs, overcoming the limitations of the conventional single-ion-dominated or cation-anion stepwise doping process. By developing a novel p-type OMIEC, Pu2gT, with side chains that can strongly interact with cations, we demonstrate that delayed cation expulsion is the key prerequisite for dual-doping, which features concurrent cation expulsion and anion injection and can greatly improve the OECT current sensitivity. Furthermore, the universality of this strategy is confirmed by incorporating crown ether additives to enhance dual-doping effects and further boost the current sensitivity. Compared to traditional anion-dominated OECTs, the dual-doped Pu2gT OECT exhibits higher gm and reduced SS, enabling precise ion concentration discrimination and high-fidelity ECG signal acquisition. This study not only provides fundamental insights into OECT doping dynamics but also establishes innovative materials design and device engineering strategies for high-performance bioelectronic devices, propelling advancements in next-generation biosensing and health monitoring technologies.

## Methods

### Materials characterization

All chemicals were purchased from Sigma Aldrich and used as received. 1H and 13 C NMR spectra were acquired on a Bruker AVANCE III HD 600 MHz spectrometer. Polymer molecular weights were determined by high‑temperature gel permeation chromatography (GPC) on a Polymer Laboratories GPC‑PL220 system (Agilent Technologies) at 150 °C using trichlorobenzene as the eluent and polystyrene standards.

### Well‑tempered metadynamics simulations

Well‑tempered metadynamics simulations were performed to study the interactions between Na+ cations and glycolated polymer units. Forcefield parameters and topologies for Pu2gT were added to a previously reported forcefield (publicly available on GitHub). Dihedral potentials for the thiophene bearing glycol side chains and its neighboring thiophene without side chains were calculated using DFT at the B3LYP/6‑311 G + (d,p) level. Atomic partial charges were derived using the CHELPG electrostatic mapping at the same level of theory. Water was described with the optimized‑point‑charge model. Metadynamics simulations were carried out using GROMACS 2021.2 and PLUMED 2.4.1. Electrostatics and van der Waals forces were computed with the particle mesh Ewald summation; hydrogen bonds were constrained with the LINCS algorithm (2 fs time step). Energy minimization employed the steepest descent algorithm. Temperature and pressure were controlled using a velocity‑rescale thermostat and a Berendsen barostat, respectively.

A section of Pu2gT or P(g3T2‑T) polymer chain (four thiophene rings) was placed in a 7 × 7 × 7 nm³ box with ≈10000 water molecules. An energy penalty prevented the chloride counter‑ion from approaching within 3 nm of the cation or oligomer. After energy minimization, NVT (1 ns) and NPT (1 ns) equilibration, a 5 ns metadynamics run with 8 walkers was performed. Two collective variables were biased: the distance d between the Na+ and the polymer geometric center, and a continuous coordination‑like variable ψ (defined in the Supplementary Information). Gaussians of height 0.3 kJ mol^−1^ were deposited every 200 ps with a bias factor of 5. Convergence was monitored by the stability of the free‑energy surfaces.

Free energies as a function of the cation‑backbone distance r and the coordination number ζ (number of oxygen atoms within 0.38 nm of Na+) were obtained by reweighting the metadynamics trajectories and applying a weighted histogram analysis. Only frames with *r* < 0.75 nm were considered to ensure the cation remained near a polymer unit. The standard chelation free energy ΔG* was calculated by Boltzmann inversion after adjusting probabilities to a 1 M standard state.

### Grazing‑incidence wide‑angle X‑ray scattering (GIWAXS)

GIWAXS measurements were performed at beamline 7.3.3 of the Advanced Light Source using a 10 keV X‑ray beam and a Dectris Pilatus 2 M detector. Polymer films were spin‑coated onto gold‑coated silicon substrates. Dry films were measured as prepared; wet films were immersed in 0.1 M NaPF_6_ aqueous solution for 10 min and dried under N2. Doped films were produced by cyclic voltammetry (0–0.8 V at 0.016 V s^−1^ or 0.1 V s^−1^) in 0.1 M NaPF6 using a three‑electrode setup (working electrode: polymer film on Au, counter: Pt, reference: Ag/AgCl). After reaching the target potential, samples were removed, dried, and measured. The incidence angle was optimized between 0.11° and 0.15°. Data reduction was performed with the Nika package in Igor Pro.

### Cryogenic electron microscopy (cryo‑EM)

Cryo‑EM samples were prepared by spin‑coating Pu2gT or P(g3T2‑T) from chloroform solution onto PEDOT:PSS‑coated glass substrates, followed by a water‑transfer step to deposit the polymer film onto a glow‑discharged porous carbon film on a copper grid (Quantifoil R 2/2). Grids were manually blotted, plunge‑frozen in liquid ethane, and stored under liquid nitrogen. Imaging was performed on a FEI Talos F200C transmission electron microscope operating at 200 kV in low‑dose mode at −178 °C, using a Gatan 626 cryo‑holder and a FEI CETA 4k × 4k CMOS camera. Magnifications ranged from 92 k to 120 k.

### Density functional theory (DFT) calculations

DFT calculations were performed with Gaussian16 Rev. A.03. Geometry optimizations, frontier molecular orbitals, torsional potentials, and dihedral angle distributions were calculated at the ω‑tuned ωB97XD/6‑31 G(d) level using methoxy groups as surrogates for the full glycol side chains.

### Cyclic voltammetry (CV)

CV measurements were performed on an Autolab PGSTAT302N workstation with a three‑electrode configuration (Ag/AgCl reference, Pt counter, polymer‑coated ITO working electrode). The electrolyte was a 0.1 M NaPF6 aqueous solution or 0.1 M tetrabutylammonium hexafluorophosphate in acetonitrile. The voltage was scanned from 0 to 0.8 V.

### Electrochemical quartz crystal microbalance with dissipation (EQCM‑D)

EQCM‑D measurements were carried out on a Biolin QSense Explorer using gold‑coated quartz crystals (0.785 cm^2^). Polymer films were spin‑coated from chloroform solution and annealed at 70 °C for 1 h. The chip served as the working electrode, with Ag/AgCl reference and Pt counter, connected to an Autolab PGSTAT302N. After injecting 0.1 M NaPF6 electrolyte at 100 μL min^−1^, the system stabilized until the frequency drift was <5 Hz min^−1^. Data were collected during CV scans (0–0.8 V) at rates of 0.016–0.3 V s^−1^. Mass changes were extracted using the Dfind Broadfit model (Qsense Dfind software).

### Operando UV-vis-NIR absorption spectroscopy

Operando UV-vis-NIR spectra were acquired with a Shimadzu UV‑3600 plus spectrometer coupled to an Autolab PGSTAT302N workstation using the same three‑electrode setup and potential scan conditions as for EQCM‑D. Transmittance data were converted to absorbance A = −lg *T*, and differential absorbance ΔA = A– A0. Polaron and π–π* absorbances were monitored at 550 nm and 850 nm for Pu2gT‑based samples, and at 600 nm and 900 nm for P(g3T2‑T).

### Electrochemical impedance spectroscopy (EIS)

EIS was performed on an Autolab PGSTAT302N with the same three‑electrode configuration but using an Au‑coated glass working electrode. A 50 mV sinusoidal AC voltage (105–10^−1^ Hz) was superimposed on a DC bias of 0-0.9 V in 0.1 M NaPF6. Impedance data were analysed with NOVA 2.1 software.

### OECT fabrication and characterization

Conventional planar organic electrochemical transistors (c‑OECTs) were fabricated on cleaned glass substrates with 3 nm Cr and 50 nm Au source/drain electrodes (channel width W = 1000 μm, length L = 50 μm) deposited by thermal evaporation. Polymer solutions (15 mg mL^−1^ in CHCl_3_) were spin‑coated at 2000rpm, giving a channel thickness of d ≈ 80 nm. All devices were measured in 0.1 M NaPF₆ aqueous electrolyte with an Ag/AgCl gate electrode. A Keithley 2602B sourcemeter controlled by Kickstart software was used. Transfer characteristics were recorded at a drain voltage VD = −0.6 V.

## Supplementary information

Supplementary Information

Transparent Peer Review file

## Data Availability

The source data generated in this study have been deposited in the SciDB database under the following DOI: 10.57760/sciencedb.31783. The data are publicly available without restrictions. All other data supporting the findings of this study are available within the article and its Supplementary Information.
