# Supplementary material for: Delayed cation dynamics enables dual-doped organic electrochemical transistors with high current sensitivity
Source: Nat Commun. 2026 Jun 1;17:7029. doi: 10.1038/s41467-026-73762-1 (PMC13392116; doi:10.1038/s41467-026-73762-1)
Supplement: Supplementary file 1 — Supplementary Information [file 41467_2026_73762_MOESM1_ESM.pdf]

Supplementary information for

Delayed Cation Dynamics Enables Dual-Doped Organic Electrochemical Transistors with High Current Sensitivity

Sen Zhang[1#], Bingjun Wang[1#], Nicholas Siemons[2#], Iona Anderson[2], Shijie Wang[1], Yuxin Kong[3], Jin-Ting Ye[4], Xian-Kai Chen[4], Minning Wang[1], Xiao Yu[1], Chi-Yuan Yang[5], Yuxiang Li[3*], Simone Fabiano[5*], Jenny Nelson[2*], and Wei Ma[1*]

[1] State Key Laboratory for Mechanical Behavior of Materials, Xi'an Jiaotong University, Xi'an 710049, China

[2] Department of Physics, Imperial College London, London SW7 2AZ, UK

[3] School of Materials Science and Engineering, Xi'an University of Science and Technology, Xi'an 710054, China

[4] Institute of Functional Nano and Soft Materials (FUNSOM), Joint International Research Laboratory of Carbon-Based Functional Materials and Devices, Soochow University, Suzhou 215123, China

[5]Laboratory of Organic Electronics, Department of Science and Technology, Linköping University, Norrköping, Sweden

[#]These authors contributed equally.

[*]Corresponding authors: liyuxianggj687@xust.edu.cn (Y.L.), simone.fabiano@liu.se (S.F.) jenny.nelson@imperial.ac.uk (J.N.), msewma@xjtu.edu.cn (W.M.)

This file includes:

General Methods

Synthetic Procedures

Supplementary Figures 1–41

Supplementary Tables 1-4

References

General Methods

Materials characterization

All chemicals were purchased from Sigma Aldrich and used as received. ^{1}H and ^{13}C NMR spectra were acquired on the Bruker AVANCE III HD 600 MHz. Polymer molecular weights were characterized on Polymer Laboratories GPC-PL220 high temperature GPC/SEC system (Agilent Technologies) at 150 ºC vs polystyrene standards using trichlorobenzene as the eluent.

Well-tempered metadynamics simulations

Well-tempered metadynamics simulations[1] were performed to study the interactions between cations and glycolated polymer units. The forcefield used was similar to that used in previous work[2] and is publicly available on GitHub[3]. In this study, parameters and topologies for Pu2gT were added to this forcefield. These include the dihedral potential associated with the thiophene bearing the glycol side chains and its neighbouring thiophene without any side chains, as well as the atomic partial charges for a Pu2gT polymer. In the case of the dihedral, a potential was calculated using DFT with a B3LYP functional and a 6-311G+(d,p) basis set. The MD potentials were fitted to the difference in the DFT potential and the background potential in MD. Partial charges were calculated using the same level of theory, and CHELPG electrostatic mapping[4]. Water was modelled using the optimized-point-charge model[5].

Metadynamics simulations were performed using GROMACS 2021.2[6] and PLUMED 2.4.1[7]. Electrostatics and van-der-Waals forces are computed using scalable Particle Mesh Ewald summation. Hydrogen bonds are constrained using a LINCS algorithm, allowing for a 2 fs time step to be used. Energy minimization (EM) steps were carried out using the STEEP algorithm, until the change in energy was less than the numerical accuracy. Temperature coupling was achieved via a velocity-rescale algorithm[8] and a Berendsen barostat[9].

For the simulations we placed either a section of a Pu2gT polymer chain or a section of a P(g3T2-T) polymer chain in a 7×7×7 nm^3 box alongside approximately 10,000 water molecules. Both the P(g3T2-T) and Pu2gT chain sections comprised four thiophene rings to keep the phase space to a computationally accessible size. Secondly, an energy penalty was applied to the chloride ion to stop it coming within 3nm of either the cation or the oligomer. By including the anion, charge neutrality is ensured, and by applying this energy penalty the phase space can be more effectively explored. These simulation boxes were energy minimized, simulated in

NVT for 1 ns and then in NPT for 1 ns. Following this, a 5 ns metadynamics simulation was performed using 8 walkers on each system. The structures obtained from the end of this metadynamics run are used as the starting structures for the final metadynamics production run to ensure the initial states are in different parts of the phase space.

During the metadynamics runs, two collective variables (CVs) are biased: d and ψ. The first, d, measures the distance between the centre of the Na^+ cation and the geometric centre of the polymer section. The second, ψ, is given by

$$\psi = \sum_i \left(\frac{1-\theta_i^6}{1-\theta_i^{12}}\right) \quad (1)$$

where i is an oxygen atom on the polymer side chains, and $\theta_i = r_i/0.5$ and r_i is the distance between atom i and the cation. Therefore, ψ is a rough approximation at a coordination number between the cation and the oxygen atoms, but is continuous and “broad”, ensuring the sampling is enhanced during the simulation to maximum effect.

To make use of parallelization in these calculations we performed multiple-walker simulations using 8 walkers. Using 8 walkers, each at a simulation speed of 130 ns per day, allowed for an effective simulation speed of around 1 μs per day. During the production run, we use hills with a maximum height of 0.3 kJ mol^{-1}, a bias factor of 5, hill widths of 0.05, and 1 in d and ψ, respectively. We deposit a hill every 200 ps during the metadynamics simulation. Convergence is assured through monitoring the free-energy surfaces in d and ψ and continuing the simulation until no further significant changes are observed.

To obtain the free energy in terms of r (the distance from the cation to the backbone near where the side chains are attached) and the coordination number ζ (the number of oxygen atoms on the OEG side chains that are within 0.38 nm of the cation), the corresponding statistical weight for each frame in the trajectory was used to perform a weighted histogram analysis[10]. By Boltzmann-inverted the calculated populations in terms of r and ζ, the free energy in those terms is obtained. In both cases during the reweighting, frames of the metadynamics trajectories were considered only if $r < 0.75$ nm, as we considered larger distances to be unrepresentative of a cation in the bulk of a film where it will be surrounded by polymers in all directions. Therefore, the free energies and standard free energies reported here are conditional; the free energy *given* the cation is within

0.75 nm of a polymeric unit. The choice of a cutoff at $r = 0.75$ nm was chosen as beyond this value, the free energy profile of the simulations begins to follow a $1/r^2$ relationship, indicating that at values above 0.75 nm the cation starts to behave like a free cation in water, rather than a cation interacting with an oligomer.

To calculate the standard free energy of chelation, ΔG^*, the free energy of each simulation was first reweighted in terms of r. Then, three areas of the free energy surfaces were identified. The first, R_B, is where $r < 0.5$ nm and the region where the cation is considered bound to the oligomer. This cutoff was chosen as it is around where the free energy goes from positive to negative, indicating an attractive interaction between the oligomer and the cation. The second, R_U, is where 0.5 nm $< r <$ 0.75 nm and the area where the cation is not bound to the oligomer. The third was where $r >$ 0.75 nm, and those frames were removed from the analysis for the same reasons as explained above. For each simulation, the probability of the cation being bound, p_B, and unbound, p_U, was calculated via reweighting the metadynamics trajectories into a discrete space defined by R_B or R_U. When p_B and p_U are obtained, they are adjusted to find the probabilities p_B^* and p_U^* given a 1M solution;

$$p_B^* = \frac{4\pi\, p_B}{3}(0.5^3)/V_{\text{mol}} \quad (2)$$

$$p_U^* = \frac{4\pi\, p_U}{3}(0.75^3 - 0.5^3)/V_{\text{mol}} \quad (3)$$

where $V_{\text{mol}} = 1.66$ nm^3 and is the volume corresponding to the inverse of 1M concentration. The final standard chelation free energy was then calculated using the Boltzmann inversion, $\Delta G^* = -k\text{T}\, log\left(\frac{p^*_B}{p^*_U}\right)$.

Grazing-incidence wide-angle X-ray Scattering (GIWAXS)

GIWAXS measurements were performed at beamline 7.3.3[11] at the Advanced Light Source. The samples were spin-coated onto gold-coated silicon substrates. For dry film samples, no additional processing steps were required. Regarding wet film samples, those prepared on gold-coated silicon substrates were immersed in 0.1 M sodium hexafluorophosphate ($NaPF_6$) solution for 10 minutes to achieve sufficient swelling, followed by surface drying with nitrogen gas to eliminate residual solution. For doped samples, electrochemical pretreatment was conducted using an Autolab PGSTAT302N workstation configured with a three-electrode system: the film-coated substrate served as the working electrode, platinum as the counter electrode, and

Ag/AgCl as the reference electrode, immersed in 0.1 M NaPF6 electrolyte. The samples were subjected to cyclic voltammetric scanning across distinct voltage windows (0-0.2 V, 0-0.4 V, 0-0.6 V, 0-0.8 V) at sweep rates of 0.016 V s^{-1} and 0.1 V s^{-1}. Upon reaching predetermined doping potentials, the samples were retrieved and dried under nitrogen to remove surface residues. During characterization, a 10 keV X-ray beam was incident on the samples at angles ranging from 0.11° to 0.15° for measurement.Then an optimal angle was selected to maximize the scattering intensity. The scattered X-ray photons were detected using a two-dimensional Dectris Pilatus 2M detector. Data analysis was completed using Igor Pro software with the Nika package[12].

Cryogenic electron microscopy (cryo-EM)

Cryo-EM samples were prepared as follows. PEDOT:PSS (Heraeus Clevios™ PH1000) was spin-coated on glass substrates, on which Pu2gT and P(g3T2-T) chloroform solutions were spin-coated. Next, a water transfer process was applied, and the polymer film was deposited on a copper grid coated with a glow-discharged porous carbon film (Quantifoil R 2/2, Electron Microscopy Sciences). The grids were manually blotted and dried, and then plunge-frozen in liquid ethane that had been cooled by liquid nitrogen. After flash-freezing, the grids were stored in liquid nitrogen. A FEI Talos F200C transmission electron microscopy, running in low dose mode at −178 °C and at 200 kV high tension, was used to characterize cryo-EM samples, which were held on a Gatan 626 cryo-holder. Using a high-sensitivity 4k × 4k pixel FEI CETA CMOS camera, the micrographs were captured at magnifications ranging from 92k to 120k.

Density functional theory (DFT) calculations

DFT calculations were performed on Gaussian 16 Rev. A.03 software. The optimized molecular geometry, spatial distributions of frontier molecular orbitals, torsional potential profiles, and the dihedral angle distribution of Pu2gT and P(g3T2-T) were calculated at the ω-tuned ωB97XD/6-31G(d) level. Note that methoxy groups were used instead of glycolated side chains to simplify the calculation.

Cyclic voltammetry (CV)

Electrochemical analysis was performed on an Autolab PGSTAT302N electrochemical workstation with a conventional three-electrode configuration. The reference, counter, and working electrodes were an Ag/AgCl

wire, a platinum sheet, and a polymer-coated ITO substrate, respectively. The working electrode was fabricated by spin-coating Pu2gT , Pu2gT(24C8) and P(g3T2-T) chloroform solutions onto pre-cleaned ITO substrates. The electrolyte was either 0.1 M $NaPF_6$ aqueous solution or 0.1 M tetrabutylammonium hexafluorophosphate acetonitrile solution. The voltage scan range was 0 to 0.8 V. Ionization potentials (IPs) were determined using the following equation: IP = $q(E_{ox} - E_{Fc} + 4.8)$, where E_{ox} is the oxidation onset potential of the polymer, E_{Fc} is the oxidation onset potential of ferrocene, and q is the elementary charge.

Electrochemical quartz crystal microbalance with dissipation (EQCM-D)

Gold-plated quartz crystal chips (0.785 cm^2) were used in EQCM-D measurements on a Biolin Qsense Explorer instrument. Samples were prepared by spin-coating Pu2gT and P(g3T2-T) chloroform solutions on the chip, and then mildly thermal-annealing the samples at 70 °C in an oven for 1 h to remove any solvent residue. The polymer-coated chips were then installed onto the instrument as the working electrode, and Ag/AgCl and platinum were used as the reference and counter electrodes, respectively. All three electrodes were connected to an Autolab PGSTAT302N workstation.

The Qsoft401 software was used to control the measurement. First, the frequency and dissipation baseline at 1st, 3rd, 5th, 7th, and 9th overtones were recorded, and then the electrolyte (0.1 M $NaPF_6$ aqueous solution) was injected at a speed of 100 μl min^{-1}. The setup was allowed to stabilize until a frequency drift rate of <5 Hz min^{-1} was achieved. The frequency and dissipation data were collected during electrochemical scans between 0 to 0.8 V at a series of rates (0.016, 0.025, 0.036, 0.049, 0.064, 0.081, 0.1, 0.15, 0.2, 0.25, and 0.3 V s^{-1}). Finally, the Qsense Dfind software was used to process the data, and the Dfind Broadfit model was applied to extract the mass change.

Operando UV–vis–NIR absorption spectroscopy

An Autolab PGSTAT302N workstation and a Shimadzu UV-3600 plus spectrometer were connected to carry out the operando UV–vis–NIR absorption spectroscopy. The three electrodes were the same as for CV measurements, and the voltage scan range and rate were the same as for EQCM-D tests. The measured data, transmission T, were converted to absorbance A by $A = -\lg T$, and the differential absorbance ΔA was calculated by subtracting the initial absorbance A_0 from A. The signal corresponding to π–π* and polaron

absorption of Pu2gT and Pu2gT(24C8) was extracted at 550 and 850 nm, respectively, and for P(g3T2-T) the wavelength was 600 and 900 nm.

Electrochemical impedance spectroscopy (EIS)

EIS measurements were performed using an Autolab PGSTAT302N workstation. The three electrodes were the same as for CV measurements, except that the substrate on which the polymer film was deposited was changed to Au-coated glass. The electrolyte was 0.1 M $NaPF_6$ aqueous solution. A small sinusoidal AC voltage of 50 mV amplitude with varying frequency (10^5 to 10^{-1} Hz) was applied to the sample, superimposed on a DC bias potential of 0-0.9 V. The measured frequency-dependent impedance Z was analyzed using NOVA 2.1 software, and the simplified Randles circuit model was chosen, consisting of a series resistor R_S, a parallel resistor R_P, and a parallel capacitor C. The C^* values were calculated by dividing the fitted C by the film volume.

OECT fabrication and characterization

For conventional planar c-OECTs, glass substrates were cleaned in ultrasonic baths with acetone and isopropanol (99.5%, Sigma-Aldrich), respectively, for 30 min each, followed by UV-ozone treatment for 15 min. The substrates were then transferred to a thermal evaporator where 3 nm Cr (0.2 Å s^{-1}) and 50 nm Au (1 Å s^{-1}) were sequentially deposited. The evaporation shadow masks defined the channel width W and length L to be 1000 and 50 μm, respectively. Pu2gT ,Pu2gT(add 3 wt% 24C8), P(g3T2-T) and P(g3T2-T) (3 wt% 24C8) solutions (15 mg ml^{-1}) were spin-coated in the glovebox at a spin speed of 2000 rpm, leading to a channel thickness $d \approx 80$ nm. For other materials, P(gDPP-2T) and P(gDPP-2T) (3 wt% 24C8) solutions (12 mg ml^{-1}) were spin-coated at a spin speed of 3000 rpm, P(gDPP-TT) and P(gDPP-TT) (3 wt% 24C8) solutions (12 mg ml^{-1}) were spin-coated at a spin speed of 1500 rpm.

For OECT characterization, 0.1 M $NaPF_6$ aqueous solution was chosen as the electrolyte and an Ag/AgCl pellet (Wuhan Brain Link Technology Co., Ltd) as the gate electrode. Three electrodes of each device were connected to a Keithley 2602B sourcemeter via an M4 probe station (Shenzhen Uotest Electronic Technology Co., Ltd). Controlled by Kickstart software, the applied drain and gate voltage profiles were programmed as

needed, and the corresponding drain and gate currents were recorded. For transfer curve measurements, the drain voltage $V_D = -0.6$ V.

Synthetic Procedures

All chemicals were purchased from Sigma Aldrich and used as received. P(g3T2-T) was synthesized following the procedure in literature[13]. The synthetic route of Pu2gT is shown in **Figure S1**.

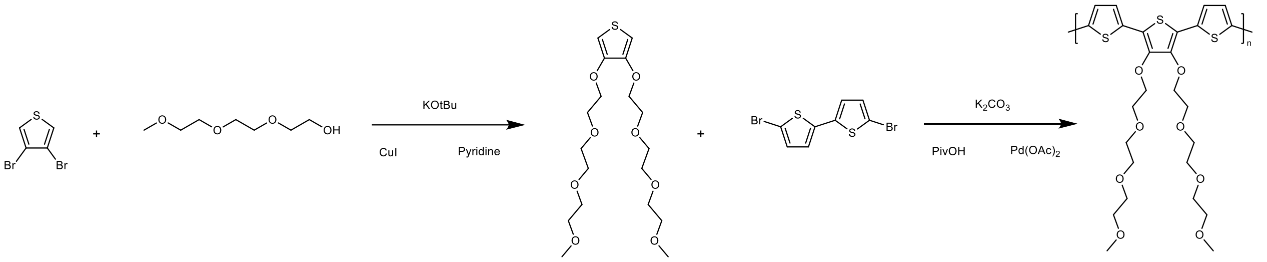

Supplementary Figure 1. Synthetic route of Pu2gT. (i) KOtBu, CuI, pyridine, 115°C to room temperature, 24 h. (ii) $Pd(OAc)_2$, PivOH, K_2CO_3, 90°C, 0.5 h. The monomer 3,4-bis(triethyleneglycolmonomethylether)thiophene was obtained in 38% yield, and the final polymer Pu2gT was obtained in 59% yield after Soxhlet extraction.

3,4-Bis(triethyleneglycolmonomethylether)thiophene

The monomer synthesis was based on a previous report[14]. CuI (0.943 g, 4.95 mmol) and KOtBu (4.17 g, 37.2 mmol) were added to a dried 100 ml two-neck round-bottom flask and purged under N_2 for 15 min, after which toluene (50 ml), pyridine (5 ml) and triethylene glycol monomethyl ether (6 g, 36.54 mmol) were added in order. The resulting brown suspension was stirred under N_2 at room temperature for 1 h, after which 3,4-dibromothiophene was added (3 g, 12.4 mmol). The reaction was then heated to reflux in the dark under N_2 for 24 h. After cooling to room temperature, the black liquid supernatant was gathered by decanting, and concentrated using rotary evaporation to a volume of less than 5 ml. This black residue was diluted using dichloromethane (200 ml) and washed with HCl (5 M, 2 × 200 ml). After gathering and drying the organic fraction with $MgSO_4$, the solvent was extracted using rotary evaporation, yielding a black-brown residue. This was purified by silica gel column chromatography eluting 1:1 hexane/EtOAc followed by EtOAc to achieve the title compound as a yellow oil (1.93 g, 38% yield). ^1H NMR (600 MHz, $CDCl_3$): δ (in ppm) = 6.21 (s, 2H), 4.12 (t, J = 5.1 Hz, 4H), 3.83 (t, J = 5.1 Hz, 4H), 3.70 (dd, J = 6.0, 3.7 Hz, 4H), 3.65–3.60 (m, 8H), 3.55–3.51 (m, 4H), 3.36 (s, 6H). ^{13}C NMR (151 MHz, $CDCl_3$): δ (in ppm) = 147.08, 97.87, 71.92, 70.78, 70.64, 70.54, 69.81, 69.49, 59.03.

Pu2gT

3,4-bis(triethyleneglycolmonomethylether)thiophene (122.5 mg, 0.3 mmol) and 5,5'-dibromo-2,2'-bithiophene (97.2 mg, 0.3 mmol) were dissolved in dry DMAc (10 ml), and the light yellow solution was

deoxygenated by N_2 bubbling for 30 min. K_2CO_3 (107.8 mg, 0.78 mmol), pivalic acid (9.2 mg, 0.09 mmol), and $Pd(OAc)_2$ (2.0 mg, 0.009 mmol) were added to a dry reaction tube (25 ml), which was sealed and purged under N_2 for 20 min. After deoxygenation, the DMAc solution was transferred into the sealed reaction tube vial via a syringe, and the reaction was heated to 90°C in a covered oil bath. After 0.5 h of heating, a dark red viscous liquid was achieved, and the reaction was stopped by cooling to room temperature. This viscous liquid was precipitated into MeOH (200 ml), and the resulting solid was filtered into a Soxhlet thimble and washed by Soxhlet extraction with MeOH, hexane, acetone, and EtOAc in order. The desired product was then recovered by Soxhlet extraction with $CHCl_3$. After that, the chloroform solution is subjected to rotary evaporation to form a layer of golden flake polymer on the bottle wall. MeOH was added to the bottle for ultrasonic treatment to peel off the flakes, and the product was obtained by filtration. Finally, the polymer was dried under a high vacuum before being stored under N_2 atmosphere (102 mg, 59.3% yield). ^{1}H NMR (600 MHz, $CDCl_3$): δ (in ppm) = 7.23 (br s), 7.11 (br s), 4.35 (br s), 3.87 (br s), 3.76–3.58 (br m), 3.53 (br m), 3.36 (br s).

For materials characterization, ^{1}H and ^{13}C NMR spectra were acquired on a Bruker AVANCE III HD 600 MHz instrument. Molecular weights were characterized on Polymer Laboratories GPC-PL220 high-temperature GPC/SEC system (Agilent Technologies) at 150°C, using polystyrene as the standard and trichlorobenzene as the eluent.

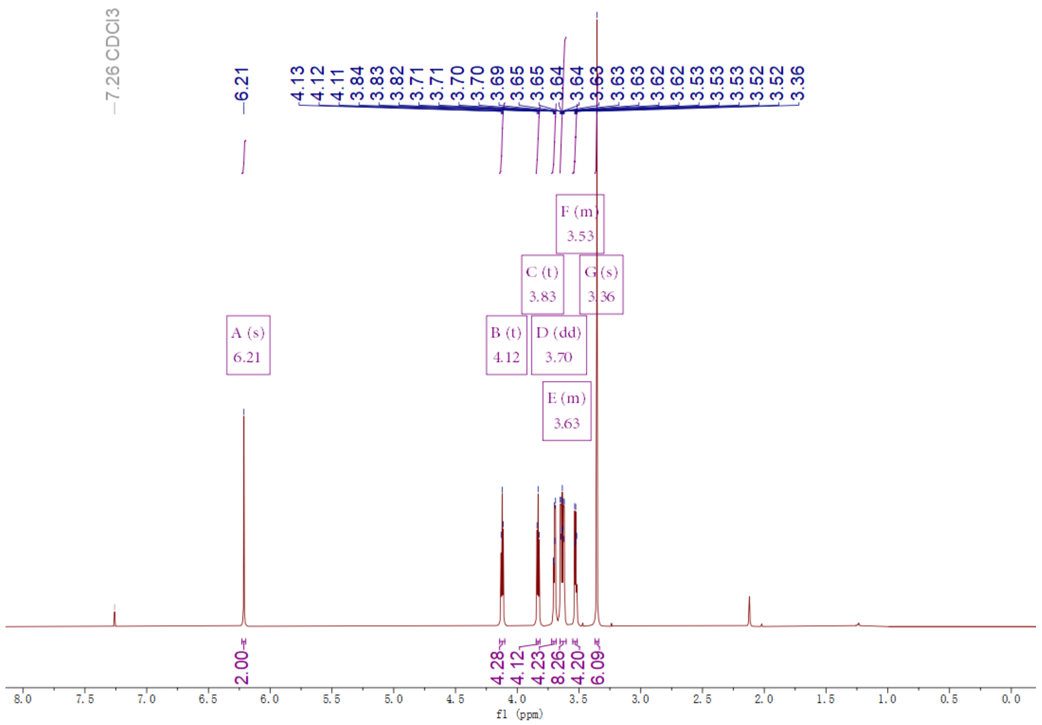

Supplementary Figure 2. ^{1}H NMR of 5,5'-dibromo-4,4'-bis(2-methoxyethoxy)-2,2'-bithiophene in $CDCl_3$. The figure shows the ^{1}H NMR spectrum of compound in $CDCl_3$, confirming its structure.

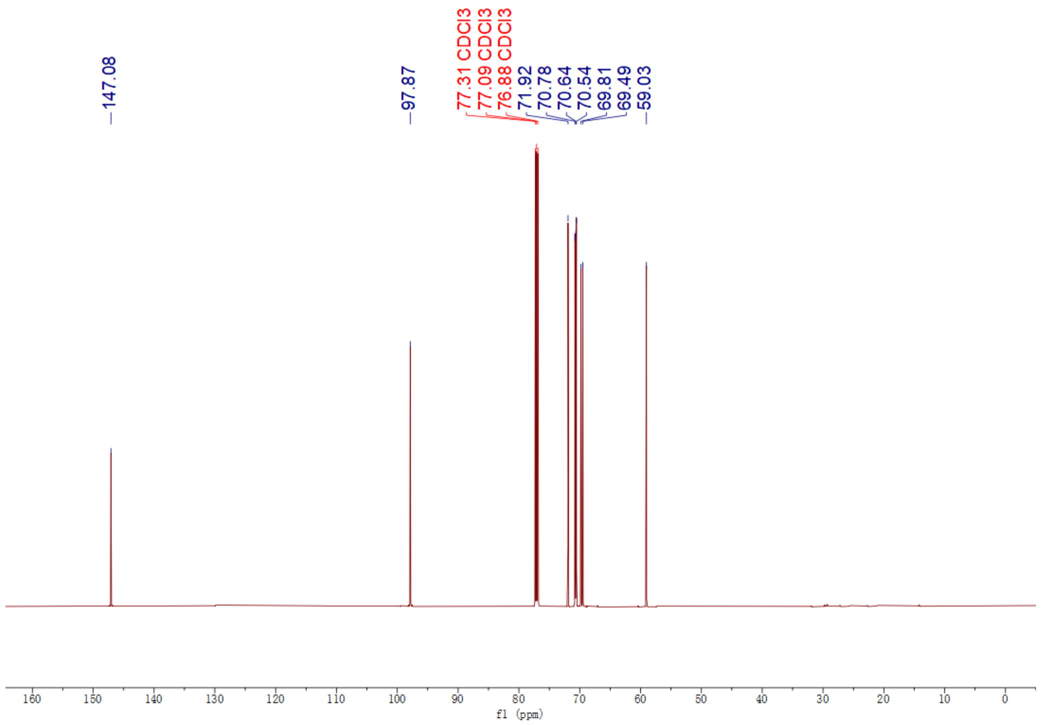

Supplementary Figure 3. ^{13}C NMR of 5,5'-dibromo-4,4'-bis(2-methoxyethoxy)-2,2'-bithiophene in $CDCl_3$. The figure shows the ^{13}C NMR spectrum of compound in $CDCl_3$, confirming its structure.

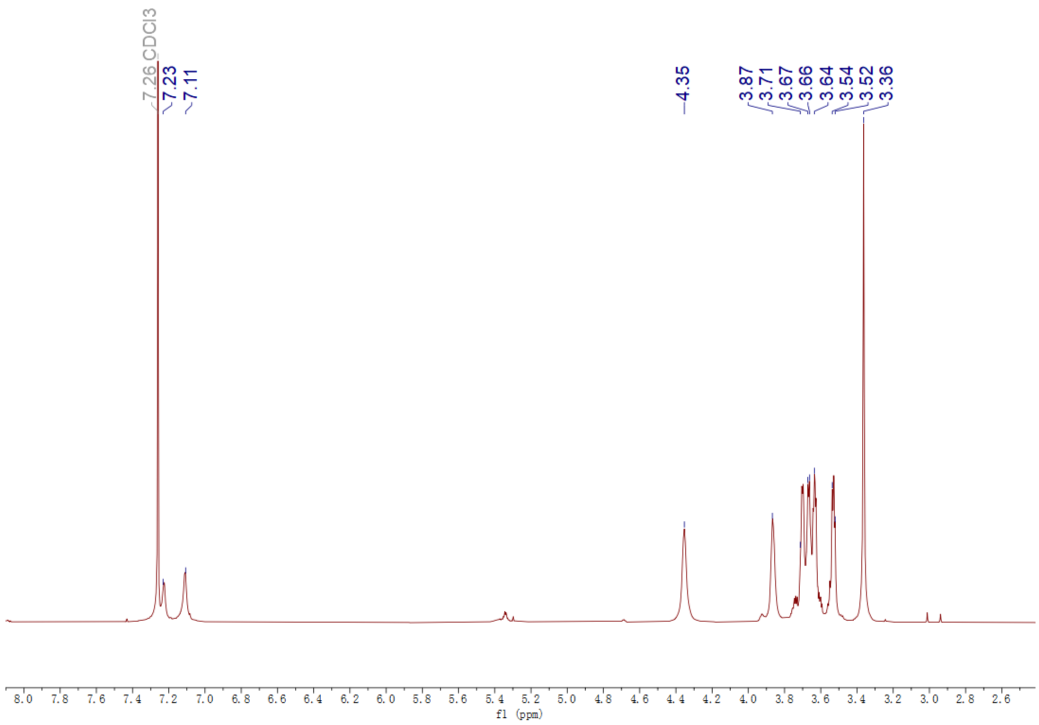

Supplementary Figure 4. ^{1}H NMR of Pu2gT. The figure shows the ^{1}H NMR spectrum of compound in $CDCl_3$, confirming its structure.

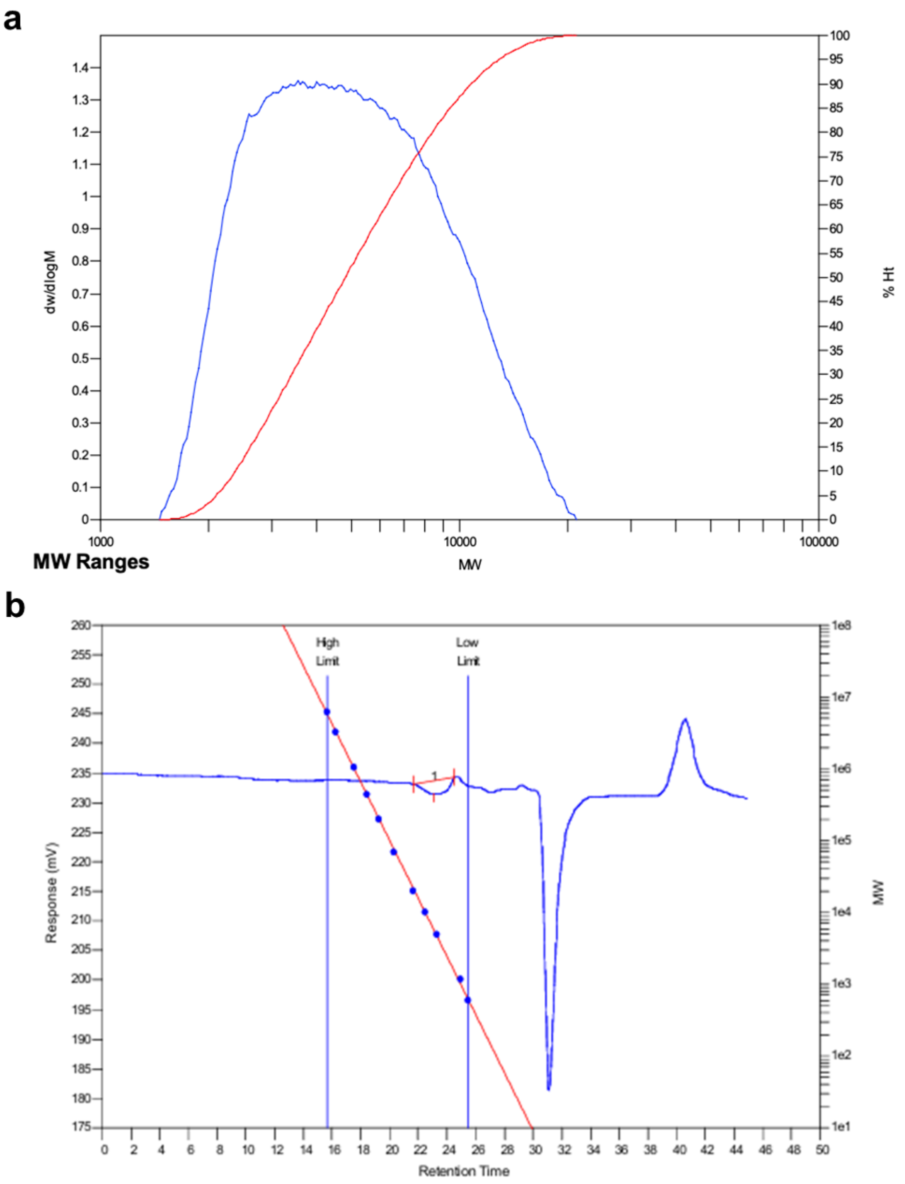

Supplementary Figure 5. GPC distribution Plots(a) and GPC trace(b) of Pu2gT at 150°C in TCB. M_n = 4.21 kDa, M_w = 5.75 kDa, PDI = 1.36.

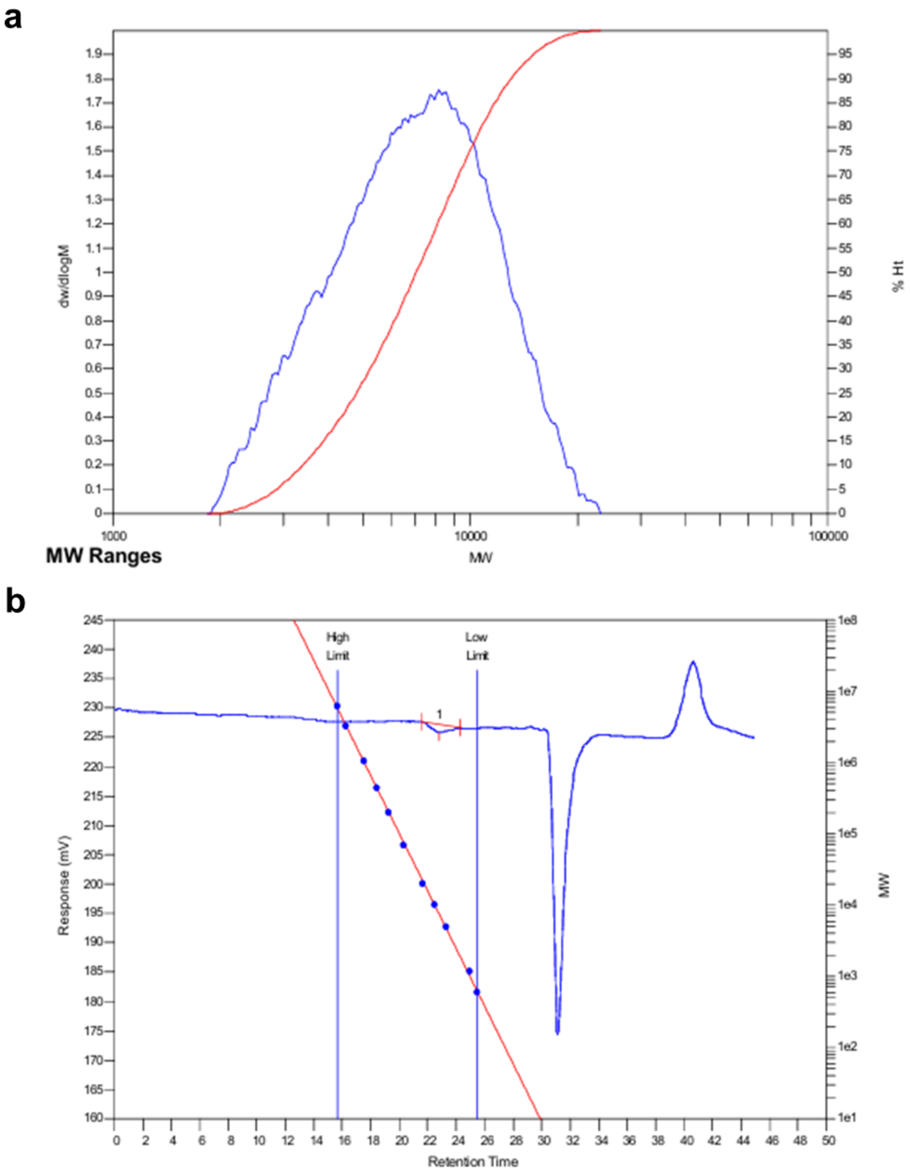

Supplementary Figure 6. GPC distribution Plots(a) and GPC trace(b) of Pu2gT at 150°C in TCB. M_n = 5.99 kDa, M_w = 7.67 kDa, PDI = 1.28.

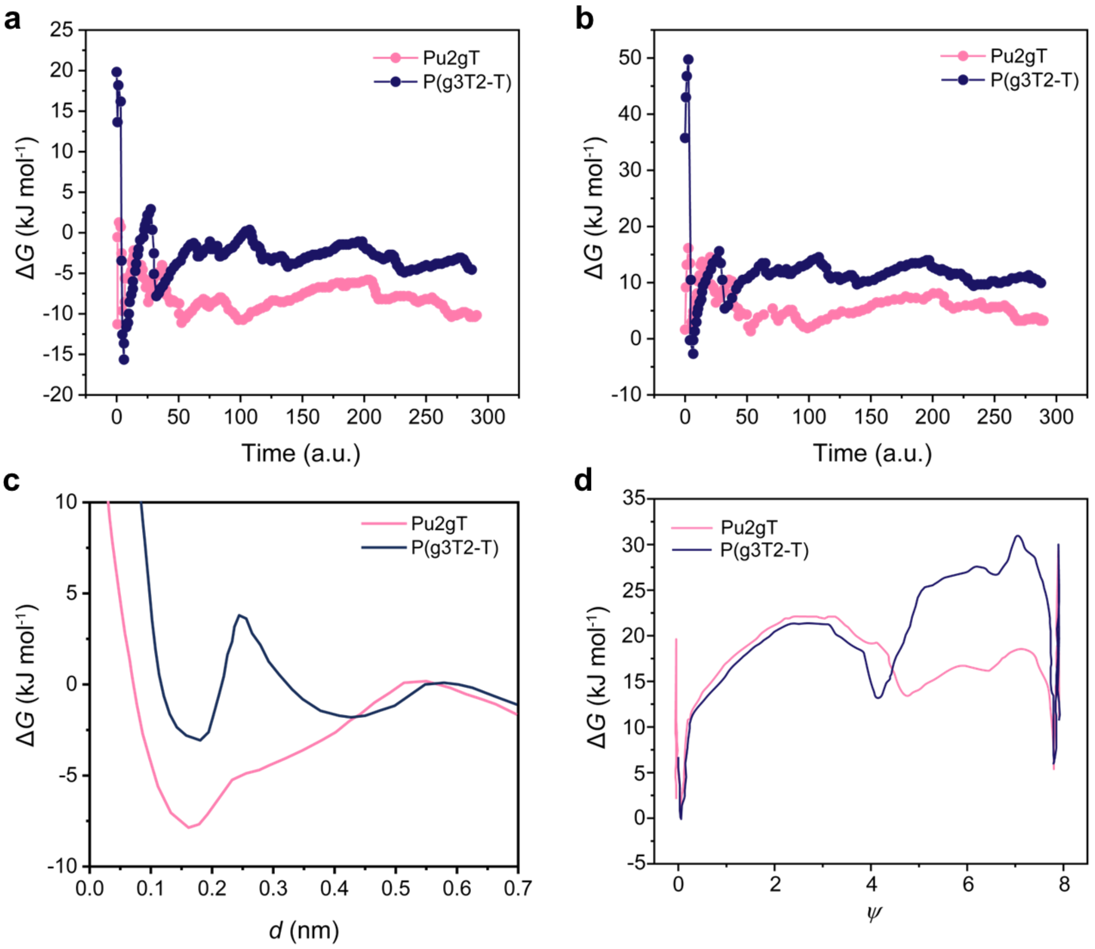

Supplementary Figure 7. Change in free-energy landscapes with time. a, The difference in free energy between the states d = 0.8 and d = 0.53 during the simulation time. **b**, the difference in free energy between the states ψ = 0.5 and ψ = 7.8. **c,** Free-energy landscape as a function of d. **d**, Free-energy landscape as a function of ψ. Error bars indicate the 95% confidence interval, calculated from the fluctuations of the free-energy surface over time.

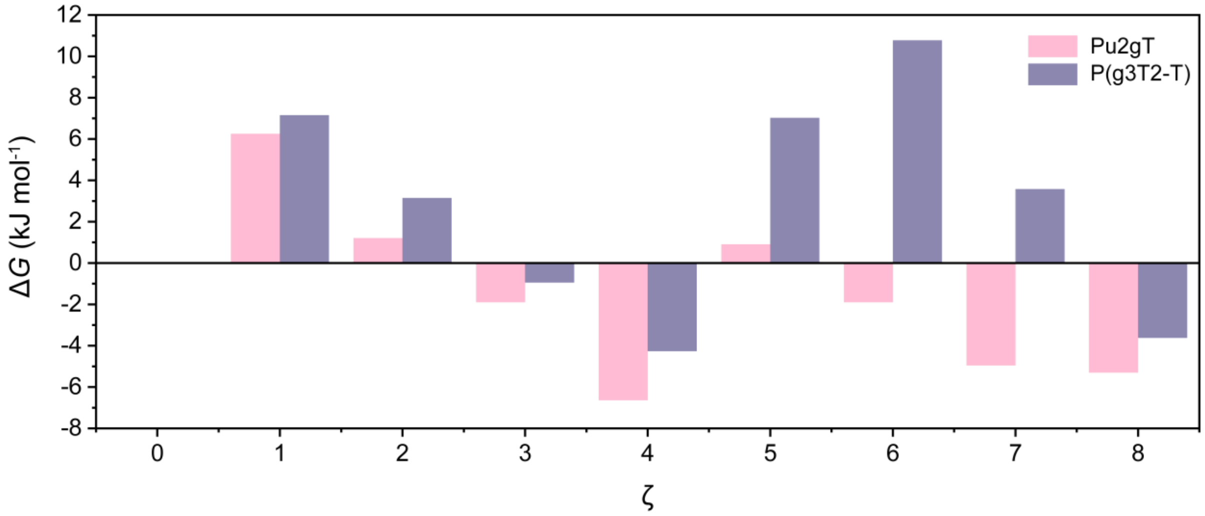

Supplementary Figure 8. Free-energetics of the interactions between Pu2gT or P(g3T2-T) units with a Na^+ cation in water. The free energy is plotted as a function of the coordination number between the cation and the oxygens on the polymer unit, ζ (with ζ = 0 being the zero-energy reference state).

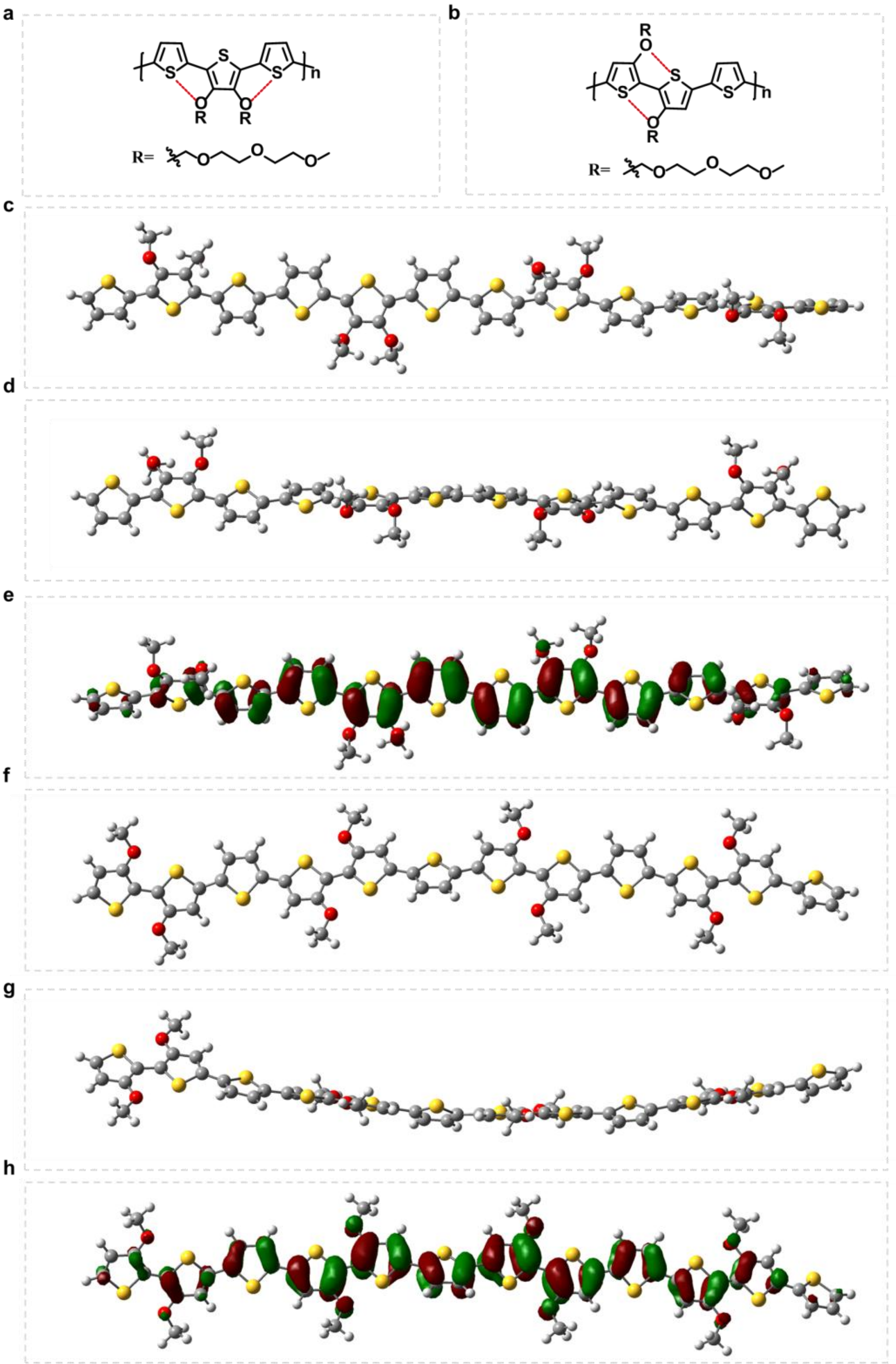

Supplementary Figure 9. Molecular structures and DFT-optimized, 4-repeat-unit-models of Pu2gT and P(g3T2-T). a,b, Molecular structures of Pu2gT (**a**) and P(g3T2-T) (**b**), highlighting their differences in S···O non-covalent interactions. **c–e**, Top-view geometry (**c**), side-view geometry (**d**), and frontier molecular orbitals (**e**) of the Pu2gT oligomer. The energy of the highest occupied molecular orbital (HOMO) is calculated to be −5.65 eV. **f–h**, Top-view geometry (**f**), side-view geometry (**g**), and frontier molecular orbitals (**h**) of the P(g3T2-T) oligomer. The HOMO energy is calculated to be −5.20 eV.

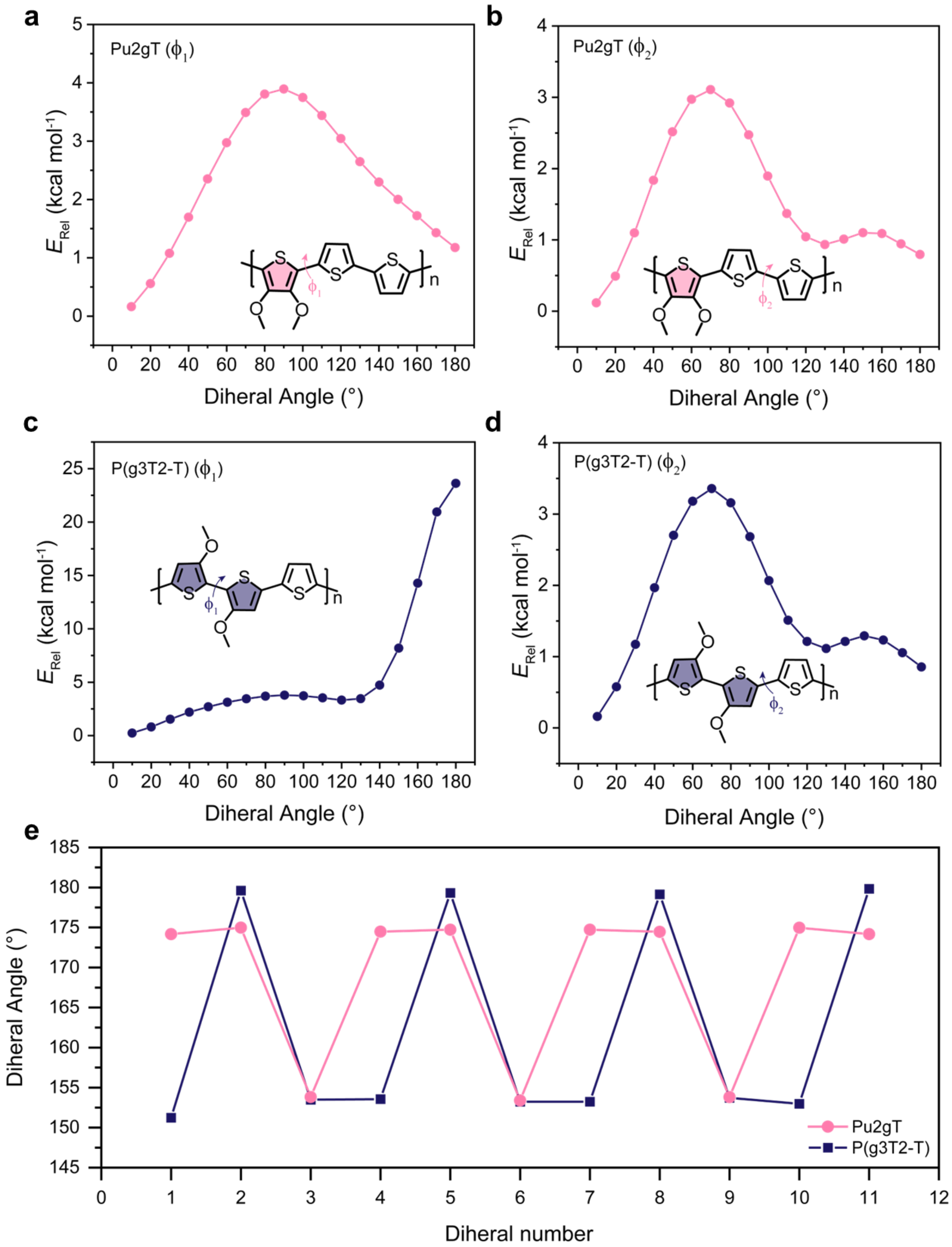

Supplementary Figure 10. Relative torsional potential (E_{rel}) profile and dihedral angle distribution of Pu2gT and P(g3T2-T). a,**b**, E_{rel} profile of Pu2gT with respect to φ_1 and φ_2 angles, representing regions with and without the S–O conformational lock, respectively. **c**,**d**, E_{rel} profile of P(g3T2-T) with respect to φ_1 and φ_2 angles. **e**, The dihedral angle distribution of Pu2gT and P(g3T2-T).

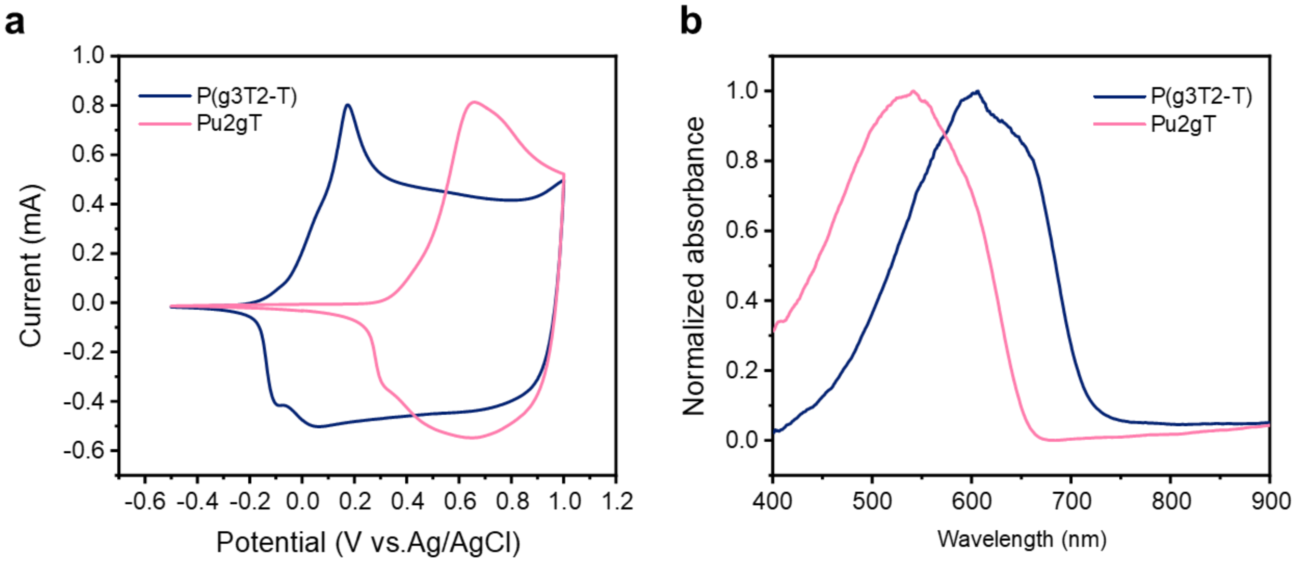

Supplementary Figure 11. Electrochemical characterization and energy level determination of Pu2gT and P(g3T2-T). a, Cyclic voltammograms of Pu2gT and P(g3T2-T) in 0.1 M tetrabutylammonium hexafluorophosphate ($TBAPF_6$) acetonitrile solution. The oxidation onset potentials for Pu2gT and P(g3T2-T) in $TBAPF_6$ electrolyte are 0.429 and −0.085 V, respectively, corresponding to HOMO energies of −4.87 and −4.37 eV. **b**, Normalized absorbance spectra of Pu2gT and P(g3T2-T) film. The absorption onsets are determined to be 655 and 719 nm for Pu2gT and P(g3T2-T), respectively, corresponding to optical gaps of 1.89 and 1.72 eV.

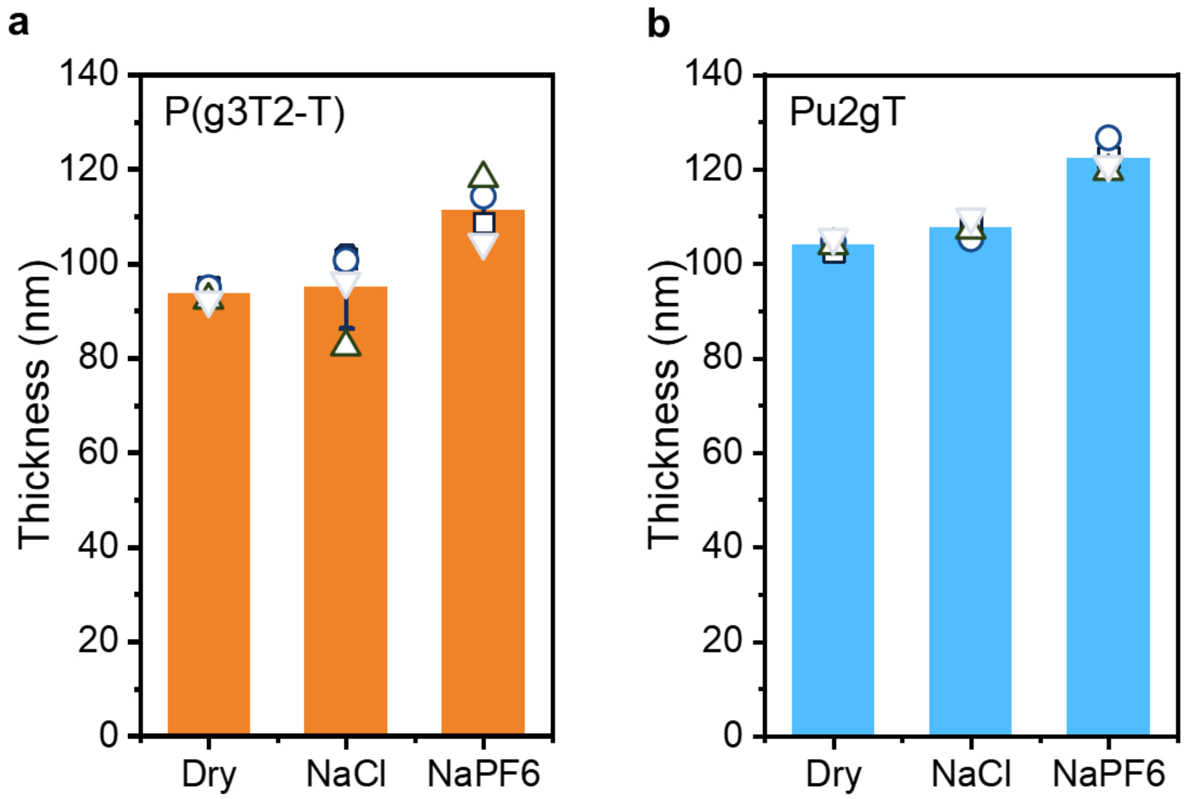

Supplementary Figure 12. Swelling propertites for P(g3T2-T) (a) and Pu2gT (b) in different electrolyte. All films show significant swelling in electrolytes $NaPF_6$. Data are shown as mean ± SD with individual data points overlaid.

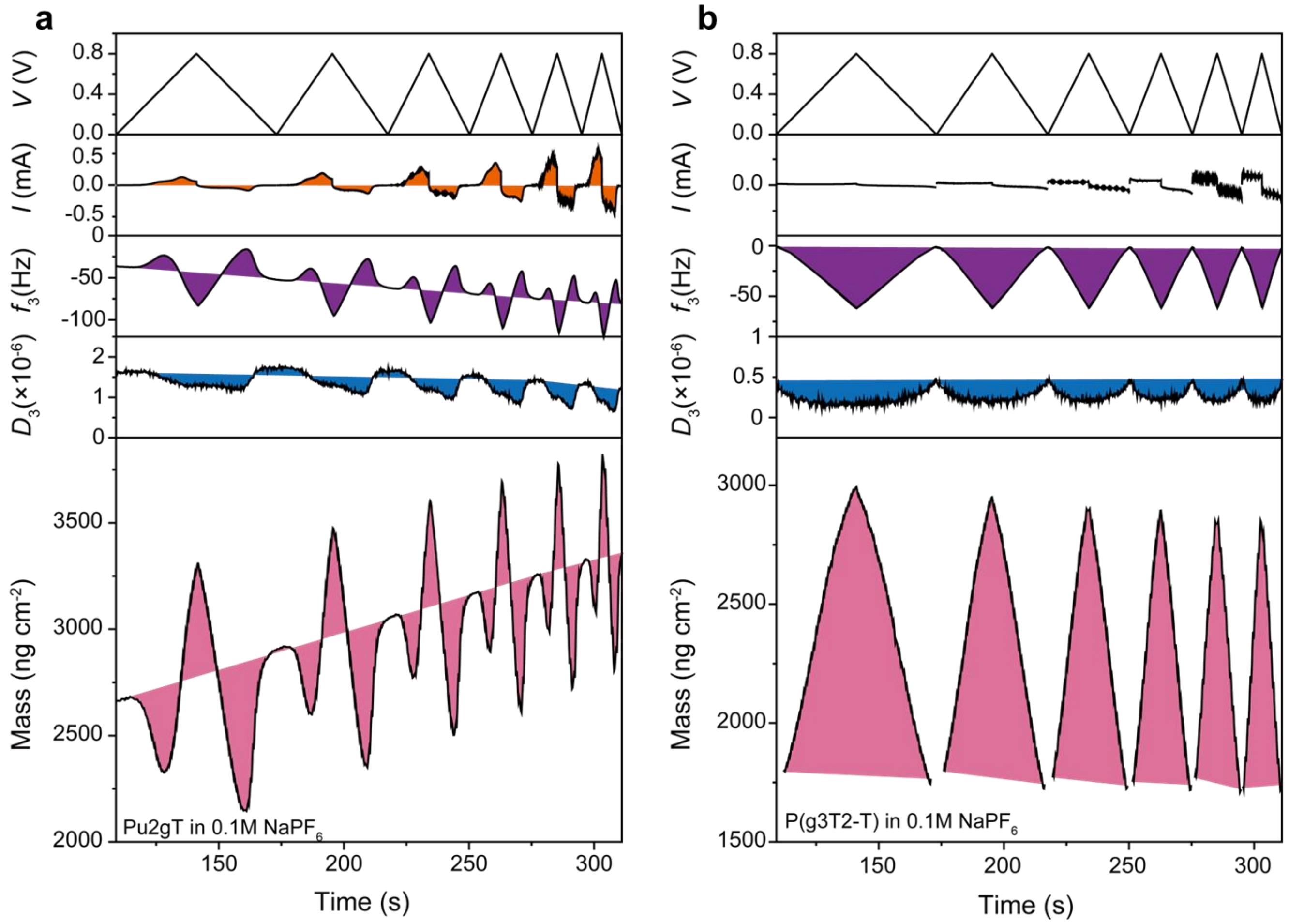

Supplementary Figure 13. EQCM-D results for Pu2gT(a) and P(g3T2-T) (b). The applied voltage profile, current response, QCM-D frequency (f_3) and dissipation (D_3) of the third overtone, and the derived mass change for Pu2gT in 0.1 M NaCl (**a**) and $NaPF_6$ (**b**) aqueous electrolyte are shown. The voltage scan rate varies from 0.025 to 0.1 V s^{-1}.

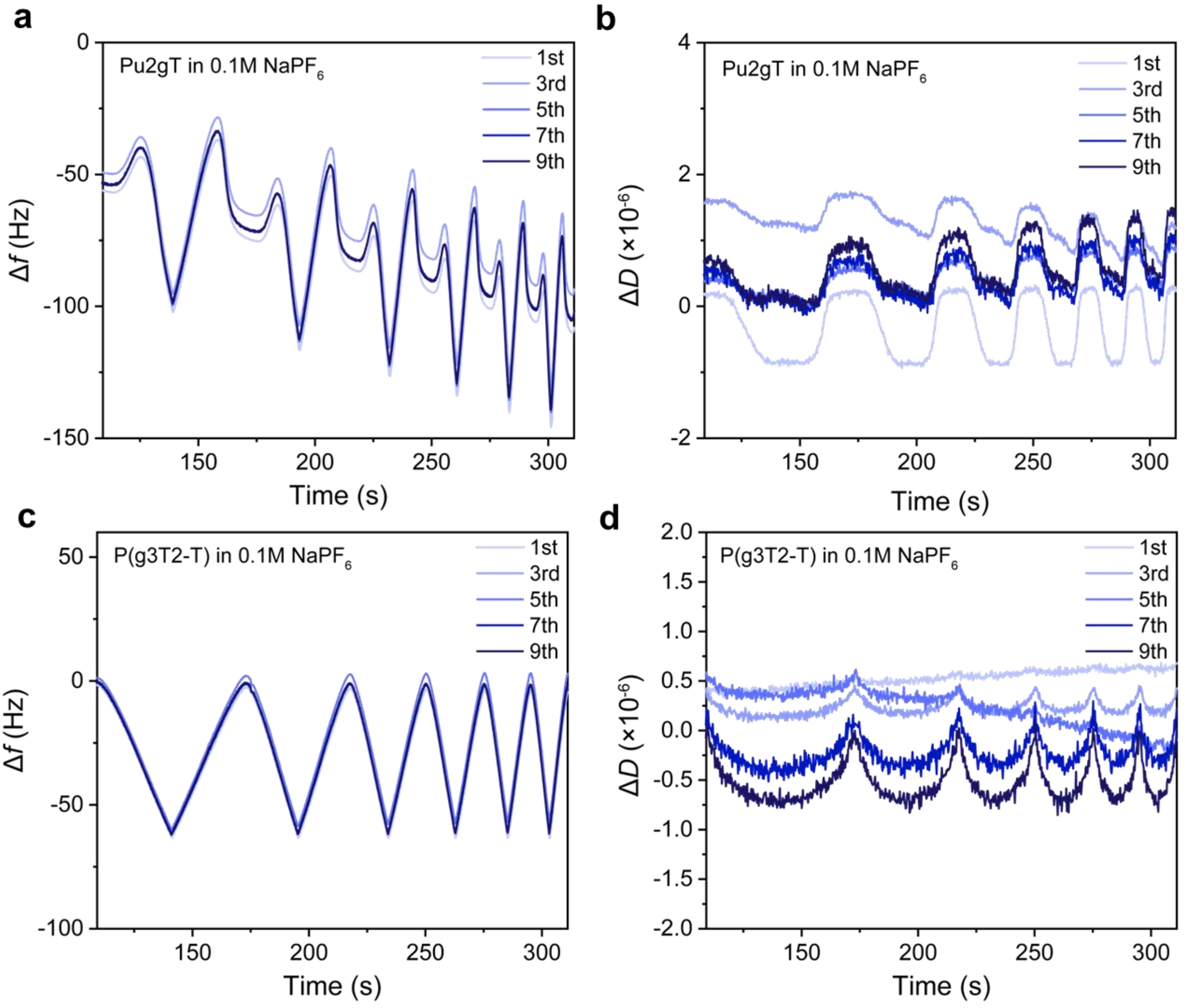

Supplementary Figure 14. Frequency shift (Δ*f*) and dissipation of energy (Δ*D*) for Pu2gT and P(g3T2-T) film in 0.1M $NaPF_6$ over different overtones. a,**b**, Δ*f* (**a**) and Δ*D* (**b**) for Pu2gT in 0.1 M $NaPF_6$ aqueous solution. **c**,**d**, Δ*f* (**c**) and Δ*D* (**d**) for P(g3T2-T) in 0.1 M $NaPF_6$ aqueous solution. The 1st, 3rd, 5th, 7th, and 9th overtones are shown.

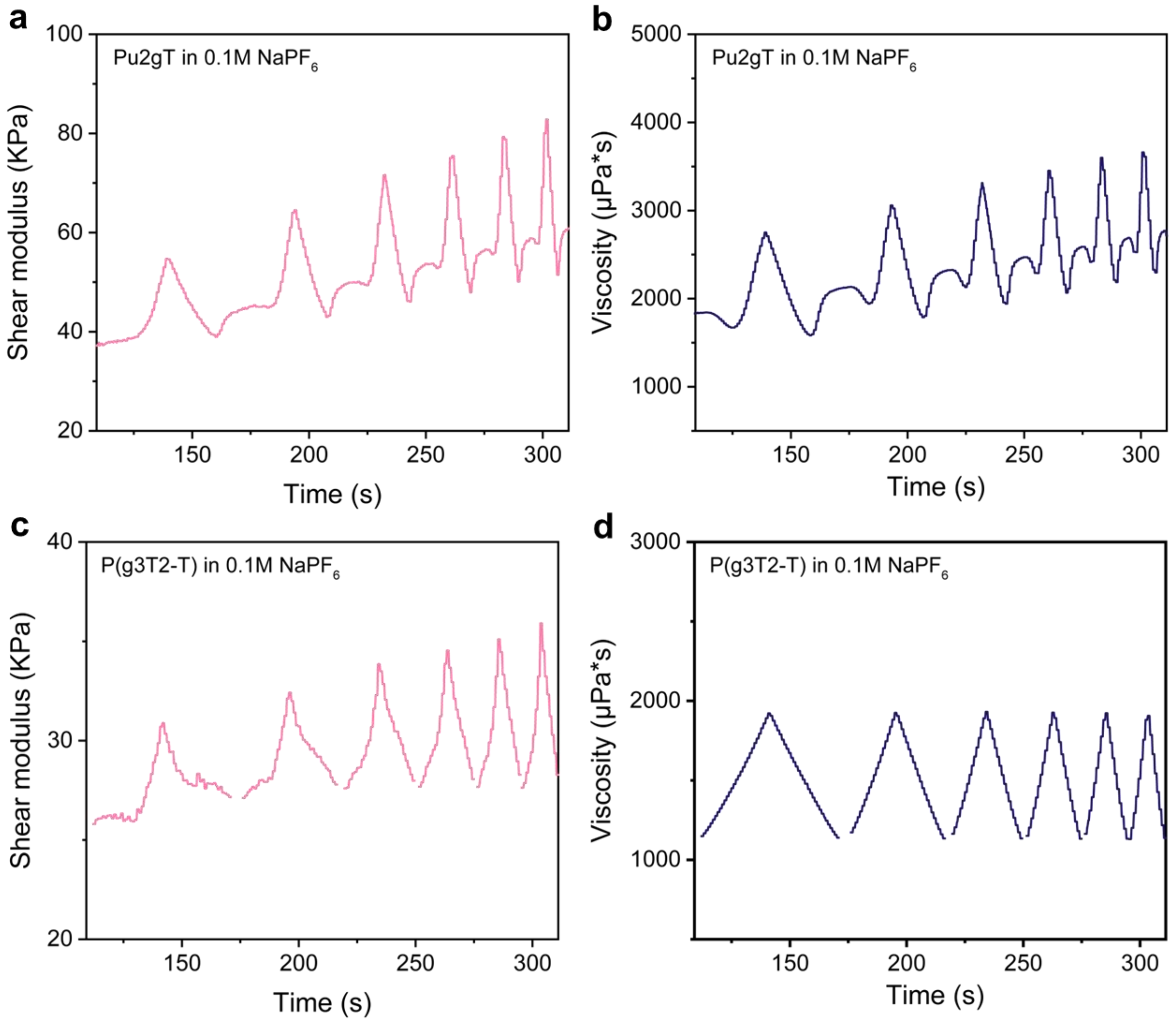

Supplementary Figure 15. Shear modulus and viscosity for Pu2gT and P(g3T2-T) film in different electrolytes. **a**,**b**, Shear modulus (**a**) and viscosity (**b**) for Pu2gT in 0.1 M $NaPF_6$ aqueous solution. **c**,**d**, Shear modulus (**c**) and viscosity (**d**) for P(g3T2-T) in 0.1 M $NaPF_6$ aqueous solution.

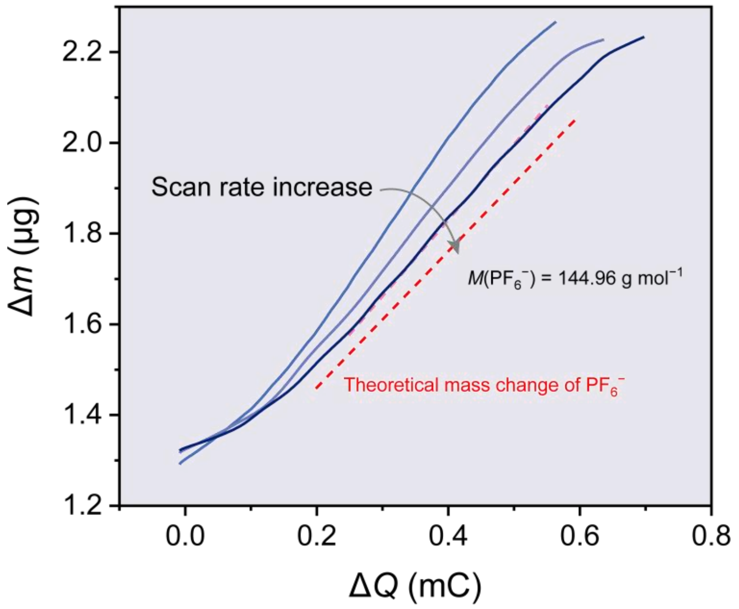

Supplementary Figure 16. The mass change of P(g3T2-T) film versus charge passed through the film in 0.1 M $NaPF_6$ aqueous electrolyte. The voltage scan rate is 0.064 V s^{-1}, 0.081 V s^{-1}, 0.1 V s^{-1}. Linear fitting is applied to extract the apparent molecular weight M_w^{app} to be 162.09 g mol^{-1}, larger than the molecular weight of PF_6^- (144.96 g mol^{-1}).

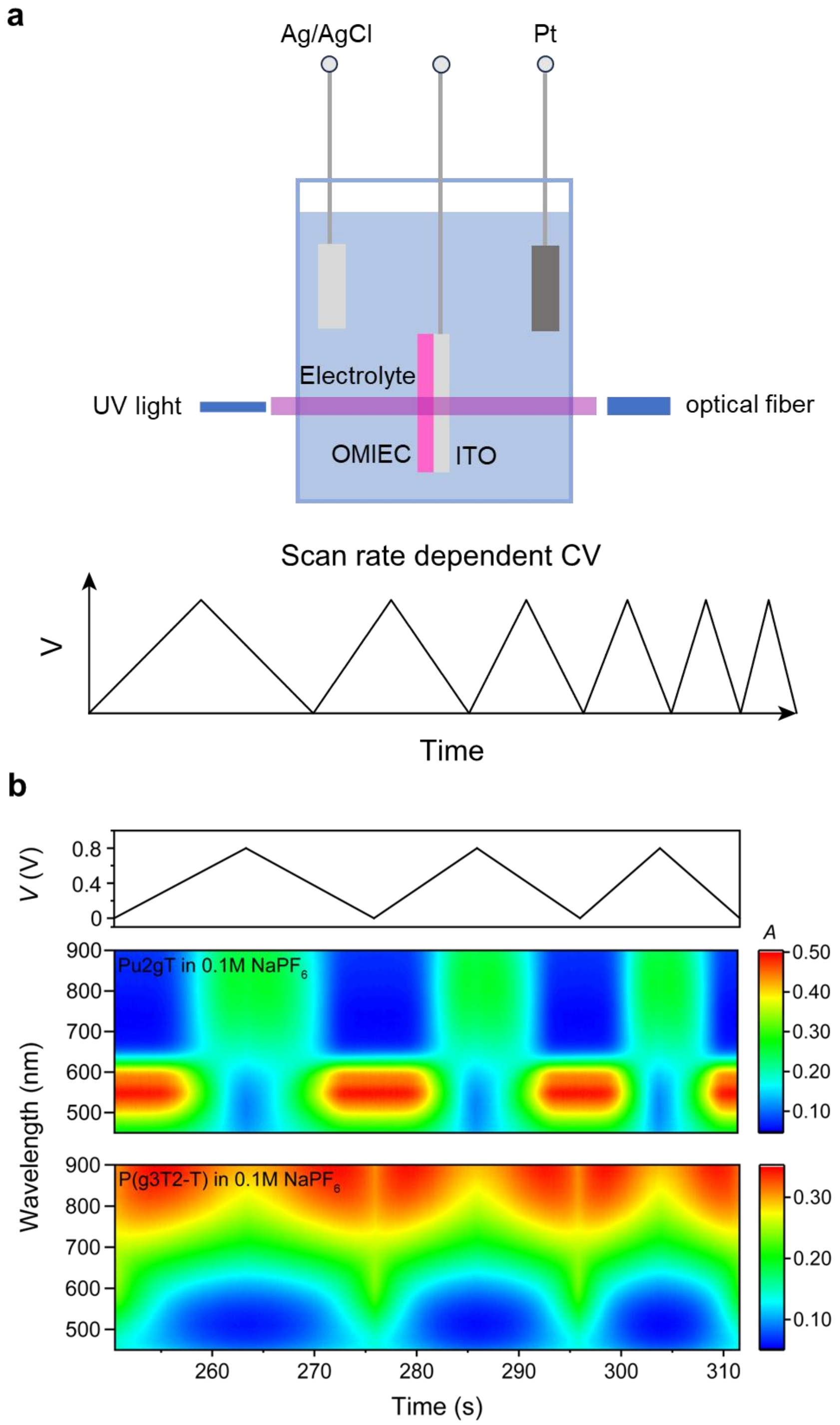

Supplementary Figure 17. Operando UV–vis–NIR absorption spectroscopy setup and results in 0.1 M $NaPF_6$ aqueous electrolyte. a, Measurement setup. The voltage range is 0 to 0.8 V, and the scan rates are 0.016, 0.025, 0.036, 0.049, 0.064, 0.081, 0.1, 0.15, 0.2, 0.25, and 0.3 V s^{-1}. **b**, Operando UV–vis–NIR absorption spectra for Pu2gT and P(g3T2-T) films during cyclic voltammetry. The electrolyte is 0.1 M $NaPF_6$ aqueous solution, and results corresponding to scan rates of 0.064, 0.081, and 0.1 V s^{-1} are shown.

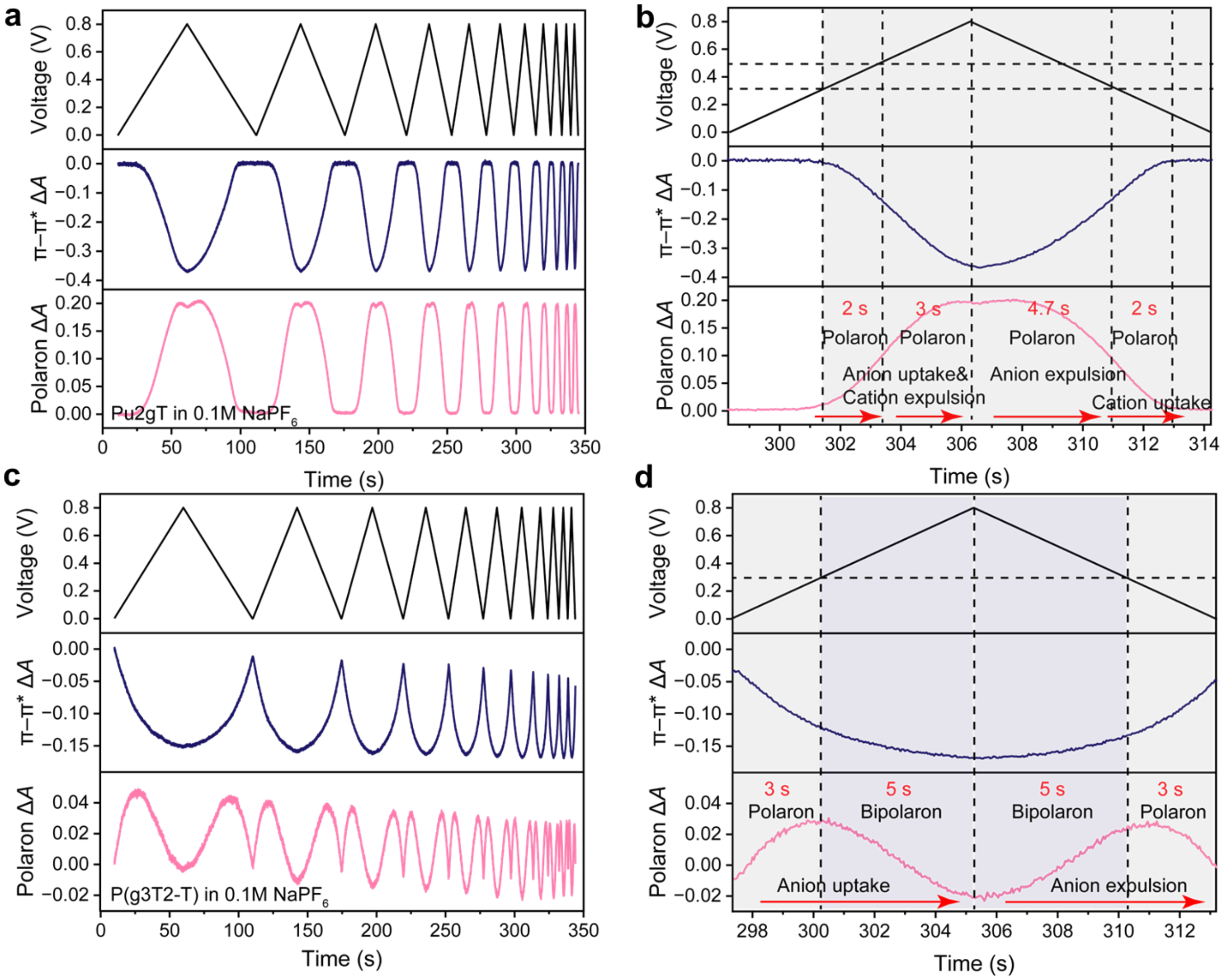

Supplementary Figure 18. Extraction of characteristic differential absorbance for Pu2gT. **a**, Differential absorbance of π–π* transition and polaron for Pu2gT in 0.1 M $NaPF_6$ aqueous electrolyte at different voltage scan rates. **b**, Detailed analysis of the 0.1 V s^{-1} scan in **a**. **c**, Differential absorbance of π–π* transition and polaron for P(g3T2-T) in 0.1 M $NaPF_6$ aqueous electrolyte at different voltage scan rates. **d**, Detailed analysis of the 0.1 V s^{-1} scan in **c**.

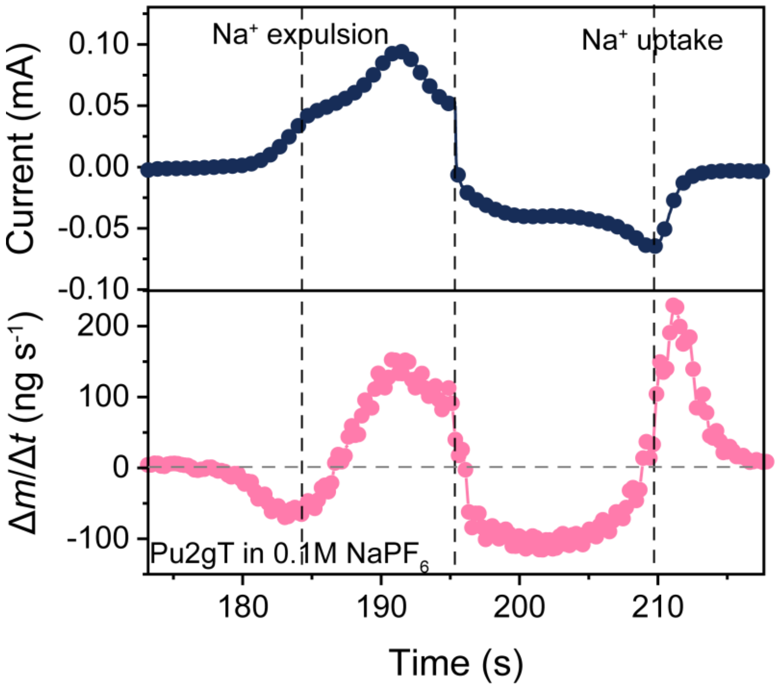

Supplementary Figure 19. Rate of Pu2gT mass change ($\Delta m/\Delta t$) against time at a scan rate of 0.036 V s^{-1} in 0.1M NaPF$_6$ aqueous solutions. Derivative of mass change ($\Delta m/\Delta t$) during a CV cycle. The peak in the forward scan corresponds to cation expulsion, and the peak in the reverse scan to cation re-uptake. The asymmetry (lower forward rate) confirms delayed cation expulsion.

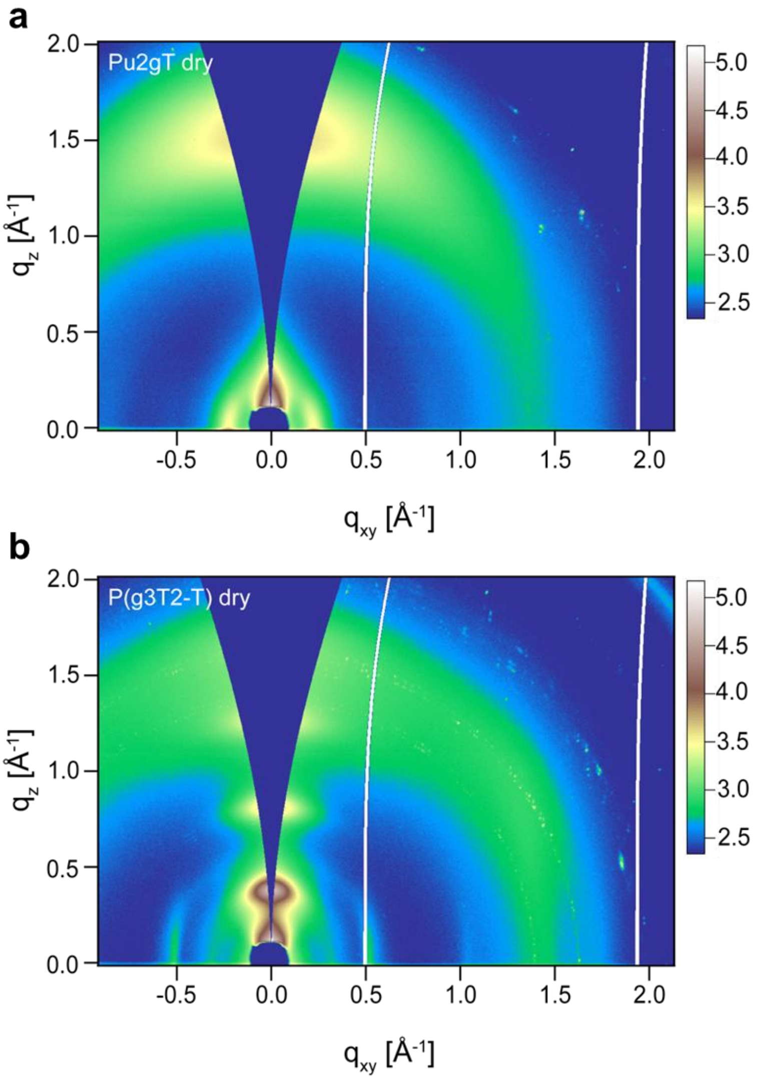

Supplementary Figure 20. Two-dimensional GIWAXS images of Pu2gT and P(g3T2-T) films. Pu2gT shows the face-on orientation and P(g3T2-T) shows the mixed face-on and edge-on orientation.

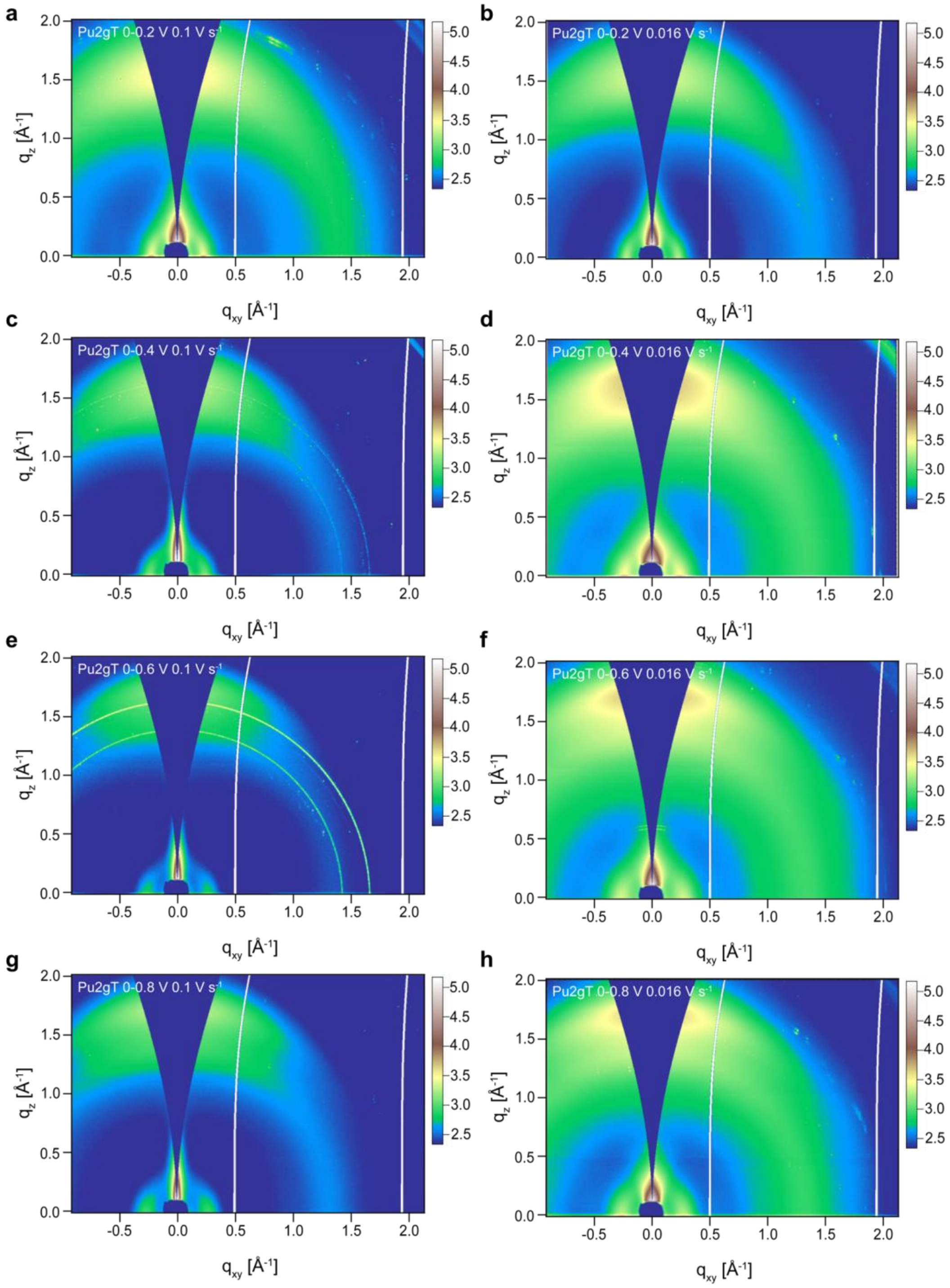

Supplementary Figure 21. Two-dimensional GIWAXS images. Pu2gT film in different scan rate and voltage range. The patterns show scan-rate-dependent microstructural changes.

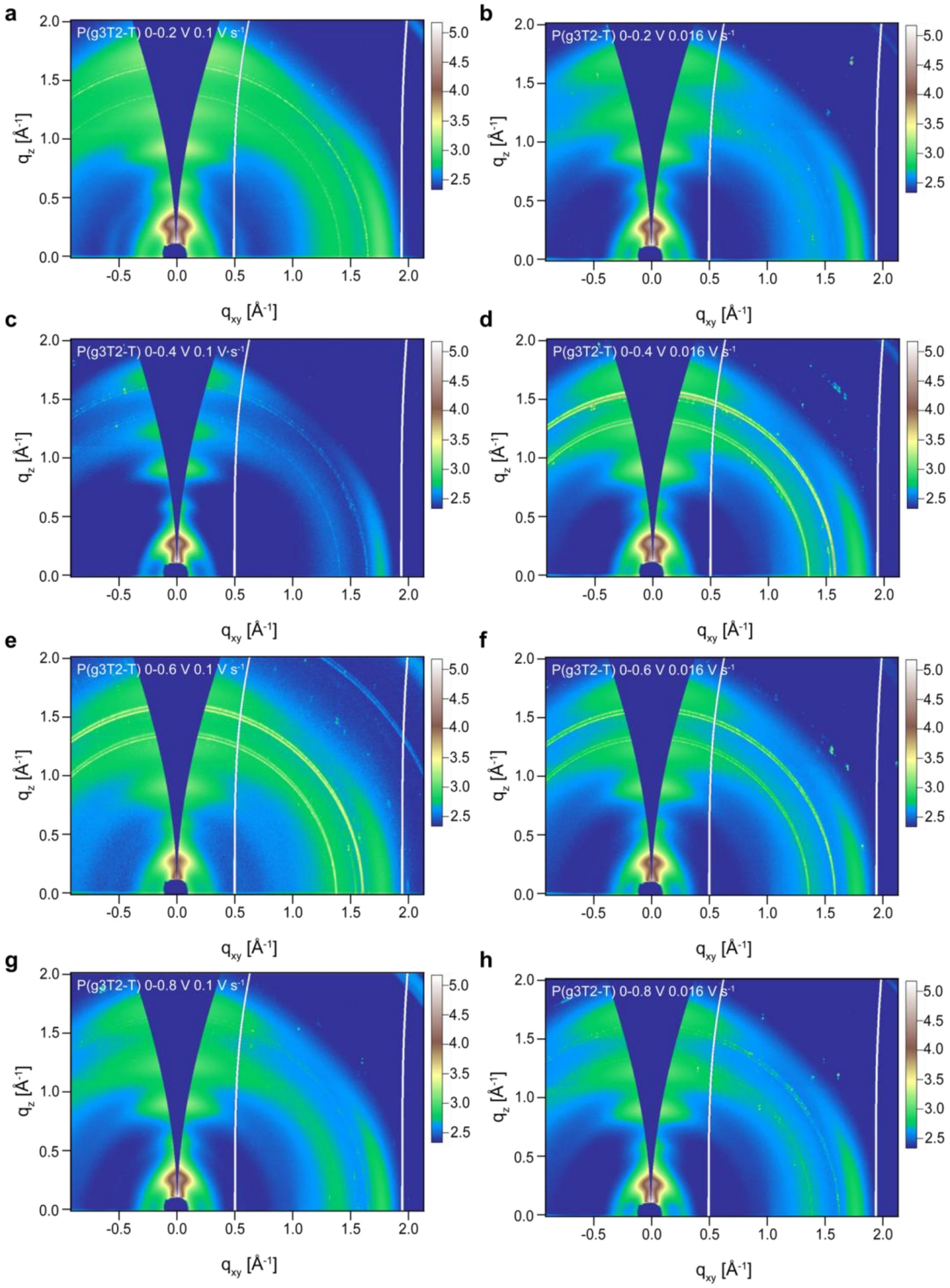

Supplementary Figure 22. Two-dimensional GIWAXS images. P(g3T2-T) film in different scan rate and voltage range. The patterns show less scan-rate-dependent microstructural changes.

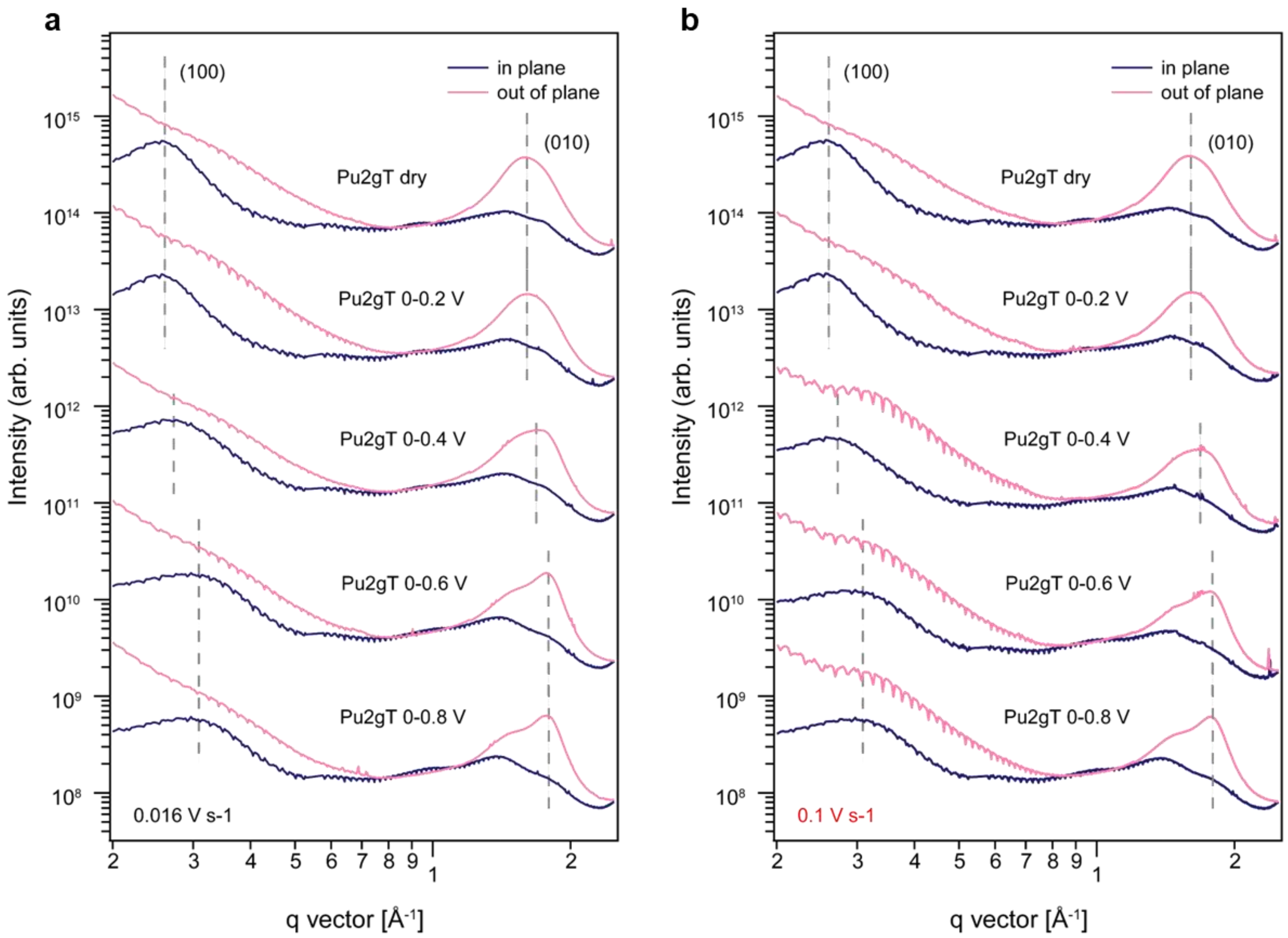

Supplementary Figure 23. One-dimensional GIWAXS linecuts. Pu2gT in 0.016 V s^{-1}(**a**) and 0.1 V s^{-1}(**b**) with different voltage range. 1D GIWAXS linecuts (IP and OOP) for Pu2gT at low (0.016 V s^{-1}, **a**) and high (0.1 V s^{-1}, **b**) scan rates. The (100) lamellar peak shifts with voltage, reflecting different cation expulsion kinetics.

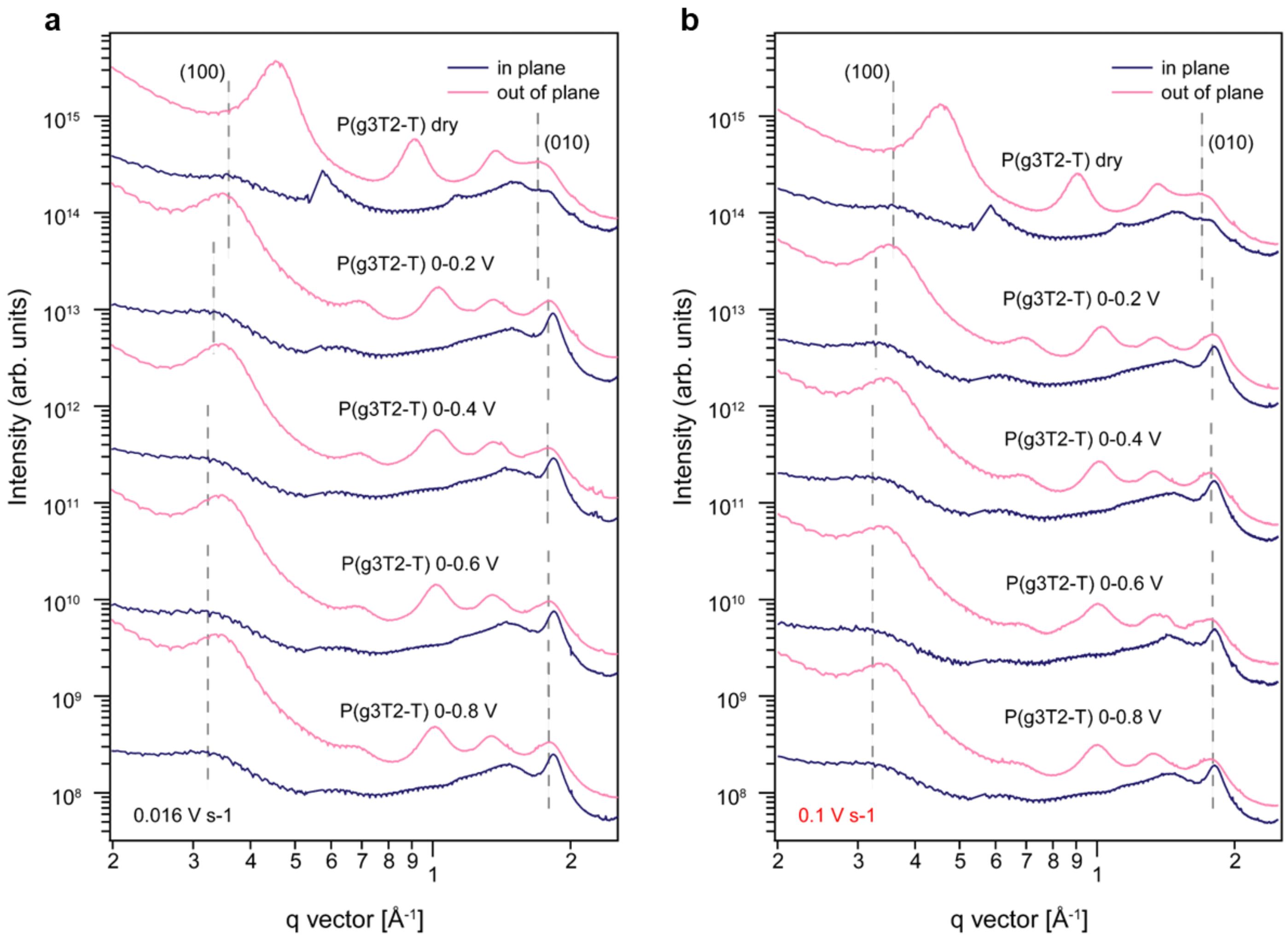

Supplementary Figure 24. One-dimensional GIWAXS linecuts. P(g3T2-T) in 0.016 V s^{-1}(**a**) and 0.1 V s^{-1}(**b**) with different voltage range. The (100) peak gradually shifts to lower q (increased d-spacing) with increasing voltage, independent of scan rate, consistent with anion-only doping.

Supplementary Table 1. Summary of in-plane (IP) and out-of-plane (OOP) peak fitting results from one-dimensional GIWAXS linecuts.

Sample and Voltage	Scan rate ($V s^{-1}$)	Peak location ($Å^{-1}$)	*d*-spacing[a] (Å)	FWHM[b] ($Å^{-1}$)	CCL[c] (Å)
Pu2gT 0.2 V (IP)	0.1	0.239	26.29	0.078	72.50
Pu2gT 0.2 V (OOP)		1.603	3.920	0.373	15.16
Pu2gT 0.4 V (IP)		0.248	25.34	0.100	56.55
Pu2gT 0.4 V (OOP)		1.685	3.729	0.393	14.39
Pu2gT 0.6 V (IP)		0.271	23.19	0.172	32.88
Pu2gT 0.6 V (OOP)		1.756	3.578	0.265	21.34
Pu2gT 0.8 V (IP)		0.275	22.85	0.168	33.66
Pu2gT 0.8 V (OOP)		1.769	3.551	0.258	21.92
Pu2gT 0.2 V (IP)	0.016	0.240	26.18	0.078	72.50
Pu2gT 0.2 V (OOP)		1.617	3.886	0.405	13.96
Pu2gT 0.4 V (IP)		0.253	24.83	0.129	48.84
Pu2gT 0.4 V (OOP)		1.714	3.666	0.348	16.25
Pu2gT 0.6 V (IP)		0.278	22.60	0.159	35.57
Pu2gT 0.6 V (OOP)		1.772	3.546	0.273	20.71
Pu2gT 0.8 V (IP)		0.277	22.68	0.165	34.27
Pu2gT 0.8 V (OOP)		1.772	3.546	0.278	20.34
P(g3T2-T) 0.2 V (IP)	0.1	0.338	18.59	0.161	35.12
P(g3T2-T) 0.2 V (OOP)		1.794	3.502	0.240	23.56
P(g3T2-T) 0.4V (IP)		0.333	18.87	0.175	32.31
P(g3T2-T) 0.4 V (OOP)		1.794	3.502	0.277	20.41

P(g3T2-T) 0.6 V (IP)		0.325	19.33	0.183	30.90
P(g3T2-T) 0.6 V (OOP)		1.797	3.496	0.272	20.79
P(g3T2-T) 0.8 V (IP)		0.322	19.51	0.176	32.13
P(g3T2-T) 0.8 V (OOP)		1.800	3.491	0.317	17.84
P(g3T2-T) 0.2 V (IP)	0.016	0.335	18.76	0.143	39.54
P(g3T2-T) 0.2 V (OOP)		1.794	3.502	0.271	20.87
P(g3T2-T) 0.4 V (IP)		0.333	18.87	0.191	29.61
P(g3T2-T) 0.4 V (OOP)		1.802	3.487	0.279	20.27
P(g3T2-T) 0.6 V (IP)		0.333	18.87	0.188	30.08
P(g3T2-T) 0.6 V (OOP)		1.805	3.481	0.255	22.18
P(g3T2-T) 0.8 V (IP)		0.330	19.04	0.193	32.56
P(g3T2-T) 0.8 V (OOP)		1.802	3.487	0.293	19.30

[a]*d*-spacing is calculated by 2π / (peak position). [b]FWHM denotes full width at half maximum. [c]CCL denotes crystalline coherence length and is calculated by CCL = 2π × 0.9 / FWHM.

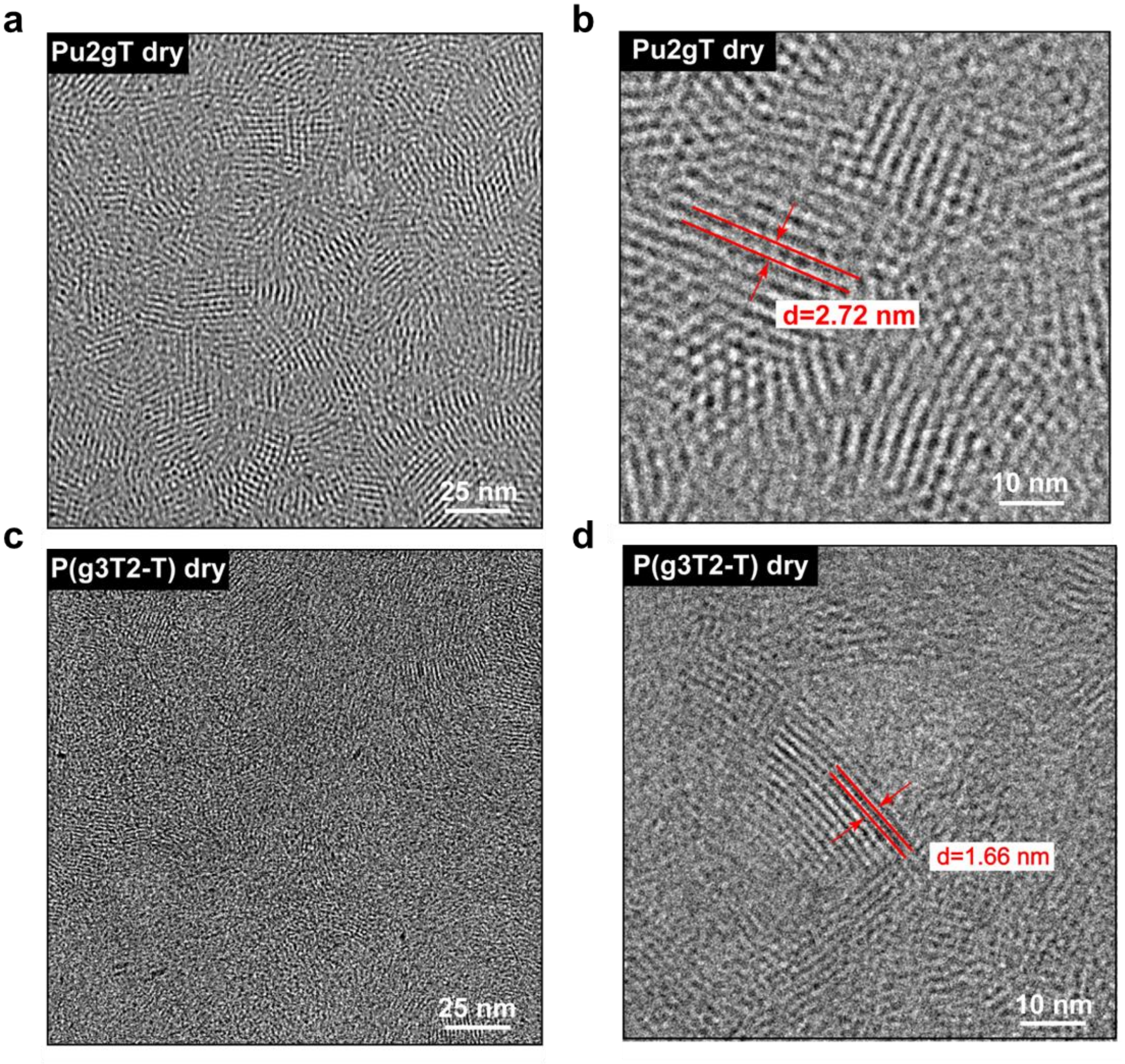

Supplementary Figure 25. Cryo-EM images for Pu2gT (a) and P(g3T2-T) (c) films. The scale bar is 25 nm. And **b,d** are the partial enlarged image of **a,b**. The scale bar is 10 nm, and extracted fringe spacings are indicated.

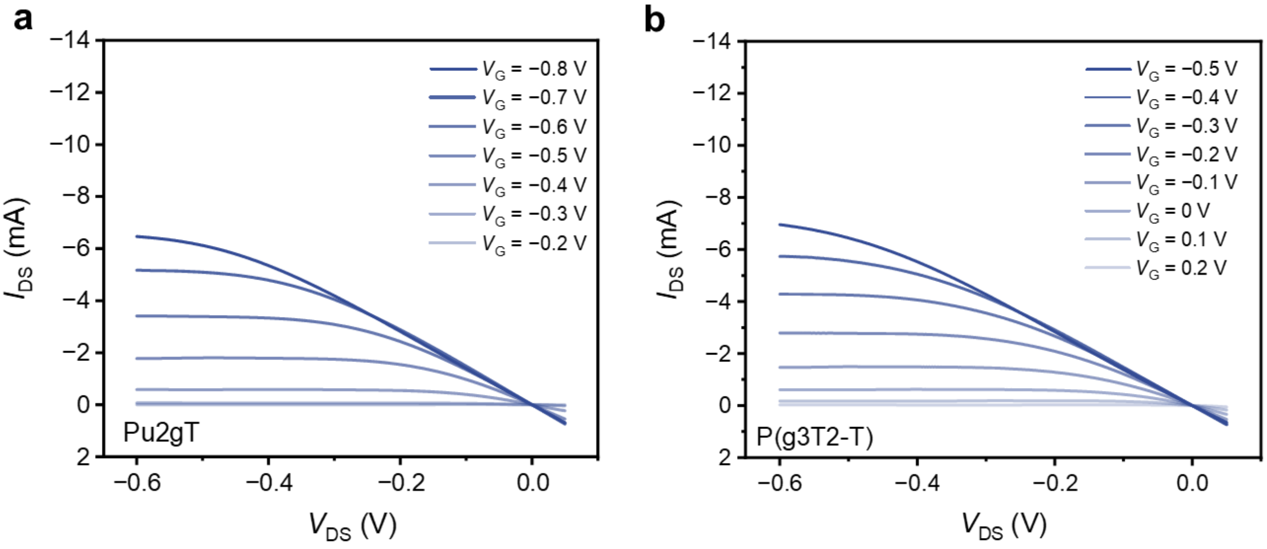

Supplementary Figure 26. c-OECT performance. a,**b**, Output characteristics for Pu2gT (**a**) and P(g3T2-T) (**b**) c-OECTs.

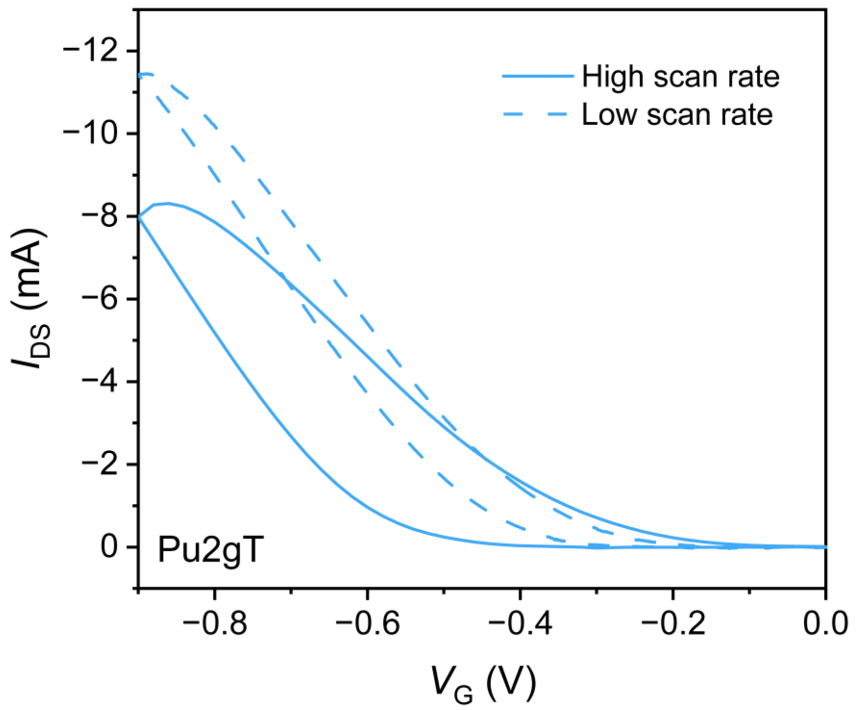

Supplementary Figure 27. Scan rate performance. Transfer curve of Pu2gT film in different scan rate.

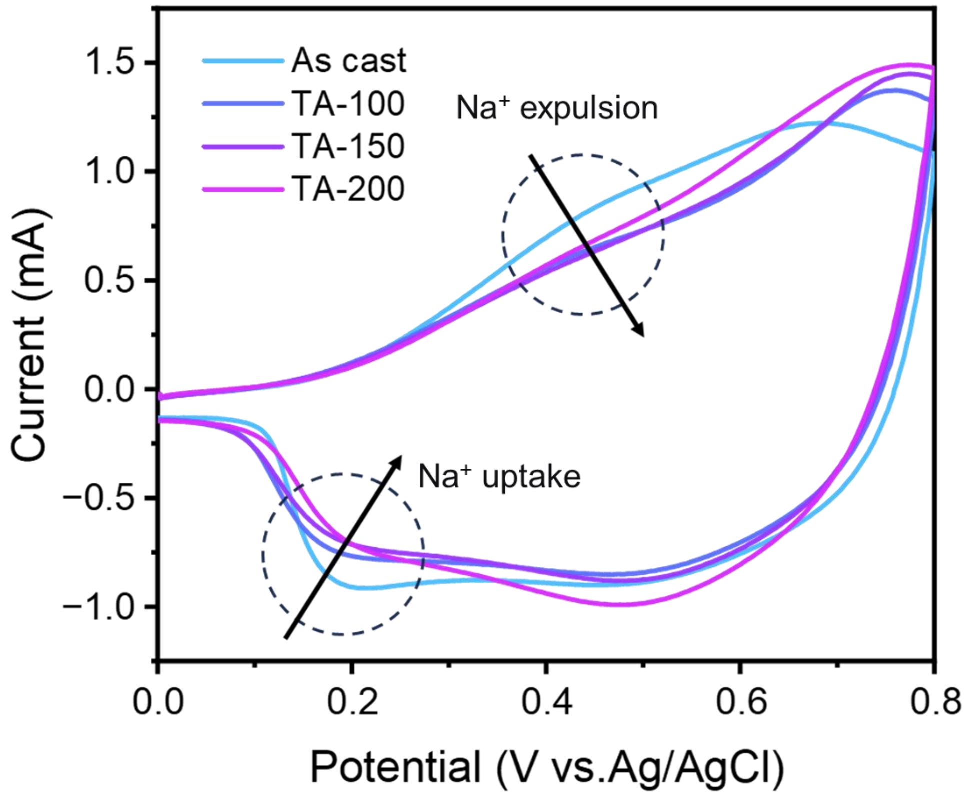

Supplementary Figure 28. Cyclic voltammograms after annealing. Pu2gT samples after different annealing temperatures (TA).

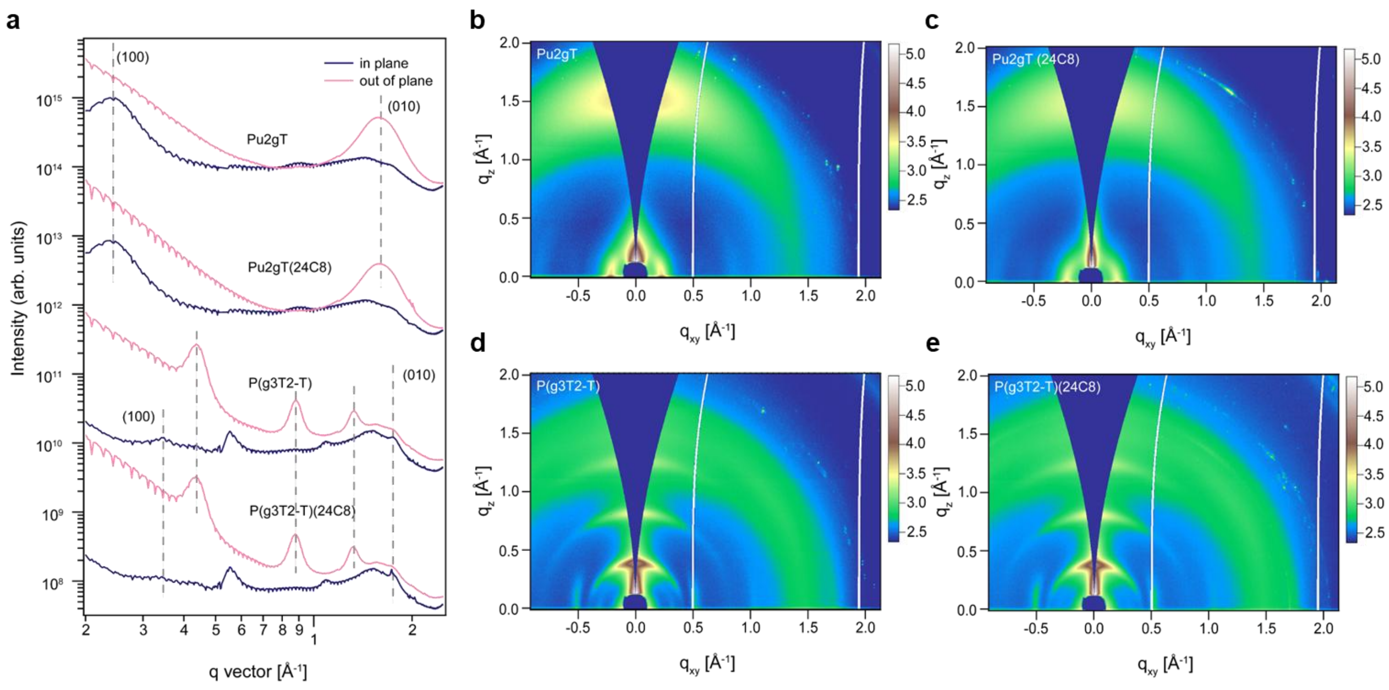

Supplementary Figure 29. Molecular packing characterization of Pu2gT, Pu2gT(24C8), P(g3T2-T) and P(g3T2-T)(24C8). a, One-dimensional GIWAXS linecuts of Pu2gT, Pu2gT(24C8), P(g3T2-T) and P(g3T2-T)(24C8) films. **b**,**c**, Two-dimensional GIWAXS images of Pu2gT (**b**) and Pu2gT(24C8) films (**c**). **d**,**e**, Two-dimensional GIWAXS images of P(g3T2-T) (**b**) and P(g3T2-T) (24C8) films (**c**).

Supplementary Table 2. GIWAXS fit-result of Pu2gT, Pu2gT(24C8) films.

	**Location ($Å^{-1}$)**	**d-spacing (nm)**	**FWHM**	**CL (nm)**
Pu2gT (100)	0.24	2.618	0.067	8.44
Pu2gT(24C8) (100)	0.24	2.618	0.067	8.44
Pu2gT (010)	1.59	0.3951	0.37	1.53
Pu2gT(24C8) (010)	1.59	0.3951	0.37	1.53

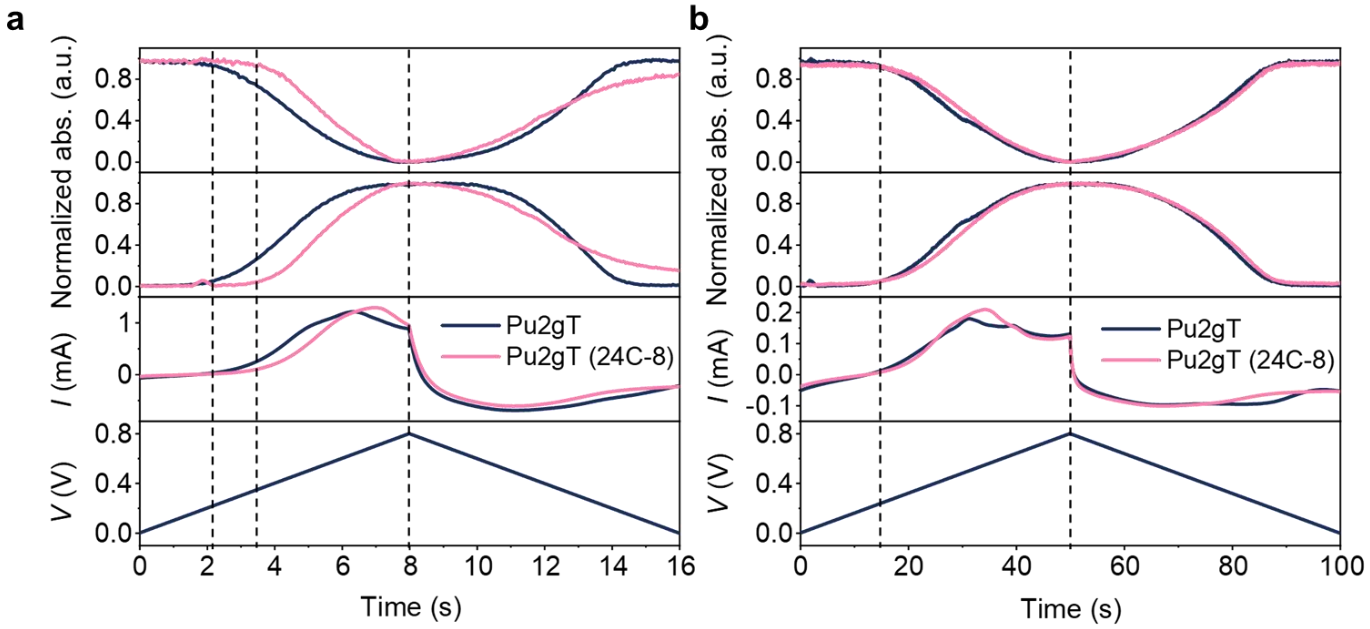

Supplementary Figure 30. Operando UV–vis–NIR absorption and CV results for Pu2gT and Pu2gT(24C8) in different scan rate (a) 0.1 V s^{-1} and (b) 0.016V s^{-1}. The polaron absorbance was extracted at 850 nm for Pu2gT and Pu2gT(24C8) spectra.

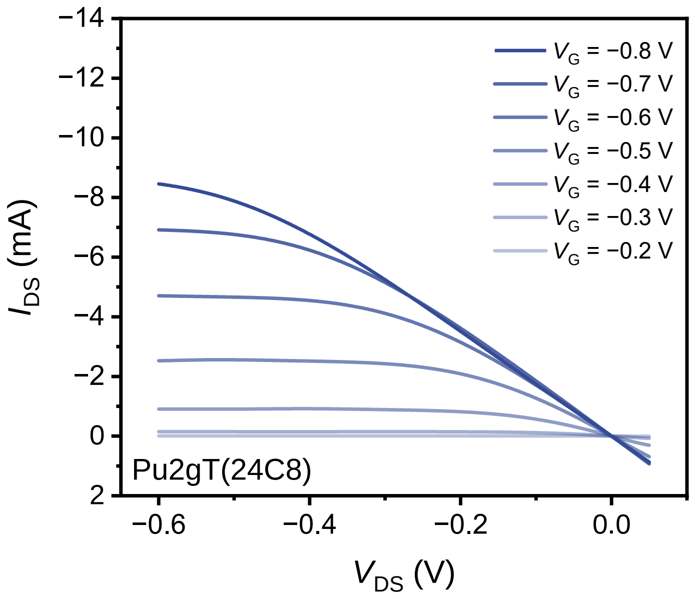

Supplementary Figure 31. Output performance. c-OECT output characteristics for Pu2gT(24C8).

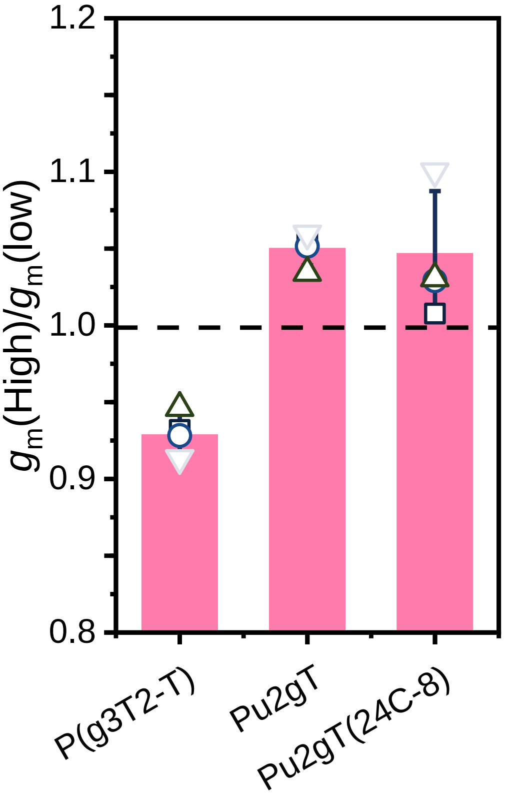

Supplementary Figure 32. Statistical Comparison of Transconductance Ratios. Between High and Low Scan Rates for Identical Devices. Data are shown as mean ± SD with individual data points overlaid.

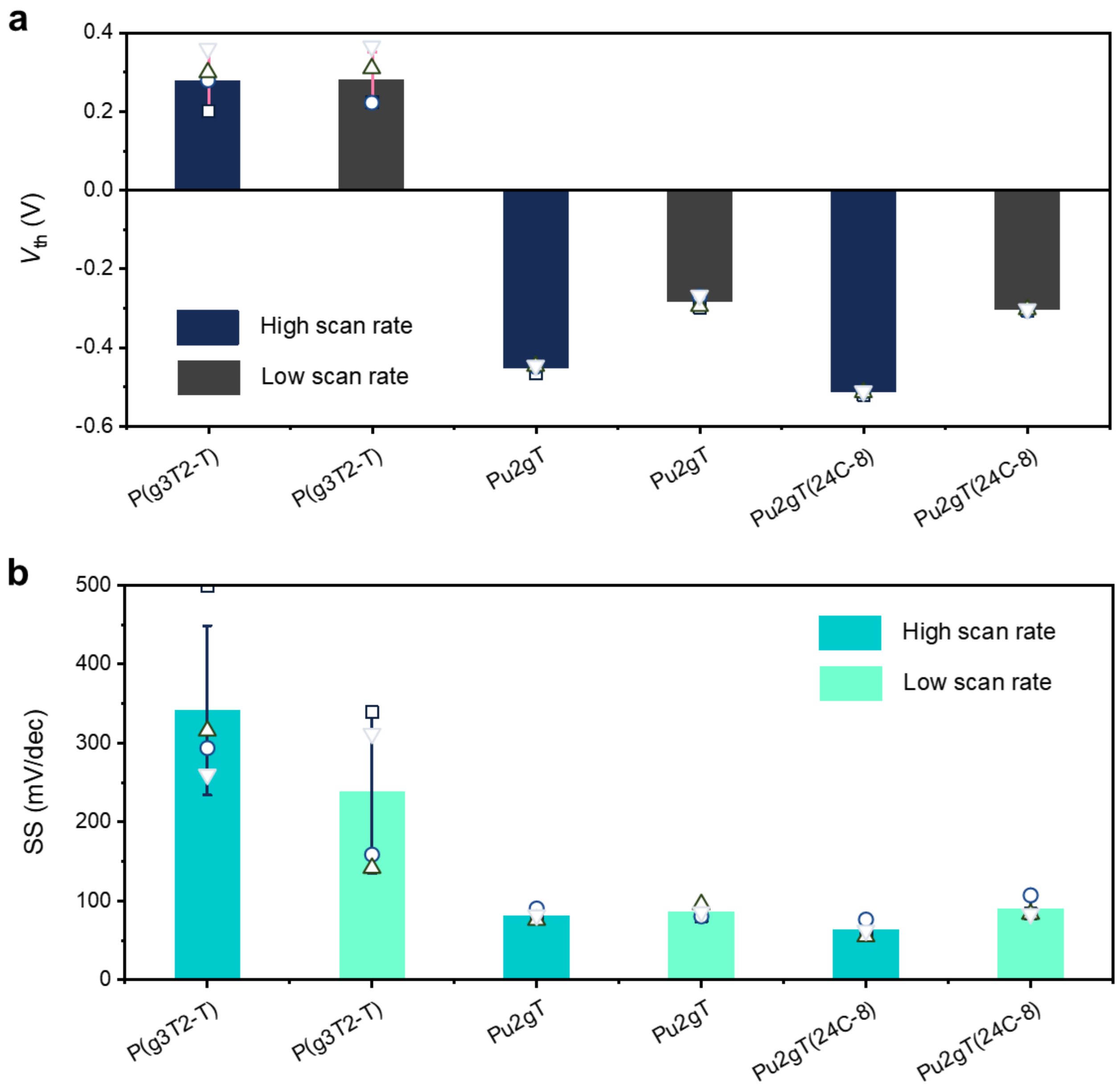

Supplementary Figure 33. Statistical Comparison. Threshold Voltages(a) and Subthreshold Slopes(b) for P(g3T2-T), Pu2gT, and Pu2gT(24C8) under High and Low Scan Rates. Data are shown as mean ± SD with individual data points overlaid.

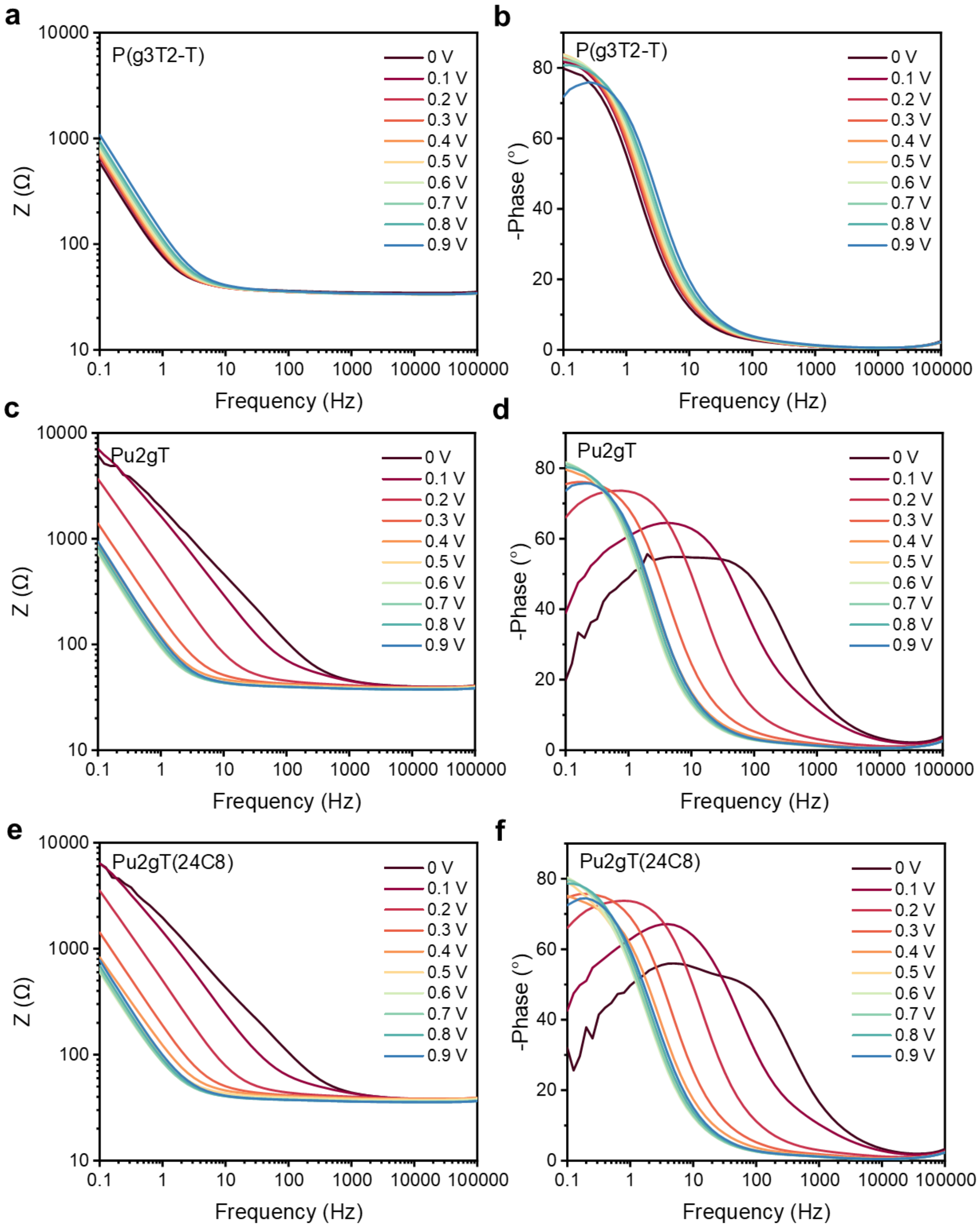

Supplementary Figure 34. Electrochemical impedance spectroscopy (EIS) results and *C extraction. a**,**b**, Representative frequency-dependent impedance (*Z*) and phase for P(g3T2-T). **c**,**d**, Representative frequency-dependent impedance (*Z*) and phase for Pu2gT. **e**,**f**, Representative frequency-dependent impedance (*Z*) and phase for Pu2gT(24C8).

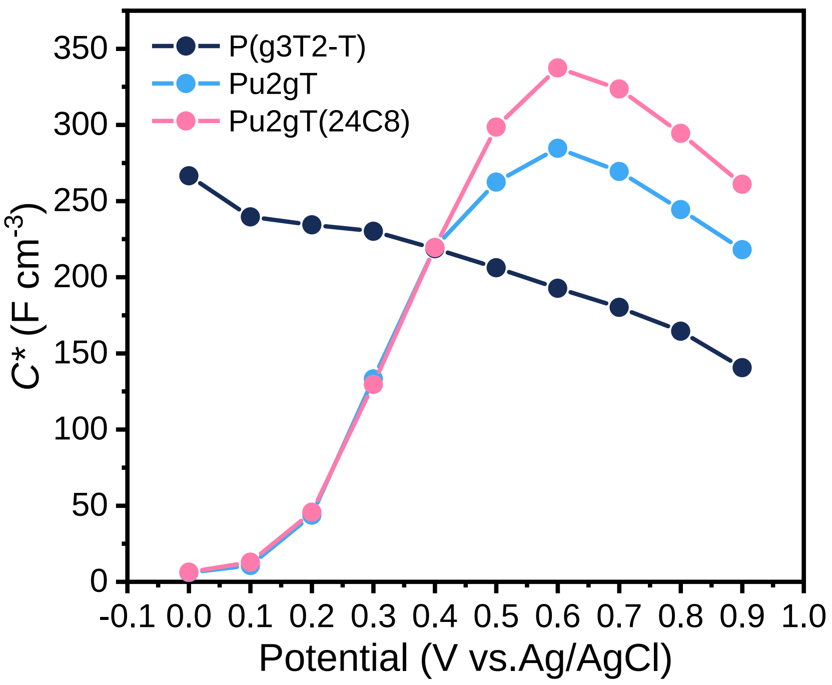

Supplementary Figure 35. Electrochemical performance. Extracted volumetric capacitance C^* as a function of applied DC bias (0–0.9 V) for P(g3T2-T), Pu2gT, and Pu2gT(24C8). Pu2gT(24C8) displays the highest C^* across all voltages, consistent with enhanced dual-doping.

Supplementary Table 3. Summary of c-OECT parameters[a].

	V_{th} (V)[b]	SS (mV/dec)	g_m (mS)	$g_m/(Wd/L)$ (S cm^{-1})
Pu2gT	−0.45 ± 0.01	81.12 ± 7.73	25.79 ± 5.17	241.78 ± 48.5
Pu2gT (24C8)	−0.514 ± 0.005	62.74 ± 9.25	39.34 ± 6.97	368.81 ± 65.34
P(g3T2-T)	0.28 ± 0.08	341.47 ± 107.3	16.52 ± 1.37	154.87 ± 12.84

[a] Averaged from more than 4 devices. [b] Threshold voltage (V_{th}) is extracted from the linear fitting of $I_D^{1/2}$ vs. V_G plots. Data are shown as mean ± SD.

Supplementary Table 4. Performance summary of reported p-type OMIEC materials.

Material	μ ($cm^2\ V^{-1}\ s^{-1}$)	C^* ($F\ cm^{-3}$)	μC^* ($F\ cm^{-1}\ V^{-1}\ s^{-1}$)	Reference
g2T-T	0.28	220	61.6	13
P(g2T-TT)	1.27	240	261	18
pgBTTT	3.44	164	564.16	19
ProDOT(OE)-DMP	0.063	111	6.993	20
P3MEEMT	0.329	294	96.7	21
PEDOT:PSS + DEMTA	2.17	50.4	109.368	22
p(g2T2-T)	$\sim 10^{-4}$	8	9	23
p(g3T2-T)	0.16	211	135	23
p(g4T2-T)	0.06	192	54	23
PTDPP-DT	1.1	224	149	24
p($g_4$2T-T)	0.39	258	86	25
p[p($g_4$2T-T)-*co*-U]	0.15	279	36	25
P(aDTDPP-bis-EDOT)	0.095	21.5	2.04	26
P(gDTDPP-bis-EDOT)	0.087	189.6	16.5	26
P(gDTDPP-aBT)	0.044	24.73	1.08	26
PDPP$[T]_2${TEG}-EDOT	0.084	167	14	27
PDPP$[T]_2${TEG}3-MEET	0.133	338	45	27
P(bgDPP-T)	1.59	3.7	6	28
P(bgDPP-T2)	0.5	84.1	42	28
P(lgDPP-MeOT2)	2.15	80.8	174	28
P(bgDPP-MeOT2)	1.63	120.0	195	28
p(g2T2)	0.03	187	5	29
p(g2T2-g3T2)	2.35	161	378	29
p(g3T2)	1.04	122	127	29
p(g3T2)	0.9	156	161	30
p(g2T2-g4T2)	1.72	187	522	30
p(g1T2-g5T2)	2.61	133	496	30

Material	μ ($cm^2\ V^{-1}\ s^{-1}$)	C^* ($F\ cm^{-3}$)	μC^* ($F\ cm^{-1}\ V^{-1}\ s^{-1}$)	Reference
p(g0T2-g6T2)	2.95	74	302	30
p(gDPP-TT)	0.57	184	104.88	31
p(gDPP-T2)	1.55	196	303.8	31
p(gDPP-MeOT2)	0.28	169	47.32	31
PgBT(F)2gT	0.060	74	4.44	14
PgBT(F)2gTT	0.931	170	158.27	14
TDPP-gTVT	1.1	173.5	205.2	32
TDPP-gTBTT	0.18	122.5	21.5	32
p(p2T-TT)	1.76	103.21	182.24	33
p(b2T-TT)	3.03	113.93	342.2	33
PEDOT-Phos	2.83×10^{-5}	282	0.008	34
P3gCPDT-2gT2	0.66	140	92.4	35
P3gCPDT-1gT2	0.90	320	288.6	35
P3gCPDT-MeOT2	3.1	145	448.6	35
IG-T	2.7×10^{-3}	76.2	0.2	36
TIG-T	1.1	121	132	36
TIG-BT	0.61	82.1	55	36
P3APPT	0.38	81	30.5	37
P3AAPT	0.36	25	9.2	37
TIIP	0.05	32	1.75	38
p(g3T2-T) + CB + BCF	3.35	166	556.31	39
PBBTL	0.034	143	4.82	40
P(gTDPPT)	0.40	161	65.1	41
G2-DMP	0.32	227	73	42
G3-DMP	0.26	214	57	42
G4-DMP	0.94	213	200	42
PProDOT-DPP	0.88	305	269	43
PgBT(Ion)2gTT	1.02	143	145.3	44
PgBT(TriEG)2gTT	0.26	147	39.37	44

Material	μ (cm^2 V^{-1} s^{-1})	C^* (F cm^{-3})	μC^* (F cm^{-1} V^{-1} s^{-1})	Reference
g2T2-gBT2	0.18	224	40	45
g2T2-gBT4	0.99	364	359	45
g2T2-gBT6	0.47	435	203	45
inDTP-P	0.26	147	38	46
outDTP-P	0.61	113	69	46
inDTP-T	1.65	162	267	46
outDTP-T	0.38	117	45	46
inDTP-2T	0.68	168	115	46
outDTP-2T	0.39	166	65	46
PDPP-2EG	0.21	97.89	20	47
PDPP-3EG	4.32	113.58	491	47
PDPP-4EG	6.52	107.72	702	47
PDPP-5EG	3.20	107.88	346	47
PE2gT	0.27	313	84.2	48
PT2gT	1.0	290	290	48
PT2gTT	0.277	221	61.2	48
p(g_4T2-TT)	3.13	120	374	49
p(g_4T2-TT) [a]	6.53	308	2008	49
gFBT-T	0.0097	25	0.00024	50
gFBT-TT	0.037	31	1.15	50
gFBT-2T	0.03	133	4.03	50
gFBT-g2T	5.76	147	847	50
gFBT-3g2T	0.69	292	202	50
Pu2gT(24C8)	2.83	337.5	955	This work

[a] The material was fractionated after synthesis using preparative gel permeation chromatography to control the concentration of residual palladium catalyst.

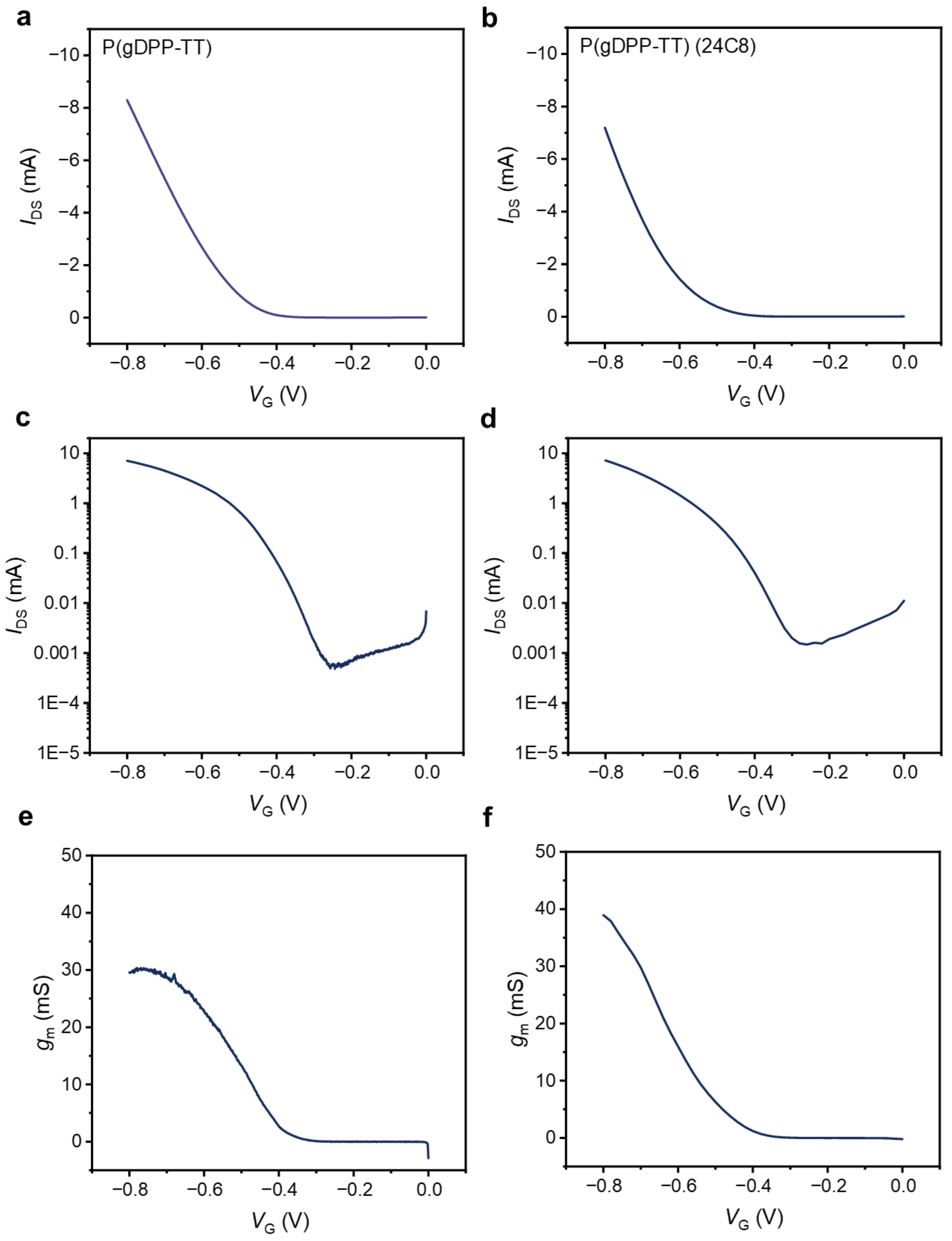

Supplementary Figure 36. Universality verification. a,**b**, Transfer characteristics of P(gDPP-TT) (**a**), P(gDPP-TT) (24C8) (**b**) in aqueous $NaPF_6$. **c**,**d**, Log transfer characteristics of P(gDPP-TT) (**a**), P(gDPP-TT) (24C8) (**b**) in aqueous $NaPF_6$. **e**,**f**, g_m curves of P(gDPP-TT) (**a**), P(gDPP-TT) (24C8) (**b**) in aqueous $NaPF_6$.

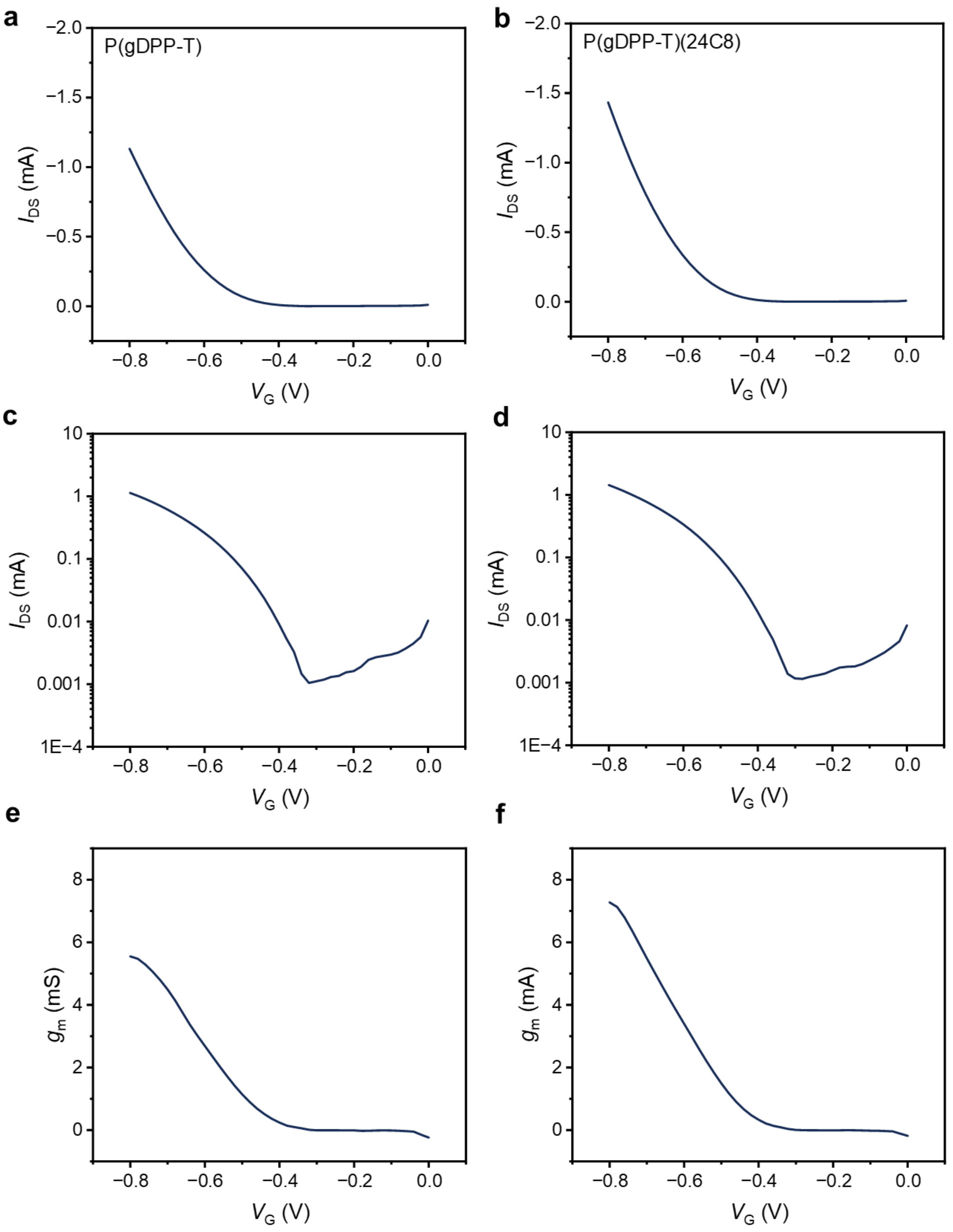

Supplementary Figure 37. Universality verification. a,**b**, Transfer characteristics of P(gDPP-T) (**a**), P(gDPP-T) (24C8) (**b**) in aqueous $NaPF_6$. **c**,**d**, Log transfer characteristics of P(gDPP-T) (**a**), P(gDPP-T) (24C8) (**b**) in aqueous $NaPF_6$. **e**,**f**, g_m curves of P(gDPP-T) (**a**), P(gDPP-T) (24C8) (**b**) in aqueous $NaPF_6$.

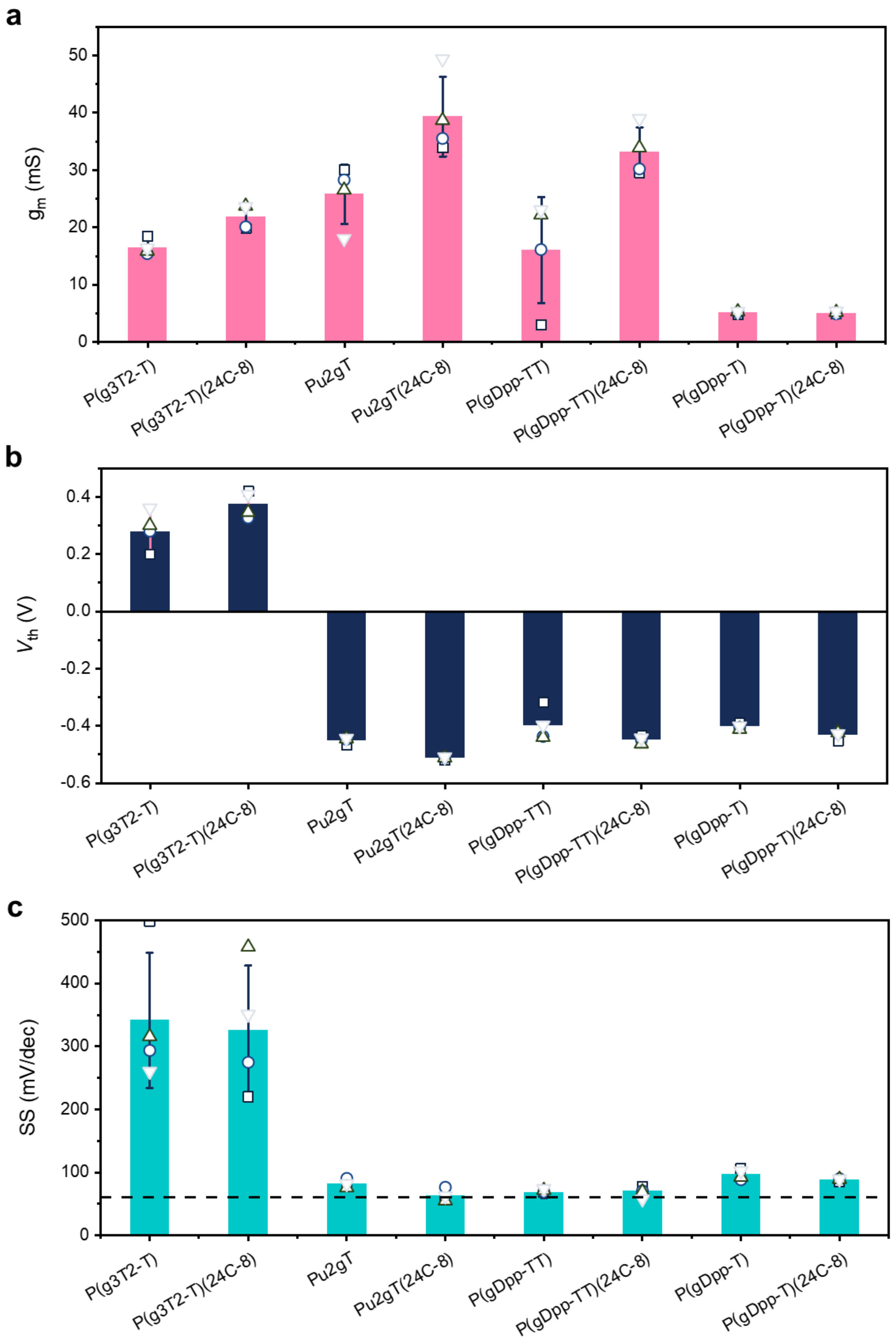

Supplementary Figure 38. Statistical Comparison. Transconductance(a), Threshold Voltages(b) and Subthreshold Slopes(c) for different OMIECs. Data are shown as mean ± SD with individual data points overlaid.

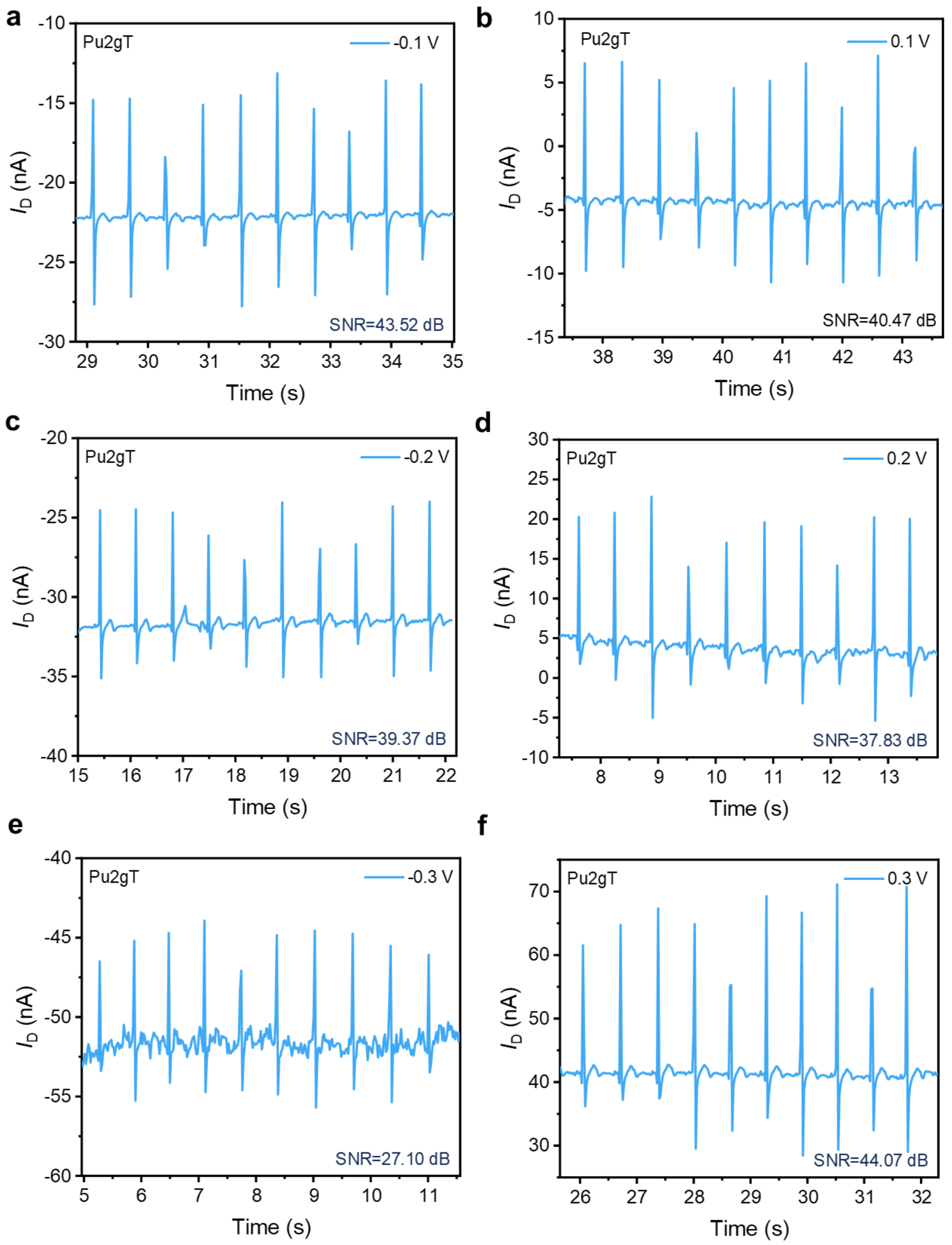

Supplementary Figure 39. ECG signal testing. Current response and signal-to-noise ratio (SNR) of Pu2gT OECT devices during electrocardiogram (ECG) signal acquisition in different gate voltage.

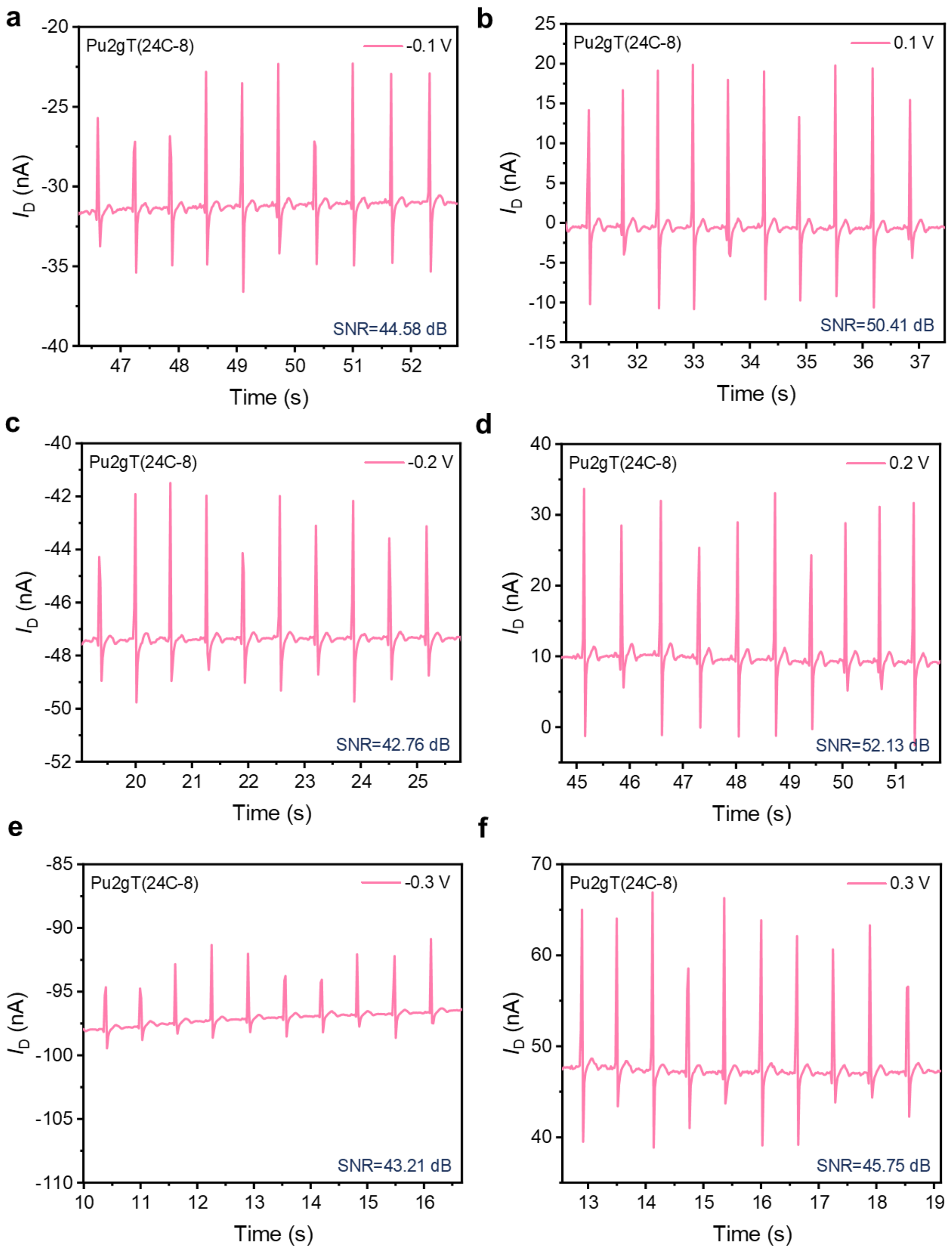

Supplementary Figure 40. ECG signal testing. Current response and signal-to-noise ratio (SNR) of Pu2gT(24C8) OECT devices during electrocardiogram (ECG) signal acquisition in different gate voltage.

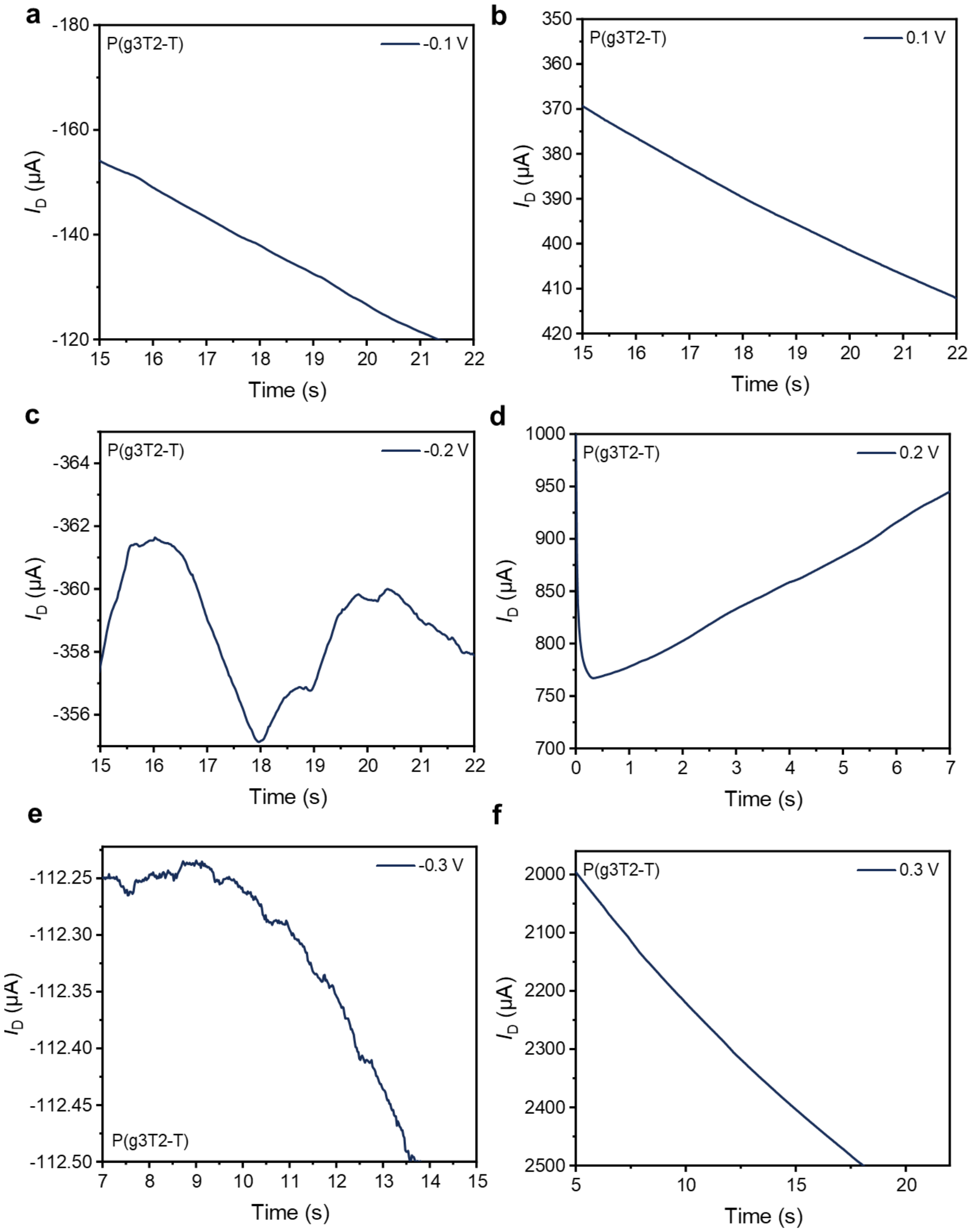

Supplementary Figure 41. ECG signal testing. Current response and signal-to-noise ratio (SNR) of P(g3T2-T) OECT devices during electrocardiogram (ECG) signal acquisition in different gate voltage.

Supplementary References

1 Barducci, A., Bussi, G. & Parrinello, M. Well-tempered metadynamics: a smoothly converging and tunable free-energy method. *Phys. Rev. Lett.* **100**, 020603 (2008).

2 Siemons, N. et al. Controlling swelling in mixed transport polymers through alkyl side-chain physical cross-linking. *Proc. Natl. Acad. Sci. U S A.* **120**, e2306272120 (2023).

3 Siemons, N. Forcefield for modelling mixed transport polymers (Version 1.0.0) (2023) https://github.com/nicholas9182/ForceField_for_Conjugated_Polymers.

4 Breneman, C. M. & Wiberg, K. B. Determining atom-centered monopoles from molecular electrostatic potentials. The need for high sampling density in formamide conformational analysis. *J. Comput. Chem.* **11**, 361–373 (2004).

5 Izadi, S., Anandakrishnan, R. & Onufriev, A. V. Building water models: a different approach. *J. Phys. Chem. Lett.* **5**, 3863–3871 (2014).

6 Van Der Spoel, D. et al. GROMACS: fast, flexible, and free. *J. Comput. Chem.* **26**, 1701–1718 (2005).

7 Bonomi, M. et al. PLUMED: a portable plugin for free-energy calculations with molecular dynamics. *Comput. Phys. Commun.* **180**, 1961–1972 (2009).

8 Bussi, G., Donadio, D. & Parrinello, M. Canonical sampling through velocity rescaling. *J. Chem. Phys.* **126**, 014101 (2007).

9 Berendsen, H. J. C., van der Spoel, D. & van Drunen, R. GROMACS: a message-passing parallel molecular dynamics implementation. *Comput. Phys. Commun.* **91**, 43–56 (1995).

10 Branduardi, D., Bussi, G. & Parrinello, M. Metadynamics with adaptive Gaussians. *J. Chem. Theory. Comput.* **8**, 2247–2254 (2012).

11 Hexemer, A. et al. A SAXS/WAXS/GISAXS beamline with multilayer monochromator. *J. Phys. Conf. Ser.* **247**, 012007 (2010).

12 Ilavsky, J. Nika: software for two-dimensional data reduction. *J. Appl. Crystallogr.* **45**, 324–328 (2012).

13 Nielsen, C. B. et al. Molecular design of semiconducting polymers for high-performance organic electrochemical transistors. *J. Am. Chem. Soc.* **138**, 10252–10259 (2016).

14 Ding, B. et al. Influence of backbone curvature on the organic electrochemical transistor performance of glycolated donor–acceptor conjugated polymers. *Angew. Chem. Int. Ed.* **60**, 19679–19684 (2021).

15 Randles, J. E. B. A cathode ray polarograph. Part II.—The current-voltage curves. *Trans. Faraday Soc.* **44**, 327–338 (1948).

16 Ševčík, A. Oscillographic polarography with periodical triangular voltage. *Collect. Czech. Chem. Comm.* **13**, 349–377 (1948).

17 Lin, S., Usov, P. M. & Morris, A. J. The role of redox hopping in metal-organic framework electrocatalysis. *Chem. Commun.* **54**, 6965–6974 (2018).

18 Giovannitti, A. et al. Controlling the mode of operation of organic transistors through side-chain engineering. *Proc. Natl. Acad. Sci. U S A.* **113**, 12017–12022 (2016).

19 Hallani, R. K. et al. Regiochemistry-driven organic electrochemical transistor performance enhancement in ethylene glycol-functionalized polythiophenes. *J. Am. Chem. Soc.* **143**, 11007–11018 (2021).

20 Savagian, L. R. et al. Balancing charge storage and mobility in an oligo(ether) functionalized dioxythiophene copolymer for organic- and aqueous-based electrochemical devices and transistors. *Adv. Mater.* **30**, 1804647 (2018).

21 Flagg, L. Q. et al. Polymer crystallinity controls water uptake in glycol side-chain polymer organic electrochemical transistors. *J. Am. Chem. Soc.* **141**, 4345–4354 (2019).
22 Keene, S. T. et al. Enhancement-mode PEDOT:PSS organic electrochemical transistors using molecular de-doping. *Adv. Mater.* **32**, 2000270 (2020).
23 Moser, M. et al. Ethylene glycol-based side chain length engineering in polythiophenes and its impact on organic electrochemical transistor performance. *Chem. Mater.* **32**, 6618–6628 (2020).
24 Wu, X. et al. Enhancing the electrochemical doping efficiency in diketopyrrolopyrrole-based polymer for organic electrochemical transistors. *Adv. Electron. Mater.* **7**, 2000701 (2020).
25 Zokaei, S. et al. Toughening of a soft polar polythiophene through copolymerization with hard urethane segments. *Adv. Sci.* **8**, 2002778 (2021).
26 Wang, N. et al. Ethylenedioxythiophene incorporated diketopyrrolopyrrole conjugated polymers for high-performance organic electrochemical transistors. *J. Mater. Chem. C.* **9**, 4260–4266 (2021).
27 Krauss, G. et al. Polydiketopyrrolopyrroles carrying ethylene glycol substituents as efficient mixed ion-electron conductors for biocompatible organic electrochemical transistors. *Adv. Funct. Mater.* **31**, 2010048 (2021).
28 Jia, H. et al. Engineering donor–acceptor conjugated polymers for high-performance and fast-response organic electrochemical transistors. *J. Mater. Chem. C.* **9**, 4927–4934 (2021).
29 Moser, M. et al. Controlling electrochemically induced volume changes in conjugated polymers by chemical design: from theory to devices. *Adv. Funct. Mater.* **31**, 2100723 (2021).
30 Moser, M. et al. Side chain redistribution as a strategy to boost organic electrochemical transistor performance and stability. *Adv. Mater.* **32**, 2002748 (2020).
31 Moser, M. et al. Polaron delocalization in donor–acceptor polymers and its impact on organic electrochemical transistor performance. *Angew. Chem. Int. Ed.* **133**, 7856–7864 (2021).
32 Wang, Y. et al. The effect of the donor moiety of DPP based polymers on the performance of organic electrochemical transistors. *J. Mater. Chem. C.* **9**, 13338–13346 (2021).
33 Moser, M. et al. Propylene and butylene glycol: new alternatives to ethylene glycol in conjugated polymers for bioelectronic applications. *Mater. Horiz.* **9**, 973–980 (2022).
34 Hopkins, J. et al. A phosphonated poly(ethylenedioxythiophene) derivative with low oxidation potential for energy-efficient bioelectronic devices. *Chem. Mater.* **34**, 140–151 (2021).
35 Lan, L. et al. Facilely accessible porous conjugated polymers toward high-performance and flexible organic electrochemical transistors. *Chem. Mater.* **34**, 1666–1676 (2022).
36 Parr, Z. S. et al. From p- to n-type mixed conduction in isoindigo-based polymers through molecular design. *Adv Mater.* **34**, 2107829 (2022).
37 Chen, S. E. et al. Impact of varying side chain structure on organic electrochemical transistor performance: a series of oligoethylene glycol-substituted polythiophenes. *J. Mater. Chem. A/* **10**, 10738–10749 (2022).
38 Rashid, R. B. et al. A semiconducting two-dimensional polymer as an organic electrochemical transistor active layer. *Adv. Mater.* **34**, 2110703 (2022).
39 Hidalgo Castillo, T. C. et al. Simultaneous performance and stability improvement of a p-type organic electrochemical transistor through additives. *Chem. Mater.* **34**, 6723–6733 (2022).
40 Wu, X. et al. All-polymer bulk-heterojunction organic electrochemical transistors with balanced ionic and electronic transport. *Adv. Mater.* **34**, 2206118 (2022).
41 Li, P., Shi, J., Lei, Y., Huang, Z. & Lei, T. Switching p-type to high-performance n-type organic electrochemical transistors via doped state engineering. *Nat. Commun.* **13**, 5970 (2022).

42 DiTullio, B. T. et al. Effects of side-chain length and functionality on polar poly(dioxythiophene)s for saline-based organic electrochemical transistors. *J. Am. Chem. Soc.* **145**, 122–134 (2023).
43 Luo, X. et al. Designing donor–acceptor copolymers for stable and high-performance organic electrochemical transistors. *ACS Macro Lett.* **10**, 1061–1067 (2021).
44 Ding, B. et al. Enhanced organic electrochemical transistor performance of donor–acceptor conjugated polymers modified with hybrid glycol/ionic side chains by postpolymerization modification. *Chem. Mater.* **35**, 3290–3299 (2023).
45 Cong, S. et al. Tunable control of the performance of aqueous-based electrochemical devices by post-polymerization functionalization. *Mater. Horiz.* **10**, 3090–3100 (2023).
46 Halaksa, R. et al. The influence of regiochemistry on the performance of organic mixed ionic and electronic conductors. *Angew. Chem. Int. Ed.* **62**, e202304390 (2023).
47 Jo, I. Y. et al. High-performance organic electrochemical transistors achieved by optimizing structural and energetic ordering of diketopyrrolopyrrole-based polymers. *Adv. Mater.* **36**, 2307402 (2024).
48 Ding, B. et al. Development of synthetically accessible glycolated polythiophenes for high-performance organic electrochemical transistors. *Adv. Electron. Mater.* **10**, 2300580 (2024).
49 Griggs, S. et al. The effect of residual palladium on the performance of organic electrochemical transistors. *Nat. Commun.* **13**, 7964 (2022).
50 H. Liao, A. et al. High Performance Organic Mixed Ionic-Electronic Polymeric Conductor with Stability to Autoclave Sterilization. *Angew. Chem. Int. Ed.* **64**, e202416288 (2025).
