# Supplementary material for: Delayed cation dynamics enables dual-doped organic electrochemical transistors with high current sensitivity
Source: Nat Commun. 2026 Jun 1;17:7029. doi: 10.1038/s41467-026-73762-1 (PMC13392116; doi:10.1038/s41467-026-73762-1)
Supplement: Supplementary file 2 — Transparent Peer Review file [file 41467_2026_73762_MOESM2_ESM.pdf]

nature portfolio

Peer Review File

Delayed Cation Dynamics Enables Dual-Doped Organic Electrochemical Transistors with High Current Sensitivity

Corresponding Author: Professor Wei Ma

This file contains all reviewer reports in order by version, followed by all author rebuttals in order by version.

Version 0:

Reviewer comments:

Reviewer #1

(Remarks to the Author)
The authors have responded to my previous comments.

Reviewer #4

(Remarks to the Author)
Authors have demonstrated an OECT in which they deep-dive into an important doping mechanism which this reviewer feels complements and adds nuance to previously reported studies (10.1021/acs.chemmater.8b02220, 10.1021/acsmaterialslett.9b00501, 10.1002/adfm.201807034 etc). The manuscript systematically investigates the critical role of ions on the “dual-doping” mechanism. The study concludes that properties of OECTs are fundamentally controlled by the coupled interaction between the polymer and the electrolyte ion/solvent, specifically showing how simultaneous anion injection/cation expulsion drastically changes the device performance. The reviewer feels that the authors provide a well written manuscript with a breadth of characterizations and unify the principles reported in previous works. Although the core concepts are not entirely novel, the manuscript presents a well-executed investigation into the “dual-doping” mechanism and yields compelling results and the findings are useful for the community. The manuscript may be accepted for publication after minor revision
1. A core issue I see is that the device's characteristics (once the material system is fixed) are a function of how fast the gate voltage is changed, due to the different diffusion coefficients of the two ion species. This limits the applications to the transduction of those signals that overlap with the rate that maximizes the dual-doping. Neuromorphic devices may be a better demonstration as an application, but the reviewer acknowledges it is beyond the scope of the manuscript
2. When both cation expulsion and anion injection are involved, the transient behavior would be affected by two time constants. Mixed pathways typically reduce usable bandwidth and make them strongly dependent on the bias history because ion redistribution is not fast. How does the transient response look like?
3. Other than side chain engineering, geometric scaling (specifically thickness) may reduce the diffusion path length and therefore may allow the slow anion kinetics to keep up with the cation expulsion at faster scan rate. Other strategies like electrolytes with less solvated anions, solid electrolytes that intentionally slow down cation expulsion component etc may also result in dual doping system over a wider bandwidth
4. As demonstrated by the authors in the manuscript, Vth shift would indeed be dependent on the scan rate. But the reviewer feels that the devices may exhibit a bias-stress drift because the ionic redistribution is slow
5. The SS improvement may be observable only in cases where the faster mechanism dominates at the onset of conduction, not necessarily in all dual-doping scenarios
6. What are the on/off ratios observed in these devices? The off current depends on residual charges within the film among other physical drivers. Do you see a drift in the ratio over a large number of cycles?
7. Other minor comments are below
a. The abbreviated form c-OECT is not expanded in the main text
b. Line 148, Page 6. Authors are incorrectly referring to fig 3a in stead of fig 2a. Same mistake is continued in the remainder of that paragraph and the next two. Please make sure the manuscript is consistent
c. Figure S27. The encircled regions indicating ion expulsion and uptake is not labeled
d. Figure S38. Different what voltages? Drain? Gate? Clarify

Version 1:

Reviewer comments:

Reviewer #4

(Remarks to the Author)
The manuscript has now been revised in response to the comments provided by me for the previous version

Open Access This Peer Review File is licensed under a Creative Commons Attribution 4.0 International License, which permits use, sharing, adaptation, distribution and reproduction in any medium or format, as long as you give appropriate credit to the original author(s) and the source, provide a link to the Creative Commons license, and indicate if changes were made.
In cases where reviewers are anonymous, credit should be given to 'Anonymous Referee' and the source.
The images or other third party material in this Peer Review File are included in the article's Creative Commons license, unless indicated otherwise in a credit line to the material. If material is not included in the article's Creative Commons license and your intended use is not permitted by statutory regulation or exceeds the permitted use, you will need to obtain permission directly from the copyright holder.
To view a copy of this license, visit https://creativecommons.org/licenses/by/4.0/

REVIEWER COMMENTS

Reviewer #1 (Remarks to the Author):

The authors have responded to my previous comments.

Response: Thank you for your time to review our revised manuscript and for your positive comment. We appreciate your valuable feedback on our previous version, which has significantly helped us improve the quality and clarity of our work. We are pleased that our responses and revisions have addressed your concerns.

Reviewer #4 (Remarks to the Author):

Authors have demonstrated an OECT in which they deep-dive into an important doping mechanism which this reviewer feels complements and adds nuance to previously reported studies (10.1021/acs.chemmater.8b02220, 10.1021/acsmaterialslett.9b00501, 10.1002/adfm.201807034 etc). The manuscript systematically investigates the critical role of ions on the "dual-doping" mechanism. The study concludes that properties of OECTs are fundamentally controlled by the coupled interaction between the polymer and the electrolyte ion/solvent, specifically showing how simultaneous anion injection/cation expulsion drastically changes the device performance. The reviewer feels that the authors provide a well written manuscript with a breadth of characterizations and unify the principles reported in previous works. Although the core concepts are not entirely novel, the manuscript presents a well-executed investigation into the "dual-doping" mechanism and yields compelling results and the findings are useful for the community. The manuscript may be accepted for publication after minor revision

1. A core issue I see is that the device's characteristics (once the material system is fixed) are a function of how fast the gate voltage is changed, due to the different diffusion coefficients of the two ion species. This limits the applications to the transduction of those signals that overlap with the rate that maximizes the dual-doping. Neuromorphic devices may be a better demonstration as an application, but the reviewer acknowledges it is beyond the scope of the manuscript.

Response: Thank you for this insightful comment. We fully agree with your observation that the device characteristics are indeed influenced by the rate of gate voltage variation, owing to the distinct diffusion coefficients of the two ionic species. In the present work, we have accordingly focused on two representative application scenarios aligned with this behavior. First, given that the device is modulated by the dynamic processes of both ionic species, it displays enhanced sensitivity toward ion concentration, which we have successfully applied to ion concentration sensing. Second, consistent with your comment, we have also demonstrated the device for detecting signals whose rates match the optimal window for dual-doping, in line with the operational regime highlighted in this study.

We fully agree with your suggestion that this property could serve as an excellent demonstration for neuromorphic devices, and we plan to actively explore this direction in the future. It is worth noting that in our p-type organic semiconductor (Pu2gT), the delayed expulsion of cations might pose certain challenges for neuromorphic applications. For example, the long-term retention of cations within a P-type OMIEC (organic mixed ionic-electronic conductor), where they act as dedopants, may affect the memory retention effect of neuromorphic devices, and this effect can also be influenced by the doping history. Of course, we believe that by designing n-type organic semiconductors with similar side chains to regulate cation dynamics, the memory effects in n-type devices could be significantly enhanced.

2. When both cation expulsion and anion injection are involved, the transient behavior would be affected by two time constants. Mixed pathways typically reduce usable bandwidth and make them strongly dependent on the bias history because ion redistribution is not fast. How does the transient response look like?

Response: Thank you for your comments regarding the transient response. We fully agree with your observation that the dual-doping process involves the kinetics of two ionic species and should therefore be governed by two distinct time constants. To address this, we have performed additional experimental and detailed analyses.

First, Figure R1 shows the response speed of the planar Pu2gT device. As can be seen, the transient response of Pu2gT clearly exhibits two distinct phases, reflecting the dual kinetic processes of anions and cations. Due to hindered cation expulsion, the response shows a slower time characteristic. We fitted the data using both single-exponential and double-exponential models. The results indicate that the single-exponential fit is poor, whereas the double-exponential fit closely matches the experimental data, confirming that the process indeed follows a dual-time-constant model.

As shown in Figure R1, the transient response of the planar Pu2gT OECT device displays two clearly distinguishable kinetic phases, corresponding to anion injection and cation expulsion, respectively. The slower component arises from the hindered expulsion of cations due to strong coordination with the polymer side chains. We quantitatively evaluated the dynamics using both single- and double-exponential fitting models. The single-exponential model yielded unsatisfactory agreement with the experimental data, as the fitting curve fails to converge (Figure R1a). In comparison, the double-exponential model provided excellent fit (R^2 = 0.99), directly validating the presence of two distinct time constants in the dual-doping process (Figure R1b).

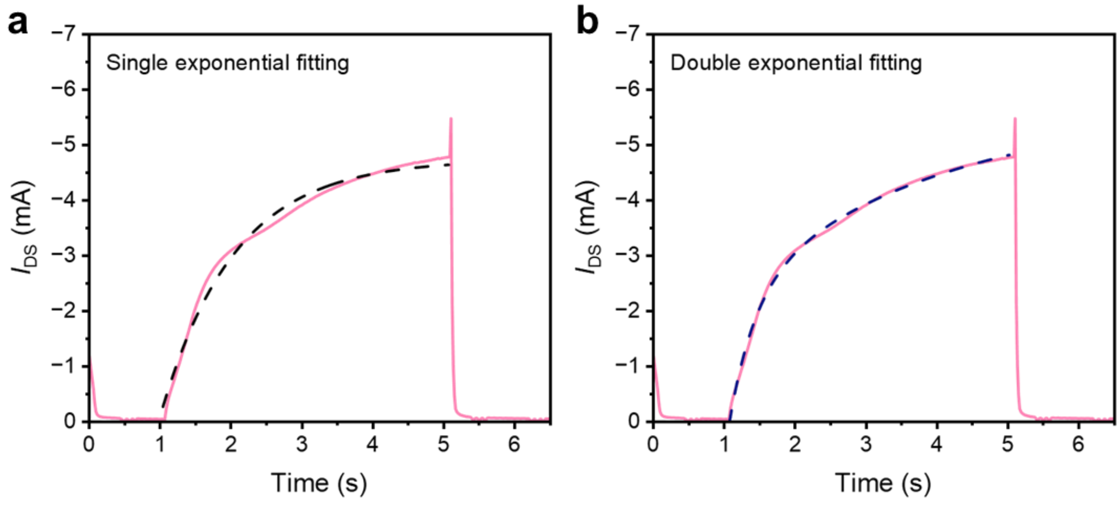

Figure R1. Transient current response of a Pu2gT-based planar OECT device, fitted using single- and double-exponential models. Under the single-exponential model, the fitting curve fails to converge. The double-exponential model gives a satisfactory R^2 of 0.99. Test conditions: V_D = −0.6 V, V_G = −0.7 V.

We also fully agree with your comment that the transient behavior is strongly dependent on doping history, particularly due to the slow redistribution of cations within the polymer film. To investigate this effect, we conducted experiments by applying consecutive voltage pulses and monitored the transient response of the OECT (Figure R2). Under the first pulse, when the film had no doping history, the device exhibited pronounced dual-time-constant behavior: fast anion injection and slow cation expulsion. After seven consecutive pulses, the previously expelled cations lacked sufficient time to fully re-coordinate with the polymer side chains, resulting in a less constrained ionic environment and faster cation expulsion. As evident in Figures R2b and R2c, the device response accelerated noticeably with increasing pulse number. We further

extracted the two time constants at different pulse numbers (Figure R2d). With increasing pulses, τ_1 (corresponding to anions) remained largely unchanged, consistent with the absence of strong coordination effects for anions. In contrast, τ_2 (corresponding to cations) decreased significantly, supporting our interpretation of accelerated cation dynamics.

In summary, our experimental results confirm that the dual-doping process is characterized by two distinct time constants and that the transient response is indeed modulated by doping history, in excellent agreement with your observations.

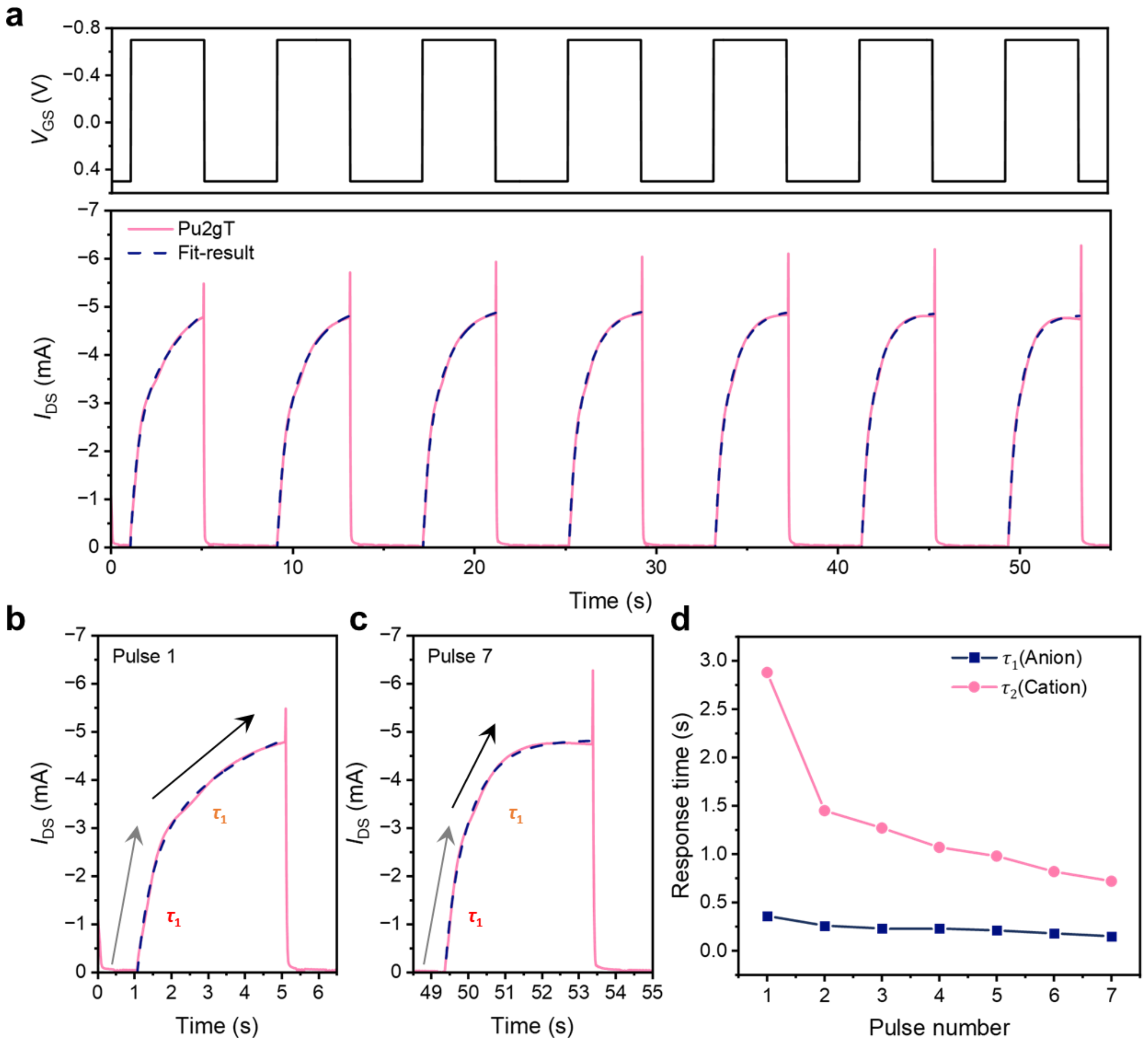

Figure R2. Transient current response of a Pu2gT-based planar OECT under sequential voltage pulses. a, Responses to multiple pulses, showing that the second and subsequent pulses are influenced by the doping history established by preceding pulses. **b, c,** Transient current traces for the first pulse and the seventh pulse, respectively. A clear two-stage process is observed for the first pulse, whereas the second stage becomes notably faster for the seventh. **d,** Time constants extracted from double-exponential fitting, where τ_1 and τ_2 are attributed to the anion- and cation-related processes, respectively.

3. Other than side chain engineering, geometric scaling (specifically thickness) may reduce the diffusion path length and therefore may allow the slow anion kinetics to keep up with the cation expulsion at faster scan rate.

Other strategies like electrolytes with less solvated anions, solid electrolytes that intentionally slow down cation expulsion component etc may also result in dual doping system over a wider bandwidth

Response: Thank you for your suggestions on additional strategies to optimize the dual-doping behavior in OECTs. We fully agree that film thickness, ion solvation strength, and the use of solid electrolytes with tailored ion dynamics can strongly modulate the kinetic matching between anion injection and cation expulsion, thereby extending the operational bandwidth of dual-doping systems. To address these points, we have performed supplementary experiments and included relevant discussions.

First, we investigated the influence of film thickness using planar Pu2gT OECT devices with channel thicknesses ranging from 70 to 120 nm. The device current increases with thicker films, consistent with the larger amount of electroactive material. Meanwhile, the hysteresis in the transfer curves becomes increasingly pronounced at greater thicknesses (Figure R3a), indicative of slower overall ion kinetics. In our dual-doping mechanism, anion injection proceeds relatively readily, while cation expulsion is hindered by coordination with polymer side chains. As the film becomes thicker, the kinetic imbalance is further amplified. As illustrated in Figure R3b, thicker films display a delayed current onset but a sharper current transition, consistent with enhanced dual-doping characteristics and increased hysteresis.

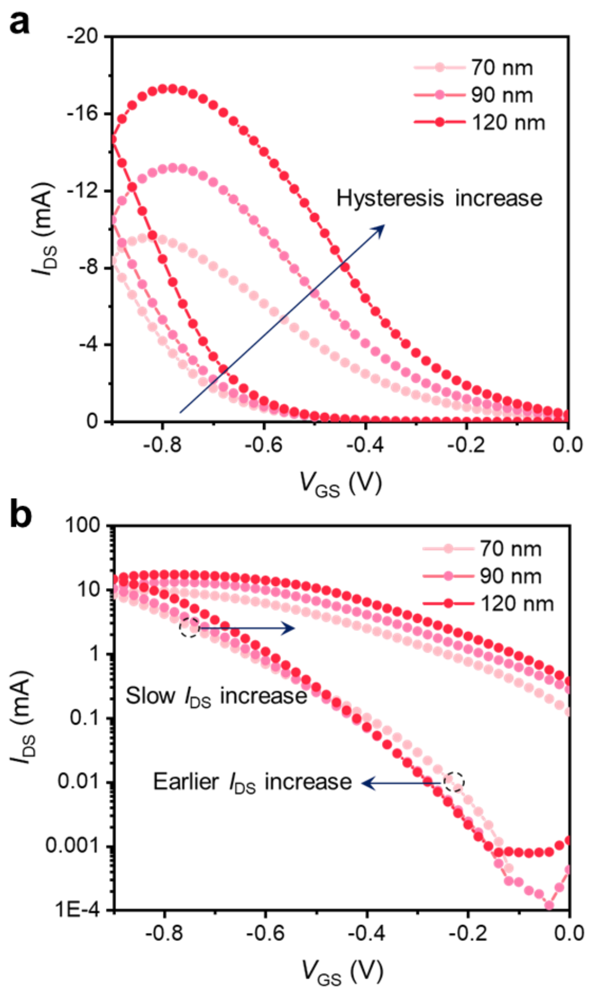

Figure R3. Transfer characteristics of planar Pu2gT devices with different film thicknesses (70, 90, and 120 nm) at a scan rate of 1 V s^{-1}.

Second, we examined the effect of ion solvation by comparing NaSCN electrolyte with $NaPF_6$ used in the manuscript. As previous reported, $NaPF_6$ is one of the least solvated electrolyte (*ACS Nano* **2023**, *17*, 18608–18615), i.e. NaSCN is more solvated than $NaPF_6$ in the aqueous solution. As shown in Figure R4, the NaSCN electrolyte yields considerably lower current, a much smaller threshold voltage shift, and no enhancement of

transconductance at high scan rates. These results validate your insight that a lower degree of anion solvation (as in $NaPF_6$) effectively promotes the dual-doping effect. We note that ion size, solvation energy, and perturbation of the hydrogen-bonding network within the electrolyte may further contribute to the observed differences between NaSCN and $NaPF_6$.

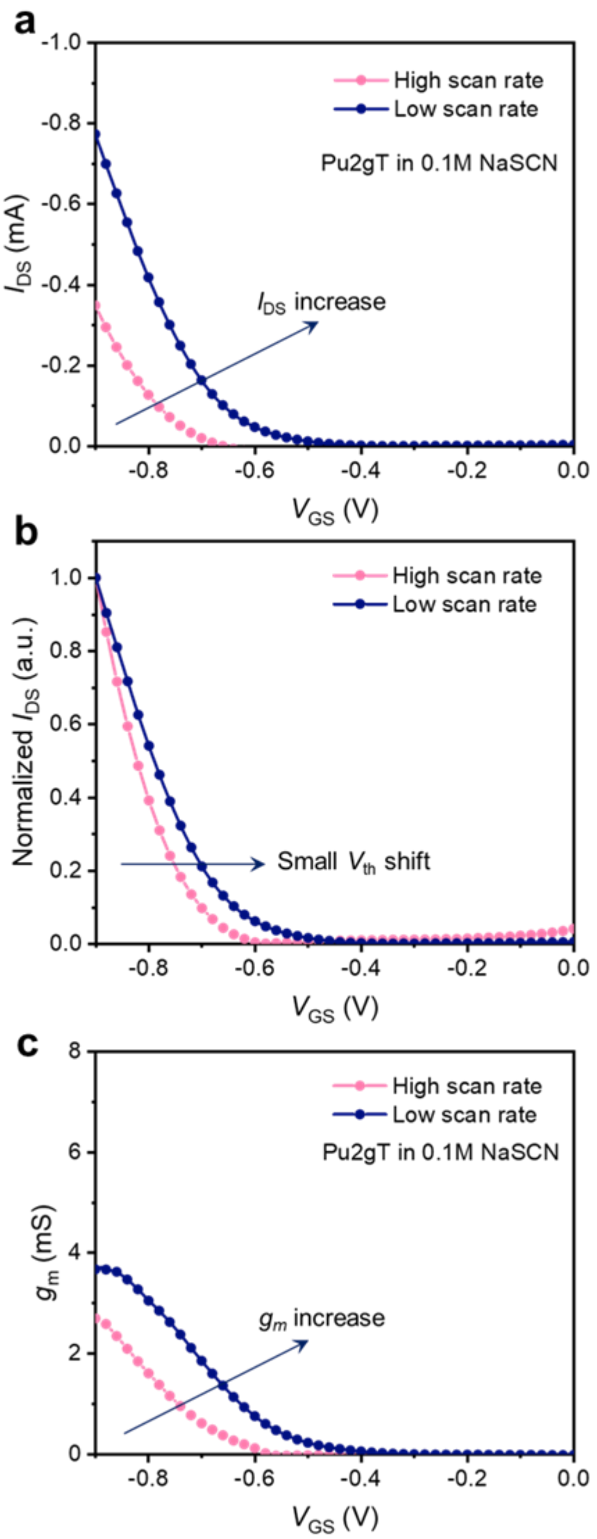

Figure R4. Transfer curves and transconductance characteristics of Pu2gT in a highly solvated 0.1 M NaSCN electrolyte at various scan rates. The high and low scan rates represent 1 and 0.05 V s^{-1}, respectively.

Finally, we evaluated the effect of solid electrolyte (EMIM:TFSI in PVDF-HFP, weight ratio = 4:1) with intrinsically low ion mobility (Figure R5). At the high scan rate (1 V s^{-1}), the device showed negligible response due to the extremely slow kinetics of both anions and cations, which severely limits efficient OECT

operation. At sufficiently slow scan rates (0.05 V s^{-1}), however, both ionic processes can proceed to a measurable extent, restoring normal device performance.

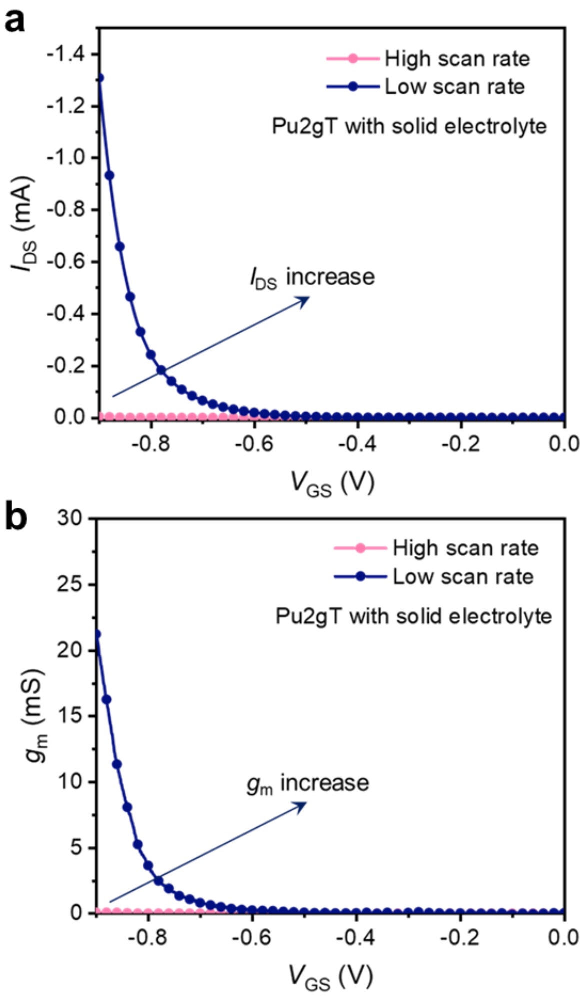

Figure R5. Transfer curves and transconductance characteristics of Pu2gT with a solid electrolyte (EMIM:TFSI in PVDF-HFP, weight ratio = 4:1) at different scan rates. The high and low scan rates represent 1 and 0.05 V s^{-1}, respectively.

In summary, film thickness, ion solvation strength, and electrolyte ion mobility all play critical roles in regulating the dual-doping process. Importantly, these parameters are strongly interrelated, and the overall device response is governed by their combined effects rather than any single variable. Further optimization of these interplaying factors is expected to broaden the operational window and practical applicability of the dual-doping mechanism.

4. As demonstrated by the authors in the manuscript, Vth shift would indeed be dependent on the scan rate. But the reviewer feels that the devices may exhibit a bias-stress drift because the ionic redistribution is slow.

Response: Thank you for this constructive comment. We fully agree with your observation that slow ionic redistribution can lead to bias-stress drift in threshold voltage, as the scanning history modulates the doping state within the polymer film. To clarify this effect, we have performed additional measurements on fresh, unbiased OECT devices to characterize threshold voltage (V_{th}) as a function of scan rate. As presented in Figure R6, these pristine devices show a clear scan-rate dependence: threshold voltages systematically increase at higher scan rates, in good agreement with the delayed cation expulsion mechanism. For a single device subjected to repeated scanning, cations are expelled during the sweep but require sufficient time to

fully re-coordinate with polymer side chains. This hysteretic behavior is fully consistent with the dual-time-constant kinetics and doping-history effects discussed in Response 2 and 6.

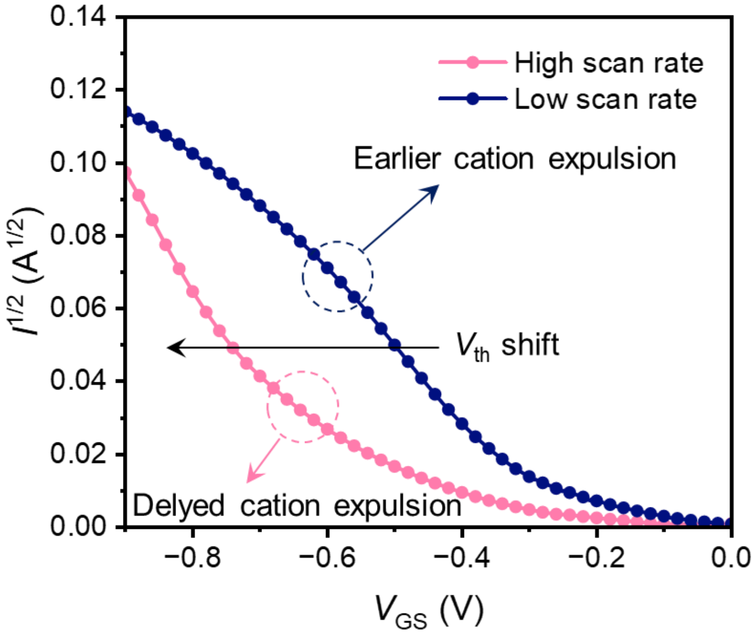

Figure R6. Threshold voltage (V_{th}) shifts in different OECT devices. Each measurement was performed on a fresh device without prior doping history. The high and low scan rates represent 1 and 0.05 V s^{-1}, respectively.

5. The SS improvement may be observable only in cases where the faster mechanism dominates at the onset of conduction, not necessarily in all dual-doping scenarios.

Response: Thank you for your constructive comment regarding the relationship between subthreshold swing (SS) and dual-doping. We fully concur with your observation. In Pu2gT, although both doping mechanisms are active at the onset of conduction, the process is clearly dominated by the faster anion kinetics. This dominant fast mechanism is precisely why we observe a noticeable improvement in SS within our dual-doping system. As you rightly pointed out, if the slower cation-driven process were to dominate instead, it would significantly suppress the current gain and thus likely preclude any SS enhancement. We greatly appreciate this insightful suggestion. Accordingly, we have incorporated relevant discussion in the revised manuscript to clarify this condition and better delineate the scope of our findings. Thank you again for this valuable input, which has helped us refine and strengthen the presentation of our work.

Thank you for this constructive comment on the relationship between subthreshold swing (SS) improvement and dual-doping kinetics. We fully agree with your observation. In the Pu2gT system, both cationic and anionic doping processes are operative at the onset of conduction, but the switching behavior is clearly dominated by the faster anion injection kinetics. It is this dominance of the faster kinetic component that gives rise to the pronounced SS improvement observed in our dual-doping mechanism. As you correctly noted, if the slower, cation-governed process were to dominate instead, the current amplification would be substantially suppressed, and the beneficial SS improvement would likely not be achieved. We have incorporated this important discussion into the revised manuscript (Page 9, Line 229-235) to better define the scope and underlying conditions of our findings.

6. What are the on/off ratios observed in these devices? The off current depends on residual charges within the film among other physical drivers. Do you see a drift in the ratio over a large number of cycles?

Response: Thank you for your constructive comments regarding the on/off ratio and cycling stability of our devices. To address these points, we have supplemented the changes in current, transconductance, and on/off ratio of the Pu2gT device under different scan cycles and doping histories, as summarized in Figure R7. Consistent with your comments, we observed significant drift in device performance under repeated scanning.

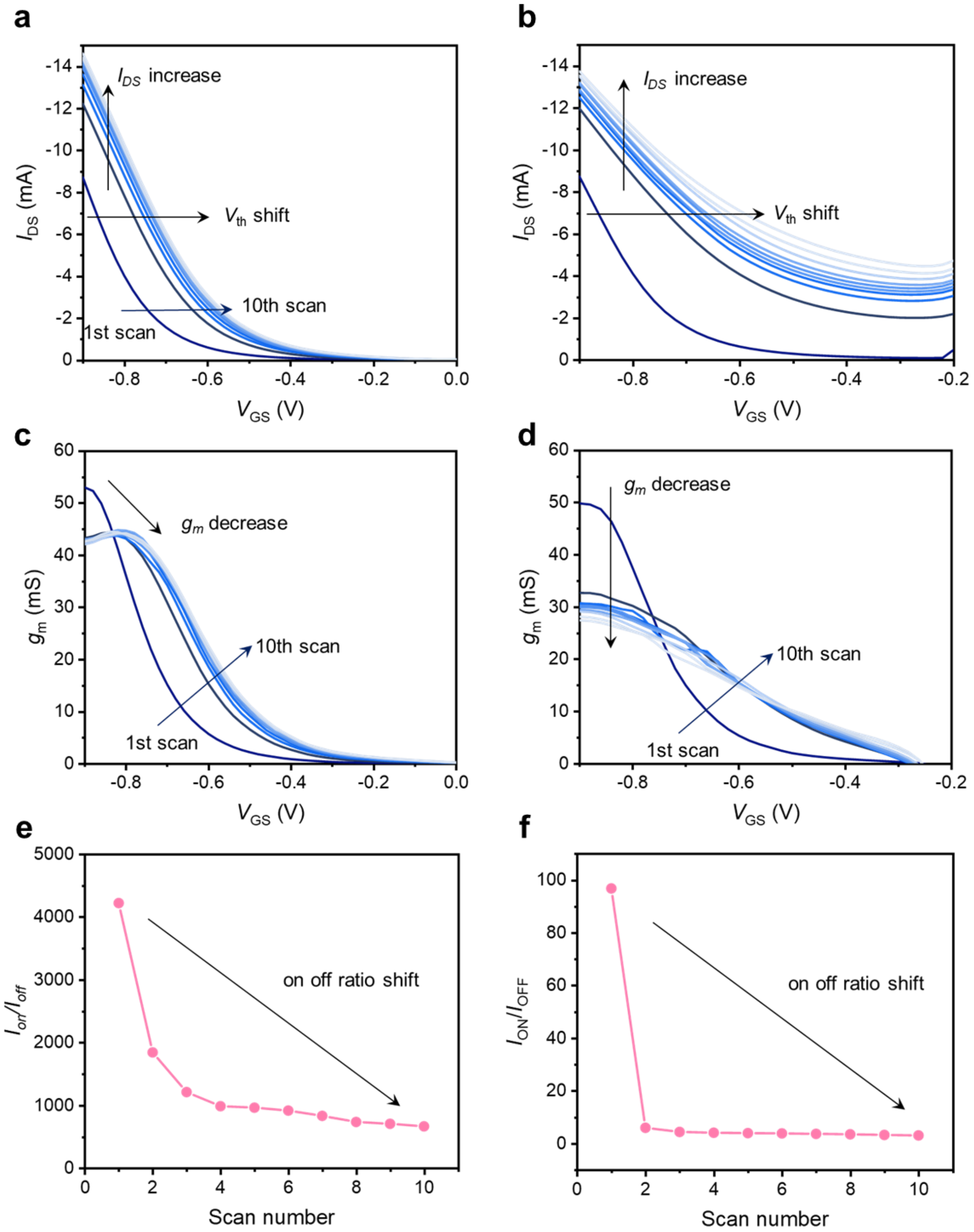

Figure R7. Variations in the current, transconductance, and on/off ratio of the planar Pu2gT OECTs over 10 consecutive scan cycles. **a, c, e,** Device characteristics when scanned to a relatively complete dedoped state. **b, d, f,** Device characteristics when scanned to an incomplete dedoped state.

Figures R7a, c, e show the device characteristics measured starting from the fully dedoped state. After the initial scan, the second to tenth scans produce a notable increase in on-state current and a negative shift in threshold voltage. Meanwhile, the transconductance decreases, and the on/off ratio degrades significantly from approximately 4×10^3 down to the order of 10^2. These changes are closely linked to the doping history of the polymer film.

As discussed earlier, a large fraction of cations is expelled during the first scan. In subsequent scans, the relatively slow re-coordination kinetics between cations and polymer side chains prevents full ionic redistribution. This weakens the dual-doping effect and favors a single-doping mechanism, leading to a reduced rate of current modulation. At the same time, the role of cations as dedopant is not fully restored, which promotes residual charge accumulation inside the film and contributes to the performance drift over multiple cycles.

Figures R7b, d, f display device characteristics under a different initial doping state, i.e. each scan starts from an incomplete dedoped state. The trends are fully consistent with the above analysis and even more pronounced, which we attribute to a higher level of residual charge remaining in the active layer.

7. Other minor comments are below
a. The abbreviated form c-OECT is not expanded in the main text
b. Line 148, Page 6. Authors are incorrectly referring to fig 3a in stead of fig 2a. Same mistake is continued in the remainder of that paragraph and the next two. Please make sure the manuscript is consistent
c. Figure S27. The encircled regions indicating ion expulsion and uptake is not labeled
d. Figure S38. Different what voltages? Drain? Gate? Clarify

Response: Thank you for your careful reading and these helpful corrections. We have addressed all points in the revised manuscript and supplementary information.

1. The abbreviated term "c-OECT" has now been defined upon its first use in the main text (Page 9, Line 215).
2. All incorrect references to Figure 3 in the relevant paragraphs have been corrected to Figure 2 (Page 6, Line 148; Page 7, Line 160, 161,167,184; Page 8, Line 195, 206).
3. In Figure S27, the encircled regions indicating ion expulsion and uptake have been clearly labeled.
4. In Figure S38, the figure caption has been updated to specify that the different curves correspond to different gate voltages**.**

REVIEWERS' COMMENTS

Reviewer #4 (Remarks to the Author):

The manuscript has now been revised in response to the comments provided by me for the previous version

Response： We thank Reviewer for the positive assessment. We are glad that the revisions have satisfactorily addressed the previous comments. We appreciate the reviewer’s time and valuable suggestions, which have helped improve the quality of our work.
